# Supplementary material for: Deep active optimization for complex systems
Source: Nat Comput Sci. 2025 Aug 25;5(9):801–12. doi: 10.1038/s43588-025-00858-x (PMC12457189; doi:10.1038/s43588-025-00858-x)
Supplement: Supplementary file 1 — Supplementary note, Figs. 1–54, Tables 1–16 and references. [file 43588_2025_858_MOESM1_ESM.pdf]

nature computational science

Supplementary information https://doi.org/10.1038/s43588-025-00858-x

# Deep active optimization for complex systems

In the format provided by the authors and unedited

# The PDF file includes:

**Supplementary Notes** ........ **3**
Brief introduction ........ 3
Pseudocode ........ 3
Evaluations on synthetic functions ........ 4
Real world tasks with easy data acquisition ........ 9
Real world task 1: Architected materials design ........ 15
Real world task 2: Compositionally complex alloy design ........ 17
Real world task 3: *De novo* cyclic peptide binder design ........ 21
Real world task 4: Designing Monoclonal antibody against SARS-COVID-2 ........ 24
**Supplementary Figures** ........ **25**
Supplementary Figure 1: Ablation study of DANTE on synthetic functions with various configurations ........ 25
Supplementary Figure 2: Distributions of the 2-D synthetic functions. ........ 26
Supplementary Figure 3: Model architecture of 1D-CNN for evaluations on synthetic functions ........ 28
Supplementary Figure 4: Evaluations on synthetic functions at initial dataset = 10 ........ 29
Supplementary Figure 5: Evaluations on synthetic functions at initial dataset = 20 ........ 30
Supplementary Figure 6: Evaluations on synthetic functions at initial dataset = 200 ........ 31
Supplementary Figure 7: Evaluations of DANTE on synthetic functions with different sampling batch sizes ........ 32
Supplementary Figure 8: Evaluations on synthetic functions using surrogate model ........ 33
Supplementary Figure 9: Evaluations on synthetic functions (Ackley, Rastrigin, Rosenbrock) using exact function ........ 34
Supplementary Figure 10: Evaluations on synthetic functions (Schwefel, Griewank, Michalewicz) using exact function ........ 35
Supplementary Figure 11: Electron ptychography reconstruction process ........ 36
Supplementary Figure 12: Phases of the Monte Carlo tree search algorithm ........ 36
Supplementary Figure 13: The key difference between UCB and DUCB. ........ 37
Supplementary Figure 14: Ablation study of DANTE on synthetic functions with different intervals ........ 38
Supplementary Figure 15: Ptychographic reconstruction procedure and the computing of normalized mean squared error. ........ 39
Supplementary Figure 16: FE simulation calibration ........ 39
Supplementary Figure 17: Model architecture of 3D-CNN for architected material property predictions ........ 40
Supplementary Figure 18: Model architecture of 1D-CNN for CCA property predictions ........ 41
Supplementary Figure 19: $R^2$ versus number of iterations while evaluations on synthetic functions using surrogate model. ........ 42
Supplementary Figure 20: Ablation study of DANTE on synthetic functions with different exploration weight ratios $c_0$ ........ 43
Supplementary Figure 21: Ablation study of DANTE on synthetic functions with different expansion actions ........ 44
Supplementary Figure 22: Evaluations on synthetic functions with only stochastic expansion ........ 45
Supplementary Figure 23: Evaluations on synthetic functions with only deterministic expansion ........ 46
Supplementary Figure 24: Dimensions versus the number of data points needed to achieve the optimum ........ 47
Supplementary Figure 25: Model performance of 3D-CNNs ........ 48
Supplementary Figure 26: Analysis of architected materials ........ 49
Supplementary Figure 27: Results of autonomous virtual laboratories (AVL)-DANTE ........ 50
Supplementary Figure 28: Model performance of 1D-CNNs utilizing DANTE for bcc CCA design ........ 50
Supplementary Figure 29: Model performance of 1D-CNNs utilizing DANTE for fcc CCA design ........ 51
Supplementary Figure 30: Model performance of 1D-CNNs utilizing MCMC for bcc CCA design ........ 52
Supplementary Figure 31: Model performance of 1D-CNNs utilizing MCMC for fcc CCA design ........ 53
Supplementary Figure 32: Calculated AHC and AHA of bcc and fcc $Fe_{1-x}Ir_x$ ........ 53
Supplementary Figure 33: Bloch spectral functions ($E = E_{Fermi}$) in the (001) plane through $\Gamma$ corresponding to minority and majority spin channels of fcc (a) $Fe_{43.5}Co_{18.5}Ni_{10}Al_{4.5}Zn_{9.5}Ir_{14}$ and (b) $Fe_{65}Ir_{35}$. ........ 54
Supplementary Figure 34: Bloch spectral functions ($E = E_{Fermi}$) in the (001) plane through $\Gamma$ corresponding to minority and majority spin channels of bcc (a) $Fe_{61.5}Co_{0.5}Ni_{0.5}Si_{2.5}Zn_{19}Ir_{16}$ and (b) $Fe_{80}Ir_{20}$. ........ 54
Supplementary Figure 35: Fitting of n(k) for CCAs ........ 55
Supplementary Figure 36: Band structures of fcc CCAs ........ 55
Supplementary Figure 37: Band structures of bcc CCAs ........ 56
Supplementary Figure 38: Interaction diagram and 3D conformation of the CK2α1−335/Pc cocrystals (PDBID: 4ib5) ........ 56
Supplementary Figure 39: RMSD of backbone atoms of peptide in native and DANTE-designed complex (PDB ID: 4ib5) ........ 57
Supplementary Figure 40: RMSF of c alpha atoms of native and DANTE-designed complex structures (PDB ID: 4ib5) ........ 57
Supplementary Figure 41: 3D interaction plot of complex with PDB ID: 7k2j ........ 58
Supplementary Figure 42: 2D interaction plot of complex with PDB ID: 7k2j ........ 58
Supplementary Figure 43: RMSD of backbone atoms of peptide in native and DANTE-designed complex (PDB ID: 7k2j) ........ 59
Supplementary Figure 44: RMSF of c alpha atoms of native and DANTE-designed complex structures (PDB ID: 7k2j) ........ 59
Supplementary Figure 45: 3D interaction plot of complex with PDB ID: 1smf. ........ 60

Supplementary Figure 46: 2D interaction plot of complex with PDB ID: 1smf. .... 60
Supplementary Figure 47: RMSD of backbone atoms of peptide in native and DANTE-designed complex (PDB ID: 1smf) .... 61
Supplementary Figure 48 RMSF of c alpha atoms of native and DANTE-designed complex structures (PDB ID: 1smf) .... 61
Supplementary Figure 49: Pipeline of designing monobody binder to SARS-COVID-2 spike protein .... 62
Supplementary Figure 50: The ipTM distributions obtained from different methods.... 62
Supplementary Figure 51: Evaluation of 1D-CNN Performance for Schwefel Function Prediction .... 63
Supplementary Figure 52: Surrogate model performance with and without top-visit sampling .... 63
Supplementary Figure 53: Evaluations on synthetic functions of DANTE equipped with various machine learning model .... 64
Supplementary Figure 54: DANTE performance on Rosenbrock-100d using surrogate model .... 65
**Supplementary Tables .... 66**
Supplementary Table 1: Setup for synthetic functions.... 66
Supplementary Table 2: Optimized reconstruction parameters by different methods .... 66
Supplementary Table 3: The convergence table of the benchmark functions. .... 66
Supplementary Table 4: Detailed parameters and their bounds for electron ptychography reconstruction optimization .... 66
Supplementary Table 5: Hyperparameters for GBDT and RF models.... 67
Supplementary Table 6: Hyperparameters settings for all optimization algorithms in benchmarks .... 67
Supplementary Table 7: Density matrices and corresponding mechanical properties of architected materials. .... 1
Supplementary Table 8: List of representative compositions of fcc CCAs predicted by DANTE .... 1
Supplementary Table 9: List of representative compositions of bcc CCAs predicted by DANTE .... 1
Supplementary Table 10: List of representative compositions of fcc CCAs predicted by MCMC.... 1
Supplementary Table 11: List of representative compositions of bcc CCAs predicted by MCMC .... 2
Supplementary Table 12: The AHC of ferromagnetic fcc Fe43.5Co18.5Ni10Al4.5Zn9.5Ir14, Fe65Ir35 as well as bcc Fe61.5Co0.5Ni0.5Si2.5Zn19Ir16 and Fe80Ir20.... 2
Supplementary Table 13: The calculated structure corresponding to each element.... 2
Supplementary Table 14: Metrics for *De novo* cyclic peptide binder design.... 3
Supplementary Table 15: Sequence for *De novo* cyclic peptide binder design.... 4
Supplementary Table 16: Percentile of high-quality solutions designed by three methods. .... 5
**Supplementary References .... 7**

# Supplementary Notes

## Brief introduction

The aim of our work is to infer the optimal solution from limited data. We present a deep active optimization pipeline that combines deep neural networks with a novel tree search to find superior solutions in complex systems with non-cumulative objectives and limited data availability. Our pipeline addresses a wide range of scenarios, covering a huge range of low- to high-dimensional problems, from easy to hard data acquisition tasks, and from simple to complex systems. Our work distinguishes itself from traditional derivative-free optimization methods, which are typically evaluated based on function evaluations rather than data acquisition. It also differs from deep-learning-based tree search models like AlphaGo, AlphaZero, and AlphaStar, which assume access to large datasets and focus on cumulative objectives. Although we cannot provide a formal statistical model due to the inherent complexity of deep learning combined with the stochastic nature of tree search (similar to AlphaGo), we provide well-reasoned motivations and extensive benchmark study and additional real-world tasks supporting our claims that our new approach DANTE (Deep Active Optimization with Neural-Surrogate-Guided Tree Exploration) notably outperforms current state-of-the-art (SOTA) methods by orders of magnitude in benchmark studies and demonstrates broad applicability across various scientific domains.

## Pseudocode

We present a high-level description of the NTE designed to find the global maximum of an objective function $x^*$ = argmax $f(x)$, $x \in X$, where $x$ is the input vector and $X \subseteq \mathbb{R}^N$ is the search space of dimension $N$. The algorithm begins by randomly sampling a dataset $D = \{(xi, yi)\}_{i=1}^{n}$, where $y_i = f(x_i)$. This dataset is then used to train a data-driven surrogate model $m$ = **Model**($D$) to fit the distribution of the dataset $D$. The pseudocode is showing below.

**Algorithm 1** Neural-surrogate-guided Tree Exploration

**Input:** function $f$, dataset $D = \{(x_i, y_i)\}_{i=1}^{n}$, exploration coefficient $c_0$, rollout round $k$.

**Output:** $X_{top}, y_{top}$

**Initialization:** surrogate model $m = \textbf{Model}(D)$, exploration weight $c = c_0 * |\max_i(m(x_i))|$

$x_{init} = x_{\operatorname{argmax}_i y_i}$, $X_{top} = \{x_{init}\}$, $y_{top} = \{\max_i y_i\}$, node visit number $N = 0$.

**for** $i = 1, 2, \cdots, k$ **do**

  create root node $v_0$ with state $x_{init}$;

  $V = \text{Expand}(v_0, \text{actions})$, y=m(V)// find leaf node of $v_0$;

  $v = \max(y + c\sqrt{\frac{2\log N}{n+1}})$;

  $N(v_0) = N(v_0) + 1, n(v) = n(v) + 1$

  **if** $\text{DUCB}_{root} > \text{DUCB}_{leaf}$:

    $x_{init} = v_0$

  **else**:

    $x_{init} = v$

**end for**

$x_{new}$ = Top n nodes

$X_{top} = X_{top} + x_{new}, y_{top} = y_{top} + m(x_{new})$

# Evaluations on synthetic functions

We evaluated DANTE alongside 11 other state-of-the-art optimization algorithms spanning key categories, including exact and heuristic algorithms, Bayesian methods, and tree-based strategies. These evaluations were conducted under two distinct experimental setups: one where node values were assigned based on exact function values and another where values were based on predictions from a surrogate model.

1. In the exact function scenario, we set the batch size for each algorithm to 1 to focus on sample efficiency. Consequently, each iteration involved a solitary expansion, search, or evolutionary step, followed by acquiring the exact function values for the offspring nodes, which served as feedback for the next iteration. The results are displayed in Supplementary Fig. 9-10.

2. In the surrogate model prediction scenario, the batch size was set to 20 for each algorithm to accommodate the time-consuming nature of model training and prediction. Here, the algorithms optimized based on the

surrogate model's predicted values, selecting 20 sample points accordingly. The ground truth for these selected points was then ascertained via the exact function, and these new data points were incorporated into the dataset for training a subsequent surrogate model. This iterative process, emblematic of self-driving virtual laboratories, advances to the next iteration with the updated model. The results are displayed in Supplementary Fig. 8.

**Setup for synthetic functions:** Besides Ackley, Rosenbrock, and Rastrigin functions introduced in Methods, we also test three other synthetic functions (Griewank, Schwefel, Michalewicz) for evaluating and analyzing the computational optimization approaches.

The Schwefel function can be written as:

$$f(x) = 418.9829d - \sum_{i=1}^{d} x_i \sin(\sqrt{|x_i|}) \tag{1}$$

Where $d$ is the dimension. The function is evaluated on the hypercube $x_i \in [-500, 500]$, for all $i$ = 1, …, $d$ with a discrete search space of a step size of 1.

The Griewank function can be written as:

$$f(x) = \sum_{i=1}^{d} \frac{x_i^2}{4000} - \prod_{i=1}^{d} \cos(\frac{x_i}{\sqrt{i}}) + 1 \tag{2}$$

Where $d$ is the dimension. The function is evaluated on the hypercube $x_i \in [-600, 600]$, for all $i$ = 1, …, $d$ with a discrete search space of a step size of 1.

The Michalewicz function can be written as:

$$f(x) = -\sum_{i=1}^{d} \sin(x_i) \sin^{2m}(\frac{ix_i^2}{\pi}) \tag{3}$$

Where $d$ is the dimension. The function is evaluated on the hypercube $x_i \in [0, \pi]$, for all $i$ = 1, …, $d$ with a discrete search space of a step size of 0.0001. The parameter settings of the synthetic functions in this study are also listed in Supplementary Table 1.

**Machine learning surrogate models:** Supplementary Fig. 3 shows the model architecture of 1D convolutional neural networks (1D-CNN) for the evaluations on synthetic functions. The model architecture and hyperparameters are determined by trial and error based on R-Squared value. Specifically, for the Ackley function,

the 1D-CNN comprises 5 convolutional layers with filter sizes of 128, 64, 32, 16, and 8 respectively, each using a kernel size of 3. It also includes 2 max-pooling layers with a pooling size of 2, 2 dropout layers with a dropout rate of 0.2, followed by a flatten layer, 2 fully connected layers with 128 and 64 units respectively, and an output layer. The loss function utilized is the mean square error (MSE). Supplementary Fig. 19 shows the model performance while evaluations on synthetic functions.

For the Rastrigin function, the 1D-CNN consists of 6 convolutional layers with filter sizes of 256, 128, 64, 32, 16, and 8 respectively. The kernel sizes are 5, 5, 3, 3, 3, and 3 respectively, with strides of 1, 2, 2, 1, 1, and 1 respectively. Following these convolutional layers is a flatten layer, 2 fully connected layers with 128 and 64 units respectively, and an output layer. The loss function used in this case is the mean absolute percentage error (MAPE).

For the Rosenbrock function, the 1D-CNN comprises 6 convolutional layers with filter sizes of 128, 64, 32, 16, 8, and 4 respectively, each using a kernel size of 3. Additionally, there are 3 max-pooling layers with a pooling size of 2, 2 dropout layers with a dropout rate of 0.2, followed by a flatten layer, 1 fully connected layer with 64 units, and an output layer. The loss function for this function is the MSE.

Moreover, the learning rate for the Adam Optimizer is set at 0.001, and the activation function utilized is the Exponential Linear Unit (ELU). The 1D-CNN model is trained for 500 epochs with an early stopping patience of 30, and a batch size of 64.

Other benchmark machine learning models, namely GBDT (Gradient Boosting Decision Tree), RF (Random Forest), and SVR (Epsilon-Support Vector Regression), are also assessed. The GBDT and RF models are implemented using the lightgbm 3.3.5 package, with the hyperparameters detailed in Supplementary Table 5. The SVR model is implemented using the scikit-learn 1.2.2 package with default hyperparameters.

**Hyperparameter settings for all algorithms in benchmarks:** We evaluated DANTE against a diverse array of state-of-the-art baseline algorithms spanning multiple categories, including Bayesian Optimization (TuRBO5 (*1*)), Evolutionary Algorithm (CMA-ES (*2*), Differential Evolution (Diff-Evo) (*3*), Shiwa (*4*)), MCTS (DOO (*5*), SOO (*5*), VOO (*6*), LAMCTS (*7*)), MCMC (*8*), Dual Annealing (DA) (*9*), and Random Search. Additionally, we assessed variations of DANTE, specifically DANTE-Greedy (which omits backpropagation) and DANTE-eGreedy (which also omits backpropagation but introduces an epsilon parameter to increase random expansions). The implementations of VOO, SOO, and DOO were sourced from an established repository (https://github.com/beomjoonkim/voot), while the methods including CMA-ES, Differential Evolution, and Dual Annealing were derived from the Scipy optimize module, and Shiwa was obtained from Nevergrad

(https://github.com/facebookresearch/nevergrad). The implementation of TuRBO5 is from https://github.com/uber-research/TuRBO. The implementation of LAMCTS is from https://github.com/facebookresearch/LaMCTS.

We further tested a BO variant that employs CNNs as surrogate models. For the implementation details of CNN-BO: We developed CNN-BO by modifying the source code of Bayesian Optimization (GitHub repository: https://github.com/bayesian-optimization/BayesianOptimization). Specifically, we replaced the original GaussianProcessRegressor with our CNNRegressor while retaining all other components of the codebase. The complete implementation of CNN-BO is publicly available at: https://github.com/Bop2000/DANTE/tree/main/CNN_BO. The results of CNN-BO are shown in Supplementary Fig. 4-7. Regarding the CNNRegressor architecture: We employ an ensemble of five CNNs with cross-validation to obtain predictive means and standard deviations. To enhance computational efficiency – as adopting the identical CNN architecture used in DANTE would incur prohibitive computational costs – we implemented a comparatively simpler 1D-CNN architecture. The 1D-CNN comprises 3 convolutional layers with filter sizes of 16, 8, and 4 respectively, each using a kernel size of 3. It also includes 1 max-pooling layer with a pooling size of 2 after the 1st convolutional layer, 1 dropout layer with a dropout rate of 0.2 after the max-pooling layer, followed by a flatten layer, 1 fully connected layer with 32 units, and an output layer. The loss function utilized is the mean square error (MSE). Moreover, the learning rate for the Adam Optimizer is set at 0.001, and the activation function utilized is the Exponential Linear Unit (ELU). The 1D-CNN model is trained for 100 epochs with a batch size of 32.

In the context of surrogate model prediction, we initiated the process with 200 randomly selected data points to train a 1D Convolutional Neural Network (1D-CNN), necessitating data preprocessing to optimize 1D-CNN performance. For the Ackley, Rastrigin, and Rosenbrock functions, the input $x$ did not do modification as it fell within the -5 to 5 range. However, the function value $f(x)$ conversion was necessary for surrogate model training to avoid gradient explosion, these conversion formulas are defined as follows:

$$F(x) = 100 / (f(x)/d + 0.01) \quad \text{for Ackely function} \tag{4}$$

$$F(x) = -f(x) \quad \text{for Rastrigin function} \tag{5}$$

$$F(x) = 100 / (f(x)/(d \times 100) + 0.01) \quad \text{for Rosenbrock function} \tag{6}$$

where $d$ represents the dimension, $F(x)$ is the convert function value. Moreover, in each iteration, the selection of the root node is based on the best function value: for Ackley and Rastrigin functions, a single data point is chosen,

whereas for other functions, top three data points are selected as starting root nodes. Further details on hyperparameter settings for all algorithms in the benchmarks are provided in Supplementary Table 6.

**Runtime:** In the exact function scenario, all methods—including DANTE, DANTE-Greedy, DANTE-eGreedy, VOO, DOO, SOO, CMA-ES, Diff-Evo, DA, Shiwa, and MCMC—are fairly fast when dimensions are below 200, this setup allows for the efficient collection of thousands of samples in a matter of minutes on an AMD R7 5800X CPU. However, in an evaluation of the 100-dimensional Rosenbrock function over $10^5$ iterations, VOO required roughly one day, DOO and SOO needed several hours, and the other methods were able to complete the process within an hour.

In the surrogate model prediction scenario, the majority of the time is devoted to training the model and utilizing it for predictions. Specifically, during an assessment of the 100-dimensional Rosenbrock function over 500 iterations (with $10^4$ samples), all methods—excluding TuRBO5 and LAMCTS—concluded the task within a day when employing a 3090ti GPU for model training and predictions, and a CPU for other computations. In contrast, TuRBO5 and LAMCTS required several days to accumulate thousands of samples on a CPU, as these methods generate only one sample per iteration.

**Ablations on hyperparameters: Exploration weight ratio ($c_0$)** This parameter controls the weight of exploration during tree search. A large $c_0$ prompts DANTE to more frequently explore uncharted regions (exploration). As shown in Supplementary Fig. 20, too small $c_0$ results in poor performance on the Rosenbrock function, underscoring the importance of exploration. Conversely, a large $c_0$ leads to over-exploration which is likewise undesirable. We recommend setting $c_0$ to 0.01 to 1. **Expansion actions** This parameter dictates how the tree's nodes expand. Supplementary Fig. 21 illustrates that relying solely on deterministic moves (e.g., ± 0.1 for the Ackley function) for node expansion results in poor optimization performance since it is hard to jump out from the local minimum. Similarly, stochastic moves alone also led to relatively bad optimization results., Combining deterministic and stochastic moves enhances optimal performance across all functions, establishing this approach as the default setting. Further evaluations are presented in Supplementary Fig. 22-23.

**Benchmark performance at low dimension and low initial data:** We evaluated DANTE, BO, TurBO5, and BO-CNN (i.e., CNN-BO or DNN-BO) on synthetic functions at low dimension and low initial data scenario. Test functions are Ackley-10d, 20d; Rastrigin-10d, 20d; Rosenbrock-10, 20d; Griewank-10d, 20d; Schwefel-10d, 20d. As the results shown in Supplementary Fig. 4-5, it can be observed that the BO-based method can often have a faster convergence rate at the beginning at a small initial dataset (<=20) and low dimension (=10). But eventually, as data accumulates, DANTE is able to find a better optima. Notably, as dimensionality increases, DANTE

demonstrates clearer advantages. As shown in Supplementary Fig. 6, DANTE demonstrates a clear advantage over the rest of algorithms while initial dataset (=200) is sufficient for training a high precision CNN model.

**Effects of different sampling batch size:** We evaluated DANTE on synthetic functions with different sampling batch sizes (n = 5, 10, 20). Test functions are Ackley-20d, 100d; Rastrigin-20d, 100d; Rosenbrock-20, 100d; Griewank-20d; Schwefel-20d. As the results shown in Supplementary Fig. 7, smaller sampling batch sizes lead to a faster convergence rate.

**Optimization efficiency versus dimension:** To better understand the relationship between 1) optimization efficiency and problem dimension, and 2) optimization efficiency and the degrees of freedom (DoF) per dimension, we conducted two additional tasks using benchmark test functions. These analyses aimed to explore how these factors influence the optimization process in more detail. Supplementary Fig. 14 demonstrates the influence of changes in the Degree of Freedom of the descriptor to the convergence behavior (Ackley-20d, Ackley-100d, Rastrigin-20d, Rastirigin-100d, Rosenbrock-20d, Rosenbrock-100d). As observed, as the degrees of freedom (DoF) increase, DANTE remains capable of achieving convergence, although it requires a larger amount of data. A heuristic relationship can be summarized as follows:

$$\text{Number of data points needed to reach the optimum} \approx c * \log(d * \text{DoF})$$

where $c$ is a constant and $d$ represents the dimension. However, this formula does not explicitly account for the complexity of the nonlinear landscape itself and should therefore be used with caution.

Supplementary Fig. 24 demonstrates the influence of change of dimension to the optimization efficiency on Ackley and Rastrigin functions. It can be observed that the data needed to achieve optimum scales linearly with the dimensionality.

# Real world tasks with easy data acquisition

## 1. Neural network architecture search

Neural architecture search (NAS) is an automated method for discovering optimal neural network architectures by systematically exploring and evaluating various network configurations to achieve the best performance on a given task. We evaluated DANTE compared to six other optimization algorithms: Random Search, MCMC, CMA-ES, Dual Annealing, LA-MCTS, and TuRBO5.

**Dataset and optimization Target:** To benchmark the efficacy of DANTE in optimizing neural network structures within the context of active learning, we choose the NAS-Bench-101 dataset (*10*), which contains over 400,000 unique convolutional neural networks along with their corresponding performance metrics, trained on the CIFAR-10 dataset (*11*). Each neural network is represented by a 7×7 upper-triangular adjacency matrix with up to 9 edges, where nodes represent specific operations and edges denote the connection relationships between these operations. The first operation represents the input, and the last represents the output, while the remaining five components can be selected from 3×3 convolution, 1×1 convolution, or 3×3 max-pooling. The objective of the NAS task is to identify an optimized neural network structure that achieves the highest classification accuracy on the test set (test acc).

**Neural network architecture encoding:** We adopt a truncated 40-bit path-based encoding scheme (*12*) to represent the neural network structure, where each bit corresponds to a specific path from the input layer to the output layer, incorporating various operators along the way. For optimization algorithms like CMA-ES, Dual Annealing, LA-MCTS, and TuRBO5, which require a well-defined search domain, we parameterize the neural network structure into a 36-dimensional vector within the continuous [0, 1] space, as adopted from prior work (*13*). The first 21 entries correspond to the adjacency matrix, where the largest values set the respective elements in the matrix to 1. The remaining 15 entries represent the one-hot encoding of 5 components, each with three possible operations. For DANTE, MCMC, and Random Search, optimization is performed directly at the adjacency matrix level.

**Surrogate model:** We train a 1D-CNN model to map the path encoding into the test acc. The 1D-CNN consists of 5 convolutional layers with filter sizes of 128, 64, 32, 16, and 8, respectively, each using a kernel size of 3. It also includes 2 max-pooling layers with a pooling size of 2, 2 dropout layers with a dropout rate of 0.2, followed by a flatten layer, 2 fully connected layers with 128 and 64 units, respectively, and a final output layer. The loss function used is mean square error (MSE).

**Active learning loop:** The optimization process begins by generating 200 random initial data points from NAS-Bench-101, which are used to train the initial surrogate model. In the active learning loop, optimization algorithms then sample 20 optimized successors by refining the surrogate model, expanding the dataset. The updated surrogate model is subsequently used in the next iteration of the loop, continually improving the optimization process.

**DANTE settings:** The exploration weight ratio is set to 0.1, with 100 rollout rounds. These rollouts include stochastic moves in the adjacency matrix, stochastic moves in operations, and stochastic moves in both the

adjacency matrix and operations, each with equal probability. The ratio between max, visit, and random sampling is 5:1:1.

**MCMC settings:** The acceptance rate is defined as exp(-δ/T), where δ represents the difference between the proposal point and the current best point. If δ > 0, indicating the proposal point is better than the current best, the proposal is accepted outright; otherwise, it is accepted with the calculated acceptance rate. The temperature parameter, T, decreases exponentially with each iteration, starting at an initial value of 0.01, with a half-life of 200 iterations.

**CMA-ES settings:** 0.25 sigma0, 300 maxfevals, with other parameters using default settings.

**Dual Annealing setting:** 5 maxiter, 300 maxfun, with other parameters using default settings.

**La-MCTS settings:** 40 ninits, 0.1 Cp, 100 iterations, with other parameters using default settings.

**TuRBO5 settings:** 50 n_init, 300 max_evals, 5 n_trust_regionsm, 10 batch_size, 2000 max_cholesky_size, 50 n_training_steps, with other parameters using default settings.

## 2. Soft magnetic alloy design

Soft magnets are extensively applied in electrical power generation and transformation, as well as sensors. Beyond the traditional soft magnets like Permalloy, high-entropy alloys (HEAs) offer a new plethora for the design of soft magnets with potentially multi-functionality. The soft magnets usually require high magnetization saturation, high electrical resistivity for reduced energy loss in application, and low coercivity. Owing to the complexity of addressing the coercivity more of an extrinsic property, in this work we focus on maximizing simultaneously the magnetization saturation and electrical resistivity. We evaluated DANTE compared to two other optimization algorithms: Random Search and MCMC, since they can efficiently conform to the multi-constraints of alloy design tasks.

**Feature engineering:** We adopt 27 elements: Fe, Co, Ni, Ta, Al, Ti, Nb, Ge, Au, Pd, Zn, Ga, Mo, Cu, Pt, Sn, Cr, Mn, Mg, Si, Ru, Rh, Hf, W, Re, Ir, and Bi, to design 6-element CCAs with fcc structure. For Fe, Co, and Ni, the atomic ratio ranges from 0 to 100 at.%, while for other elements, it ranges from 0 to 40 at.%, with 0.5 at.% interval. Additionally, the total atomic percentage of Fe, Co, and Ni is designed to fall between 60 at.% to 80 at.%. For CCAs with a fcc crystal structure, the Fe / (Co + Ni) ratio is required to be less than or equal to 1.5.

**Optimization Target:** The optimization target is to maximize the following target:

$$Target = M * rho \tag{1.1}$$

$M$ stands for magnetic moment and $rho$ resistivity.

**Surrogate model:** The 1D-CNN comprises 4 convolutional layers with filter sizes of 64, 32, 16, and 8 respectively, each using a kernel size of 3. The convolutional layers with 64 and 32 layers are using a stride of 2. One batch normalization layer and a dropout layer are employed to prevent overfitting. Before the output layer, there is a flatten layer and a fully connected layer with 128 units. The loss function utilized is MSE. Moreover, the learning rate for the Adam Optimizer is set at 0.001, and the activation function is ELU. The 1D-CNN model is trained for 5000 epochs with an early stopping patience of 100, and a batch size of 50.

**DANTE settings:** The exploration weight ratio $c_0$ is set to 0.05. Each iteration selects 4 data points that exhibit the best properties, alongside one randomly selected data point, to serve as initial root nodes for the rollout process. For each root node, DANTE conducts 200 rollouts, selecting 30 samples for further consideration. Of these, 15 are chosen for their highest predicted $M * rho$ values, 10 for their high feature rankings (ranked by their predicted scores and Euclidean distance from the original data points), 3 for being the most frequently visited, and 2 randomly. The rollouts include three equally probable types of expansion actions: element variation, content variation, and a blend of both. Node values are determined by averaging the predictions from five surrogate models. 20 samples are finally selected in each iteration for DFT calculations, chosen based on their feature rankings and predicted scores.

**MCMC settings:** MCMC selects the node with highest $M * rho$ value as initial root node, and conducts 1000 rollouts. The expansion actions are same as DANTE. 20 samples are finally selected in each iteration, chosen based on their feature rankings and predicted scores.

**Random Search settings:** For each iteration, 100,000 data points are randomly generated and evaluated by the surrogate model. Then, 20 samples are finally selected based on their feature rankings and predicted scores.

## 3. Lunar landing control problem

The Lunar Lander problem is a well-established benchmark environment in the OpenAI Gym toolkit, commonly used in the field of reinforcement learning (RL) to evaluate control strategies. The objective is to control a simulated lunar module to ensure a safe landing on the moon's surface, specifically within a designated target zone located between two flags. The task presents multiple challenges, including the need to optimize the use of fuel, manage thrust efficiently, and maintain stability to avoid crashes, all while adhering to the system's dynamic constraints. This problem serves as a test bed for evaluating the performance of RL algorithms in control tasks.

The **state space** is an 8-dimensional vector representing: X and Y positions of the lander; X and Y velocities; Angle of the lander; Angular velocity; Boolean values indicating if the left or right legs have made contact with the ground. The **actions** are discrete and include: Do nothing; Fire left engine; Fire main engine; Fire right engine. The agent gets **rewards** based on how close the lander is to the target landing zone: Positive reward for landing close to the target; Negative reward for crashing; Penalty for using fuel (firing engines); Rewards for coming to rest.

This problem is typically framed as a trajectory planning task with cumulative objectives; however, by fixing the initial conditions, we **reformulate it as a non-cumulative problem, where the goal is to design an optimal sequence of actions to maximize the reward.** We evaluated DANTE compared to 9 other optimization algorithms: Random Search, DOO, SOO, VOO, Shiwa, CMA-ES, Differential Evolution, Dual Annealing, and MCMC.

**Feature engineering:** We adopt a 100-dimension sequence as the input actions, each action is discrete and includes: 0 denotes do nothing, 1 denotes fire left engine, 2 denotes fire main engine, 3 denotes fire right engine. The seed of environment reset is set to 42 to get a fixed initial state of the lunar landing problem.

**Optimization Target:** The goal is to design an optimal sequence of actions to maximize the reward.

**DANTE settings:** The exploration weight ratio $c_0$ is set to 0.05. The rollouts include 6 equally probable types of expansion actions: stochastic moves of 1, 2, 5, 10, 20, 33 elements of the 100d sequence.

**Random Search settings:** Random seed is set to 42.

**DOO settings:** 0.1 explr_p with other parameters using default settings.

**SOO settings:** Default settings.

**VOO settings:** 1 explr_p with other parameters using default settings.

**Shiwa settings:** Default settings.

**CMA-ES settings:** Default settings.

**Differential Evolution settings:** Default settings.

**Dual Annealing settings:** Default settings.

**MCMC settings:** Default settings.

## 4. Electron ptychography

Electron ptychography, a phase-contrast imaging technique, has emerged as a promising solution to overcome these challenges and achieve sub-angstrom resolution and three-dimensional depth sectioning in samples thicker than 20 nm (*14*). The goal of ptychographic reconstruction is to quantify the phase of the transmission function within the atomic lattice. The quality of this reconstruction relies on a careful selection of various reconstruction parameters, including physical, optimization, and experimental parameters, which collectively affect the quality and accuracy of the retrieved transmission function. The parameter space can be vast and complex, and the optimal choice depends on the specific dataset and measurement conditions. Currently, the parameter selection is mainly based on expert knowledge and trial-and-error, which limits the efficiency and applicability of electron ptychography. Therefore, it is advantageous to employ an automated pipeline for the experimental parameter calibration with minimal human effort. Specifically, this optimization task involves finding the optimal reconstruction parameters to retrieve the underlying transmission function. This task is equivalent to solving a non-convex problem in an 8-dimensional parameter space. The objective is to iteratively minimize the normalized mean square error (NMSE) between the measured and modeled diffraction patterns (Supplementary Fig. 15).

Scanning transmission electron microscopes (STEM) with aberration correctors have been capable of characterizing nanostructures at a sub-angstrom resolution (*15*), it is a powerful tool to discover and design new materials. However, atomic-resolution imaging of nanostructures by STEM is often hindered by multiple electron scattering in samples thicker than a monolayer (*16*), as well as by beam-induced damage in sensitive materials (*17*).

**Feature engineering:** The feature vector consists of 8 variables: beam energy, defocus, maximum number of iterations, number of iterations with identical slices, probe-forming semi-angle, update step size, slice thickness and number of slices. Detailed values and their bounds are listed in Supplementary Table 4.

**Optimization Target:** The objective function NMSE is calculated between the positive square-root of the measured diffraction pattern $I_M$ and the modulus of the Fourier-transformed simulated exit-wave $\Psi$, which can be formulated as:

$$\frac{1}{N}\sum_{i}^{N}\left|\sqrt{I_{M(i)}(\mathbf{u})}-\left|F\left[\Psi_{i}(\mathbf{r})\right]\right|\right|^{2} \qquad (1.2)$$

where **r** and **u** denote the real- and reciprocal-space coordinate vectors, respectively, and $N$ is the total number of the measured diffraction patterns.

**Correlation index:** The degree of matching for a given template $T$ by intensity function $P$ is characterized by a correlation index, which can be defined by the following relation:

$$\frac{\sum_{i=1}^{m} P(x_i, y_i) T(x_i, y_i)}{\sqrt{\sum_{i=1}^{m} P^2(x_i, y_i)} \sqrt{\sum_{i=1}^{m} T^2(x_i, y_i)}} \tag{1.3}$$

where $(x_i, y_i)$ is the coordinate of pixel $i$.

**Dataset simulation:** abTEM (*18*), an open-source package, is used for the simulation of a transmission electron microscopy experiment. For this case study, we simulated a 4D dataset of 18-nm-thick silicon along the [110] direction with Poisson noise.

**Ptychographic reconstruction:** The analysis is performed using py4DSTEM (*19*), a versatile open-source package for different modes of STEM data analysis. See Supplementary Fig. 11 and 15 for more details about the reconstruction process.

**DANTE settings:** The exploration weight ratio $c_0$ is set to 0.05. We use 20 samples for the initialization and rollout for 2,500 iterations. The value of each node is ascertained by ground truth (NMSE). These rollouts include two categories of expansion actions: stochastic moves, with a 2/3 probability, and deterministic moves, comprising the remaining 1/3. Within the stochastic category, three potential variations are considered: altering a single variable in $x$, modifying $d$/3 variables in $x$, and adjusting $d$/2 variables in $x$. where $d$ is 8 in this case, $x$ is the parameter matrix.

**TuRBO5 settings:** 20 initial samples, 5 independent trust regions, with other parameters use default settings in the reference implementation.

**Bayesian Optimization settings:** 20 initial samples, other parameters use the default setting in package bayes_opt.

## Real world task 1: Architected materials design

In the following sections, we address real-world problems that involve astronomically large search spaces, numerous external constraints, noisy labels, and highly nonlinear input-output relationships. In such cases, the

labels may contain various forms of noise, and acquiring them is both resource-intensive and time-consuming, with the optimum often remaining elusive. We demonstrate that the DANTE framework can effectively address these complex tasks without relying on large datasets. Compared to existing methods, the DANTE pipeline identifies superior solutions, laying the groundwork for discovering new insights with fewer data points.

We begin with architected materials, a class of materials defined by tailored behaviors arising from the interaction between their material properties and geometry, making them one of the most widely utilized material types (*20*). The objective is to identify the geometric configuration that yields the best yield strength with constant Young's modulus using minimal data, as validation relies on finite element analysis, which is computationally expensive and can introduce noise due to numerical errors (*21*, *22*). For instance, Peng et al. have shown that the numerical errors of FEM implemented in this study is about 10% (*23*). Additionally, the vast number of possible geometries and the highly nonlinear relationship between properties and geometry make DANTE particularly well-suited for this task.

Recently, Peng et al. developed a multi-objective AL pipeline ('GAD') for orthopedic applications (*23*). This pipeline combines a deep-learning-based generative model and a high-fidelity simulator to iteratively optimize the architecture to achieve the desired elastic modulus (E) and yield strength (Y). GAD serves as an ideal benchmark for DANTE (detailed settings can be found in following texts and Supplementary Fig. 17 and 25). Here, we adopt the 3*3*3 cubic arrangement of the Gyroid units as the model input and start the AD task with the same initial labeled dataset adopted by GAD (see Methods for details).

Extended Data Fig. 1a compares the performance of DANTE and GAD. DANTE identifies superior designs within five rounds, achieving a yield strength (Y) of 82.1 MPa, whereas the best design from GAD only reaches 75.0 MPa, representing a 10% shortfall compared to DANTE. The U-MAP representation in Extended Data Fig. 1b illustrates that GAD tends to focus on a limited number of clusters within the design space, while DANTE explores a wider region, resulting in notable strength improvements of 41% compared to uniform designs.

The simulated strain-stress curves of the scaffolds shown in Extended Data Fig. 1c confirm that the DANTE designs achieve yield strengths that higher than those of the uniformly designed scaffold and the previous GAD scaffold, respectively (for details on Von Mises stress distribution and hydrostatic pressure analysis, see Supplementary Fig. 26-27 and Supplementary Table 7). Notably, GAD relies on a knowledge-based, large unlabeled dataset to train its generative model for creating new structures, which may introduce inductive bias. In contrast, DANTE is easier to implement and unbiased, requiring only a labeled dataset for surrogate model training.

**DANTE settings:** The exploration weight ratio $c_0$ is set to 0.02. In each iteration, 8 data points characterized by suitable elastic modulus and the highest yield strength, in addition to two randomly selected data points, are chosen as starting root nodes for the individual rollout process. For every root node, DANTE performs 100 rollouts, from which 7 samples are selected: 5 exhibiting the highest predicted yield strength, one most frequently visited, and one selected at random. These rollouts encompass two types of expansion actions: stochastic moves, with a 2/3 probability, and deterministic moves (± 0.1), comprising the remaining 1/3. Within the stochastic category, three potential variations are considered: altering a single variable in $x$, modifying $d/5$ variables in $x$, and adjusting $d/10$ variables in $x$. where $d$ is 27 in this case, $x$ is the density matrix. Node values are derived by averaging the predictions from five surrogate models. The t-SNE technique is utilized to project both sampling and original data points into a 2-dimensional space, subsequently organizing the sampling points according to their Euclidean distance from the original data points in this space for feature ranking. Each iteration culminates in the selection of 20 samples for FE simulation; 15 of these are chosen based on their feature and predicted yield strength rankings, while 5 are selected randomly. Further information is available on GitHub.

**GAD-MALL settings:** The settings can be found in (*23*)

**Surrogate model:** Supplementary Fig. 17 shows the model architecture of 3D convolutional neural networks (3D-CNN) for the prediction of the architected material's yield strength and elastic modulus. The 3D-CNN comprises 3 convolutional layers with filter sizes of 8, 4, and 2 respectively, each using a kernel size of 3. Each convolutional layer is followed by a max-pooling layer with a pooling size of 2. Before the output layer, there is a flatten layer and 3 fully connected layers with 128, 64, and 32 units respectively. The loss function utilized is MSE. Moreover, the learning rate for the Adam Optimizer is set at 0.001, and the activation function utilized is ELU. The 3D-CNN model is trained for 5000 epochs with an early stopping patience of 100, and a batch size of 32. Supplementary Fig. 25 shows the model performance of the self-driving virtual laboratory.

## Real world task 2: Compositionally complex alloy design

In this section, we apply DANTE to optimize the composition of Compositionally Complex Alloys (CCAs) to enhance their transport properties. CCAs, an emerging class of materials with five or more principal elements (≥ 5 at.%), have garnered increasing attention for their multifunctional potential, particularly in mechanical, magnetic, and catalytic applications (*24*). However, several challenges arise: 1) The combinatorial complexity is immense, with approximately 4.6 million possible combinations for five-component CCAs using a 1 at.% interval, and even more when considering a broader range of elements (27 in this study). 2) Density Functional Theory (DFT) calculations for transport properties are computationally expensive and subject to noise due to numerical

errors (*25*). 3) Certain compositional constraints must be imposed to prevent computational divergence in DFT calculations (e.g., the Fe/(Co + Ni) ratio is constrained to be ≥ 1.5; for more details, see Methods). These factors necessitate a method capable of optimizing with minimal data, making DANTE particularly well-suited to this task (*26*, *27*).

Here, we investigate an intriguing yet underexplored electronic property—large anomalous Hall conductivity (AHC, $\theta_{xy}$) and anomalous Hall angle (AHA, $\sigma_{xy}/\theta_{xx}$)—which have been reported in half-Heusler systems such as GdPtBi (*28*) and TbPtBi (*29*), showing great promise for advanced electronic and spintronic applications. Given that chemical disorder is commonly expected and adjustable in ferromagnetic CCAs (*28*), we are motivated to explore chemically disordered CCAs that may exhibit enhanced AHA and AHC.

The validation of DANTE-designed compositions involves density functional theory (DFT) calculations of anomalous Hall conductivity (AHC) and anomalous Hall angle (AHA) using the linear response Kubo-Bastin formalism (see Methods). Our objective is to maximize the product of AHC and AHA while adhering to a formation energy constraint of 0.02 ± 0.002 Ry/site. This constraint accounts for the configurational entropy of complex concentrated alloys (CCAs), which can mitigate positive formation energies (see Methods). DANTE incorporates these constraints into the leaf node generation process, discarding any leaf nodes that do not satisfy the specified criteria. We focus on the prevalent body-centered cubic (bcc) structure in CCAs (*30*) and apply distinct Fe/(Co+Ni) ratio ranges to the phase to further refine the predicted compositions (see Methods).

Extended Data Fig. 1d presents a comparison of results obtained using DANTE and Markov Chain Monte Carlo (MCMC), demonstrating that DANTE achieves higher anomalous Hall angle (AHA) and anomalous Hall conductivity (AHC), with a 28.5% improvement over MCMC (Supplementary Figures 28-31). Extended Data Fig. 1e illustrates the latent distribution through a 2D U-MAP representation (refer to Supplementary Tables 9-12 for the compositions of CCAs predicted by DANTE and MCMC). DANTE explores a composition space distinct from the initial distribution, identifying an optimal composition that is notably different from that found by MCMC. This suggests that MCMC may be trapped in a local optimum.

To further investigate the mechanism underlying the enhanced electronic transport properties of the best alloy discovered by DANTE, we analyze their electronic structures by examining the degree of smearing, defined as the curve fitting in the minority-spin channel along a selected momentum path (*31*). Extended Data Fig. 1f illustrates the degree of smearing for the optimal composition at each iteration (see Supplementary Figures 32–35). The results show that the degree of smearing increases with each iteration, indicating a stronger disorder scattering effect.

This enhancement can be attributed to the inclusion of Zn in DANTE's CCAs, as its *d* orbitals exhibit full band filling ($3d^{10}$) with the *d*-band center located at a lower energy level (~ -0.5 Ry relative to Fermi energy) compared to Pt, which is preferred by MCMC. As a result, the band center mismatch among different elements is larger (*32*). DANTE effectively identifies this distinction between Zn and Pt, uncovering a superior combination of elements (see Supplementary Figures 35–37).

**DANTE settings:** The exploration weight ratio $c_0$ is set to 0.02. Each iteration selects 8 data points that exhibit the best properties, alongside two randomly selected data points, to serve as initial root nodes for the rollout process. For each root node, DANTE conducts 100 rollouts, selecting seven samples for further consideration. Of these, five are chosen for their highest predicted AHA*AHC values, three for their high feature rankings (ranked by their predicted scores and Euclidean distance from the original data points), one for being the most frequently visited, and another randomly. The rollouts include three equally probable types of expansion actions: element variation, content variation, and a blend of both. Node values are determined by averaging the predictions from five surrogate models. 20 samples are finally selected in each iteration for DFT calculations, chosen based on their feature rankings and predicted scores. More details can be found at GitHub.

**MCMC settings:** Similar to DANTE, yet without setting the exploration weight ratio $c_0$.

**Surrogate model:** Supplementary Fig. 18 shows the model architecture of 1D-CNN for the prediction of CCA's formation energy, AHA, and AHC. The 1D-CNN comprises 4 convolutional layers with filter sizes of 64, 32, 16, and 8 respectively, each using a kernel size of 3 and a stride of 2. One batch normalization layer and a dropout layer are employed to prevent overfitting. Before the output layer, there is a flatten layer and a fully connected layer with 128 units. The loss function utilized is MSE. Moreover, the learning rate for the Adam Optimizer is set at 0.001, and the activation function is ELU. The 1D-CNN model is trained for 5000 epochs with an early stopping patience of 1000, and a batch size of 50. Supplementary Fig. 28-31 shows the model performance of the self-driving virtual laboratory.

**Density functional calculation:** The calculated anomalous Hall resistivity (AHR) for fcc $Al_{0.25}CrFeCoNi$ and bcc $Al_{1.25}CrFeCoNi$ are 0.879 and 1.699 μΩ cm, respectively, which are in reasonably good agreement with experimentally measured AHRs of 0.5 and 1.5 μΩ cm (*33*). Moreover, as reported in Ref. (*34*), for disordered $Fe_{50}Pd_{50}$ and $Ni_{50}Pd_{50}$ alloys, the calculated $\sigma_{xy}$ with vertex corrections are 0.541 and -1.400 $(\text{m}\Omega\,\text{cm})^{-1}$. In comparison, the experimental values are 0.303 and -1.293 $(\text{m}\Omega\,\text{cm})^{-1}$, respectively.

In the calculation of the conductivity tensor $\sigma_{\mu\nu}$, a fully relativistic Dirac four-component scheme for the basis functions was used throughout with an angular momentum cutoff of $l_{max} = 3$. The self-consistent field (SCF) potential was obtained by employing the Vosko-Wilk-Nussair (VWN) parametrization (*35*) for the exchange-correlation functional in the local density approximation (LDA). The energy integration was performed on a semicircle on the complex energy plane using 64 energy points and $36^3$ $k$-points in the Brillouin zone (BZ) for bcc and fcc CCAs. The atomic sphere approximation (ASA) was employed as a shape approximation for the potential. Using the optimized SCF potentials, subsequent KB conductivity tensor calculations were performed using $10^6$ $k$-points to ensure convergence. The lattice parameters of CCAs were calculated by averaging the experimental volume of each constituent element over atomic concentration, as validated in Ref. (*36*).

Besides the transport properties, we also evaluated the formation energy of the CCAs with respect to the (meta-)stable single elements using the Exact Muffin-tin Orbitals (EMTO) package (*37*). The reason for the selection of meta-stable crystal structure for some single elements as reference is owing to the relatively lower accuracy of the KKR-based approach in handling low-symmetry structures (see Supplementary Table 13).

**Smearing of Bloch spectral function:** The degree of smearing is quantified following the work of Szotek et al. (*38*) and Robarts et al. (*31*), where the momentum spectral function is considered as Lorentzian,

$$\rho(\boldsymbol{k},\epsilon) = \frac{1}{\pi}\left(\frac{\hbar}{\tau_{\boldsymbol{k}}}\right)\left[\frac{1}{(\epsilon-\epsilon_{\boldsymbol{k}})^2+(\hbar/\tau_{\boldsymbol{k}})^2}\right] \quad (7)$$

where $\epsilon_{\boldsymbol{k}}$ is the location in energy and $\tau_{\boldsymbol{k}}$ the lifetime of a band near Fermi energy. By integrating $\rho(\boldsymbol{k},\epsilon)$ over energy, we obtain,

$$n(\boldsymbol{k}) = \int_{-\infty}^{E_F} \rho(\boldsymbol{k},\epsilon)d\epsilon = \frac{1}{2} + \frac{1}{\pi}\arctan\left[\frac{\epsilon_F-\epsilon_{\boldsymbol{k}}}{(\hbar/\tau_{\boldsymbol{k}})}\right] \quad (8)$$

In practice, as implemented in Ref. (*31*), the $n(\boldsymbol{k})$ curves were fitted with tanh functions via two parameters $\boldsymbol{k}_F$ and $b$,

$$n(\boldsymbol{k}) = \frac{1}{2}\left[1 - \tanh\left(\frac{k-k_i}{b_i}\right)\right] \quad (9)$$

In other words, $n(\boldsymbol{k})$ was fitted by a sum of tanh functions centered at $k_F$ with broadening width $b$. Based on this equation, the curve fitting was performed as follows,

$$n(\boldsymbol{k})_{max} - n(\boldsymbol{k})_{min} = \sum_{i=1}^{N}\left(\frac{1}{2}\left[1 - \tanh\left(\frac{k-k_i}{b_i}\right)\right]\right)/N * (n(\boldsymbol{k})_{max} - n(\boldsymbol{k})_{min}) \quad (10)$$

i.e., the variation of $n(\boldsymbol{k})$ along the selected k-path is normalized. In fcc system, the selected k-path was along [110] direction and the value of *N* was selected to be three; in bcc system, the selected k-path was along [001] direction and $N = 2$. The smearing corresponding to each material was then obtained by averaging over $b_i$, i.e., $\sum_{i=1}^{N} b_i / N$.

**Supplementary results:** We list in Supplementary Tables 8-11 some representative compositions of fcc and bcc CCAs predicted by DANTE and MCMC algorithms, respectively. In general, the CCAs provided by DANTE

demonstrate superior combinatorial properties in terms of AHC and AHA. It is apparent that the predicted compositions by MCMC, especially for the bcc CCAs, are way more dispersed. Moreover, we find that the bcc and fcc CCAs optimized by DANTE consist of similar elements, that is, FeCoNiZnIrAl(Si). Driven by this observation, we further investigate both the bcc and fcc binary $Fe_{1-x}Ir_x$ systems. The calculated formation energies suggest that when $x > 0.3$, fcc phase is more thermodynamically stable than bcc phase. As demonstrated in Fig. 2, the bcc $Fe_{0.7}Ir_{0.3}$ exhibits the largest AHA of about 0.062, which is however smaller than the predicted bcc CCAs (~0.085). Similarly, in binary FeIr alloys with the fcc structure, the AHA is maximally 0.032, whereas the maximal AHA of fcc CCAs is approximately 0.070.

Furthermore, the electronic structure, which can most easily be described through the k-resolved density of states known as the Bloch spectral function (BSF, $A_B(E, k)$), is investigated to elucidate the distinctions between binary alloys and CCAs. The fcc $Fe_{43.5}Co_{18.5}Ni_{10}Al_{4.5}Zn_{9.5}Ir_{14}$ and $Fe_{65}Ir_{35}$, as well as bcc $Fe_{61.5}Co_{0.5}Ni_{0.5}Si_{2.5}Zn_{19}Ir_{16}$ and $Fe_{80}Ir_{20}$ are chosen as representative examples for comparative analysis (see Supplementary Fig. 33-34). It can be obviously seen that the BSFs are more strongly smeared out in CCAs than in binary FeIr alloys, especially in the majority spin channel. The smearing indicates strong electron scattering due to atomic disorder and can be used to estimate the quasiparticle coherent length and further the electrical conductivity (*31*). The stronger smearing in CCAs leads to lower electrical conductivity and hence larger AHA.

Another intriguing aspect introduced by chemical disorder is the vertex correction that captures extrinsic (side jump and skew scattering) contributions to the transport tensors. As listed in Supplementary Table 12, vertex correction plays a dominant role particularly in the off-site Fermi surface term $\sigma_{xy}^{1,I}$. Even more notably, the degree to which the induced increase of $\sigma_{xy}^{1,I}$ in CCAs by the vertex correction exceeds that in binary alloys is more than double. Furthermore, by comparing the AHC and AHA of the predicted CCAs in this work, i.e., $|\sigma_{xy}| > 0.900\ (m\Omega\ cm)^{-1}$ and $|\sigma_{xy}|/\sigma_{xx}$ up to 0.093, with other AHE alloys, e.g., $L1_0$ FePt thin film with $|\sigma_{xy}| = 1.243$ and $|\sigma_{xy}|/\sigma_{xx} = 0.03$, we conclude the substantial potential of utilizing DANTE for the efficient design of CCAs with multiple target properties.

## Real world task 3: *De novo* cyclic peptide binder design

In this section, we aim to design protein sequences from scratch to identify proteins with high binding scores using the DANTE pipeline. Cyclic peptides are a class of cyclic compounds that have shown considerable promise as antibiotics and therapeutics in recent years due to their stability, high specificity, and excellent membrane permeability (*39*). However, even for a simple 16-residue sequence, there are $16^{20}$ possible combinations, and the

relationship between sequence and properties can be highly complex. Consequently, the computational design of cyclic peptide binders targeting specific proteins remains a notable challenge (*40*). By employing the DANTE approach for optimizing cyclic peptides, we can achieve a more rapid discovery of superior peptide sequences compared to other AL methods.

In this study, the amino acids (AAs) in the cyclic peptide are interconnected by amide or other chemically stable bonds between the C– and N–termini or between the head and tail, creating a high-dimensional and complex search space (*41*). We integrate DANTE with AlphaFold2 and Rosetta (protein design software) as validation tools to design cyclic peptides from scratch, specifically targeting the optimization of protein-protein interactions (PPIs). The objective is to maximize the product of two Rosetta binding metrics, which are widely used for characterizing the strength of PPIs: shape complementarity (SC) and the change in Solvent Accessible Surface Area (dSASA) (*42*) (see Methods). The optimal design is anticipated to exhibit high values for both SC and dSASA.

DANTE starts with an input that includes a hotspot region from the target protein, a random AA sequence, and a positional encoding that indicates connectivity. AlphaFold2 processes this input to predict the target-peptide complex, from which shape complementarity (SC) and the change in Solvent Accessible Surface Area (dSASA) are calculated using Rosetta software (*43*). These metrics are then fed back into the DANTE framework. DANTE iteratively optimizes these metrics by adjusting the AA sequence in search of binders with higher target scores. In this approach, the metrics computed by Rosetta are directly utilized as node values.

DANTE was applied to a cyclic peptide-protein dataset, with results compared to those obtained using gradient descent (GD) and Markov Chain Monte Carlo (MCMC) methods (see Supplementary Table 14) (*44*). As shown in Extended Data Fig. 1g, starting from a random sequence, the binding metrics of the DANTE designs progressively improve, achieving statistically notable enhancements over the GD, MCMC designs, and native peptides. (Mann-Whitney test $P_{\mathrm{GD}}$ = 1e-8, $P_{\mathrm{MCMC}}$ =2.1e-6, $P_{\mathrm{native}}$ = 0.048, alternative = 'greater'). The designed sequences are detailed in Supplementary Table 15.

It is noteworthy that the designed cyclic peptides adopt binding conformations similar to those of the native peptides, occupying the same active site despite possessing notably different sequences. To elucidate the molecular basis for the differences between the DANTE designs and the native binder (see Supplementary Tables 14 and 15), we conduct an interaction map analysis of a specific example, focusing on casein kinase 2 (PDB ID: 4IB5) and the cyclic peptide designed by DANTE (hereafter referred to as the DANTE peptide).

The DANTE peptide exhibits enhanced interactions compared to the native peptide (DANTE: SC = 0.79, dSASA = 1228 Å$^2$ vs. Native: SC = 0.63, dSASA = 1046 Å$^2$). This improvement can be attributed to DANTE's incorporation of larger hydrophobic groups near the interface and an increase in hydrophilic contacts (as illustrated in Supplementary Fig. 38). The AlphaFold2-predicted complex, shown in Extended Data Fig. 1h, reveals a hydrogen-bonding network. In addition to the interactions between the peptide and residues Q34, E50, and L39—similar to those found with the native binder—the imidazole groups of residues H1 and H11 form additional salt bridges with the acidic side chains of D101 and E50, respectively. This engagement facilitates the anchoring of the peptide to the protein surface, thereby stabilizing the peptide-protein complex.

Additionally, the interaction diagram presented in Extended Data Fig. 1i indicates that the DANTE peptide strategically incorporates the hydrophobic amino acid F4 near the receptor protein (L39 and F52), enhancing shape compatibility. As a result, despite differences in sequences compared to the native peptide, DANTE effectively preserves a conformation akin to that of the native peptide while achieving improved stability and higher binding metrics through a careful balance of hydrophobic and hydrophilic interactions. Similar patterns are observed across other DANTE designs, with detailed comparisons provided in Supplementary Figures 39–48.

**DANTE settings:** In the context of cyclic peptide design, our approach incorporates a two-stage optimization process utilizing the DANTE. Initially, we generate a random peptide sequence to serve as the root node for the Monte Carlo tree. This stage involves conducting 15 rollouts from the initial node to explore potential sequences. Following this initial optimization phase, we identify and select three sequences for further analysis: these include the sequences with the highest and second-highest target values, as well as the sequence that was most frequently visited during the rollouts. These selected sequences then serve as the root nodes for a second round of tree search, during which another set of 15 rollouts is performed for each tree to refine our search for optimal sequences. In the final step of our optimization process, we compare the outcomes of the two iterations, selecting the sequence that demonstrates the highest target value as the optimized cyclic peptide sequence.

**Molecular dynamics simulation:** The MD simulations were performed based on the CHARMM36 forcefield and using NAMD engine. For each MD simulation, the complex was solvated in a truncated periodic TIP3P water box, and the minimum distance from the surfaces of the box to the complex atoms was set to 10 Å. Counter ions were added to neutralize systems, and the initial configuration was decided using a short Monte Carlo simulation. The Cyclic peptide topology and parameter file were generated using the ParamChem cyclic service. The simulation temperature was maintained at 303.15 K. 1.25 ns NVT (constant volume and temperature) was set for the equilibrating stage before 45 ns NPT (constant pressure and temperature) simulation with the time step of 0.1ns was performed. Root Mean Square Deviation (RMSD), Root Mean Square Fluctuation (RMSF) and

hydrogen bond analysis are performed by open-source package MDAnalysis. When calculating RMSD the backbone of the protein is aligned with the first frame of the molecular dynamic trajectory, while it is aligned with the average structure of all frames when calculating the RMSF.

## Real world task 4: Designing Monoclonal antibody against SARS-COVID-2

Recent advancements in protein structure prediction software, such as AlphaFold 3, have led to substantial increases in computational efficiency. This progress enables us to introduce a pertinent high-dimensional example of active learning in the context of protein design, which involves optimizing hundreds of amino acids. As achieving this optimization using AlphaFold 2, as utilized in our current project, presents considerable challenges. We present an example involving hundreds of dimensions, with AlphaFold 3 serving as the validation source (see Supplementary Fig. 49). In our analysis, we also tested several baseline methods, including TurBO5, MCMC, and DANTE.

Monoclonal antibodies (mAbs) are among the most common therapeutics and are frequently used for the treatment of cancer, autoimmune diseases, infectious diseases, and more. Recently, monobodies (Mobs) have gained attention as an alternative to mAbs due to their strong developability (*45*, *46*). Mobs are proteins roughly 100 amino acid residues in length, featuring an immunoglobulin (Ig)-like fold composed of 7 beta strands, derived from human Fibronectin type III (FN3). These proteins combine the advantageous features of both mAbs and miniproteins, which are generated through de novo design approaches, while also overcoming some of their disadvantages.

We build the Monobody binder design pipeline using DANTE, MCMC and TuRBO5 targeting at the receptor binding domain (RBD) of SARS-CoV-2 Spike protein. Similar to the real-world tasks proposed in the real-world task 3 in the main text, the pipeline uses AlphaFold 3, Rosetta and ProteinMPNN to generate pools of monobody binders with high ipTM score.

ipTM is a metric used to assess the accuracy of predicted relative positions in protein-protein complexes (*47*). Values greater than 0.8 indicate confident high-quality predictions, suggesting that the model has likely captured the correct orientation and interaction of the subunits. Conversely, values below 0.6 suggest a likely failed prediction, indicating potential inaccuracies in the model. ipTM values falling between 0.6 and 0.8 are considered a grey zone, where predictions could either be correct or erroneous, requiring further validation or additional evidence to ascertain their reliability.

As shown in Supplementary Fig. 50, the ipTM distributions obtained from different methods—DANTE, TurBO5, and MCMC—reveal that DANTE notably outperforms TurBO5 and generates a greater number of high-quality designs compared to MCMC. This indicates that DANTE is more effective at predicting the accurate relative positions of subunits in protein-protein complexes, leading to more reliable and successful design outcomes.

# Supplementary Figures

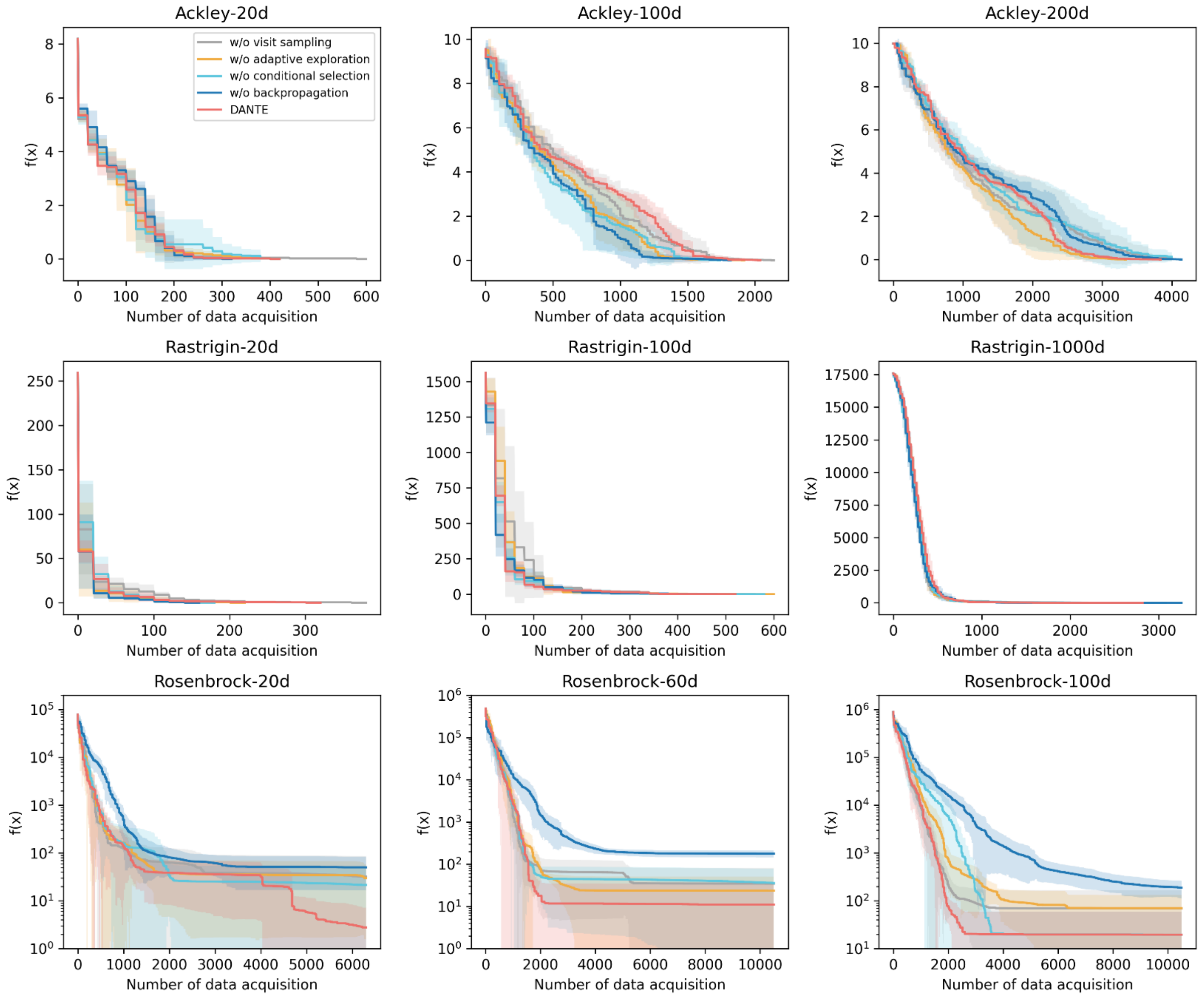


**Supplementary Figure 1: Ablation study of DANTE on synthetic functions with various configurations**

We assess the performance of the DANTE algorithm under various configurations (DANTE, without top-visit sampling, without adaptive exploration weight, without conditional selection, and without backpropagation) on the Ackley, Rastrigin, and Rosenbrock functions through surrogate model predictions. DANTE serves as the standard configuration. Without visit sampling entails the absence of selection based on the most visited nodes during each iteration, resulting in 18 samples chosen for their highest predictive values and 2 samples selected

randomly, with all other settings remaining default. Without conditional selection will replace the root node with the child node with the highest DUCB value in each rollout. Without adaptive exploration weight sets the exploration weight statically at 10, without any adjustments throughout the evaluation, while maintaining other default settings. Without backpropagation, also referred to as DANTE-Greedy, fixes the exploration weight at 0, with all other configurations default. In this figure, Ackley-20d means the tests on 20-dimension Ackley function. Data are presented as mean values ± SD, $n = 5$.

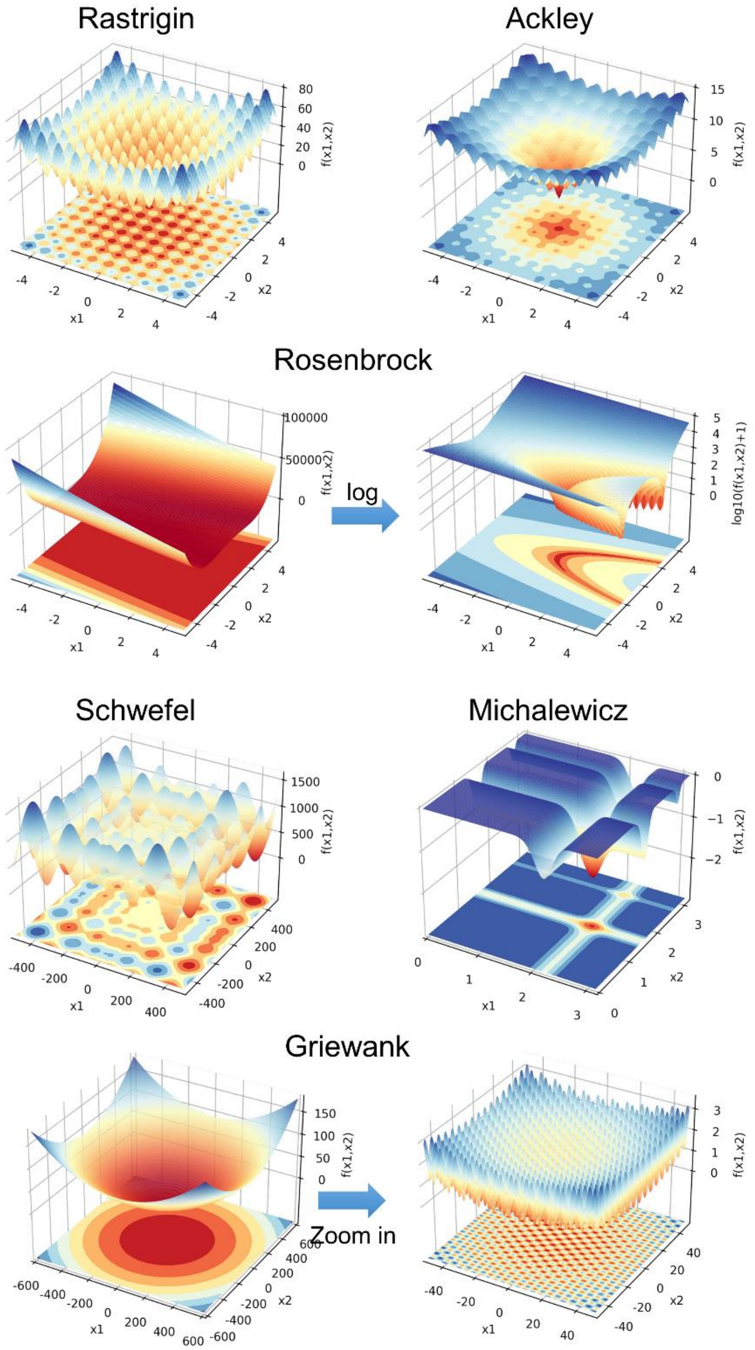


**Supplementary Figure 2: Distributions of the 2-D synthetic functions.**

Rastrigin, Ackley, Rosenbrock, Griewank, Schwefel, and Michalewicz functions in a 2-D form are displayed here. Among these, the Rastrigin, Ackley, Griewank, and Schwefel functions are characterized by numerous local minima. In contrast, the Rosenbrock function exhibits a valley-like shape, while the Michalewicz function resembles a steep ridge.

```python
from sklearn.model_selection import train_test_split
from tensorflow.keras.optimizers import Adam
from tensorflow.keras.models import Sequential
from tensorflow.keras.layers import Conv1D, MaxPooling1D, Flatten, Dense,Dropout
from tensorflow.keras.callbacks import EarlyStopping
from tensorflow.keras import layers

# Input: (None, dimension, 1)
X_train, X_test, y_train, y_test = train_test_split(X, y, test_size=0.2, random_state=i)

# model architecture

# for Ackley
model = Sequential()
model.add(Conv1D(128, kernel_size=3, strides=1, padding='same', activation='elu',
                 input_shape=(dims, 1))) #dims is the dimension
model.add(MaxPooling1D(pool_size=2, strides=1))
model.add(Dropout(0.2))
model.add(Conv1D(64,kernel_size=3, strides=1, padding='same', activation='elu'))
model.add(MaxPooling1D(pool_size=2, strides=1))
model.add(Dropout(0.2))
model.add(Conv1D(32, kernel_size=3, strides=1, padding='same', activation='elu'))
model.add(Conv1D(16, kernel_size=3, strides=1, padding='same', activation='elu'))
model.add(Conv1D(8, kernel_size=3, strides=1, padding='same', activation='elu'))
model.add(Flatten())
model.add(Dense(128,activation='elu'))
model.add(Dense(64,activation='elu'))
model.add(Dense(1,activation='linear'))
model.compile(optimizer=Adam(learning_rate=0.001), loss='mean_squared_error')

# for Rastrigin
model = Sequential([
    layers.Conv1D(256,kernel_size=5,strides=1,padding='same', activation='elu',
                  input_shape=(dims,1)),
    layers.LayerNormalization(),
    layers.Conv1D(128,kernel_size=5,strides=2, padding='same', activation='elu'),
    layers.Conv1D(64,kernel_size=3,strides=2, padding='same', activation='elu'),
    layers.Conv1D(32,kernel_size=3,strides=1, padding='same', activation='elu'),
    layers.Conv1D(16,kernel_size=3,strides=1, padding='same', activation='elu'),
    layers.Conv1D(8,kernel_size=3,strides=1, padding='same', activation='elu'),
    layers.Flatten(),
    Dense(128, activation='elu'),
    Dense(64, activation='elu'),
    Dense(1, activation='linear')
])
model.compile(optimizer=Adam(learning_rate=0.001), loss='mean_absolute_percentage_error')

# for Rosenbrock
model = Sequential()
model.add(Conv1D(128, kernel_size=3, strides=1, padding='same', activation='elu',
                 input_shape=(dims, 1)))
model.add(MaxPooling1D(pool_size=2))
model.add(Dropout(0.2))
model.add(Conv1D(64, kernel_size=3, strides=1, padding='same', activation='elu'))
model.add(MaxPooling1D(pool_size=2))
model.add(Dropout(0.2))
model.add(Conv1D(32, kernel_size=3, strides=1, padding='same', activation='elu'))
model.add(MaxPooling1D(pool_size=2, strides=1))
model.add(Conv1D(16, kernel_size=3, strides=1, padding='same', activation='elu'))
model.add(Conv1D(8, kernel_size=3, strides=1, padding='same', activation='elu'))
model.add(Conv1D(4, kernel_size=3, strides=1, padding='same', activation='elu'))
model.add(Flatten())
model.add(Dense(64,activation='elu'))
model.add(Dense(1,activation='linear'))
model.compile(optimizer=Adam(learning_rate=0.001), loss='mean_squared_error')

# trainning model
early_stop = EarlyStopping(monitor='val_loss', patience=30, restore_best_weights=True)
model.fit(X_train.reshape(len(X_train),dims,1), y_train, batch_size=64, epochs=500,
          validation_data=(X_test.reshape(len(X_test),dims,1), y_test), callbacks=[early_stop])
```

**Supplementary Figure 3: Model architecture of 1D-CNN for evaluations on synthetic functions**

This figure details the model architectures of 1D convolutional neural networks used for evaluations on Ackley, Rastrigin, and Rosenbrock functions. The hyperparameters and model architectures were empirically optimized to ensure good model performance. It is important to note that the model architecture is exclusively related to the synthetic function and is independent of the optimization algorithm, with the exception that TuRBO5 and LAMCTS default to using Support Vector Machines (SVM).

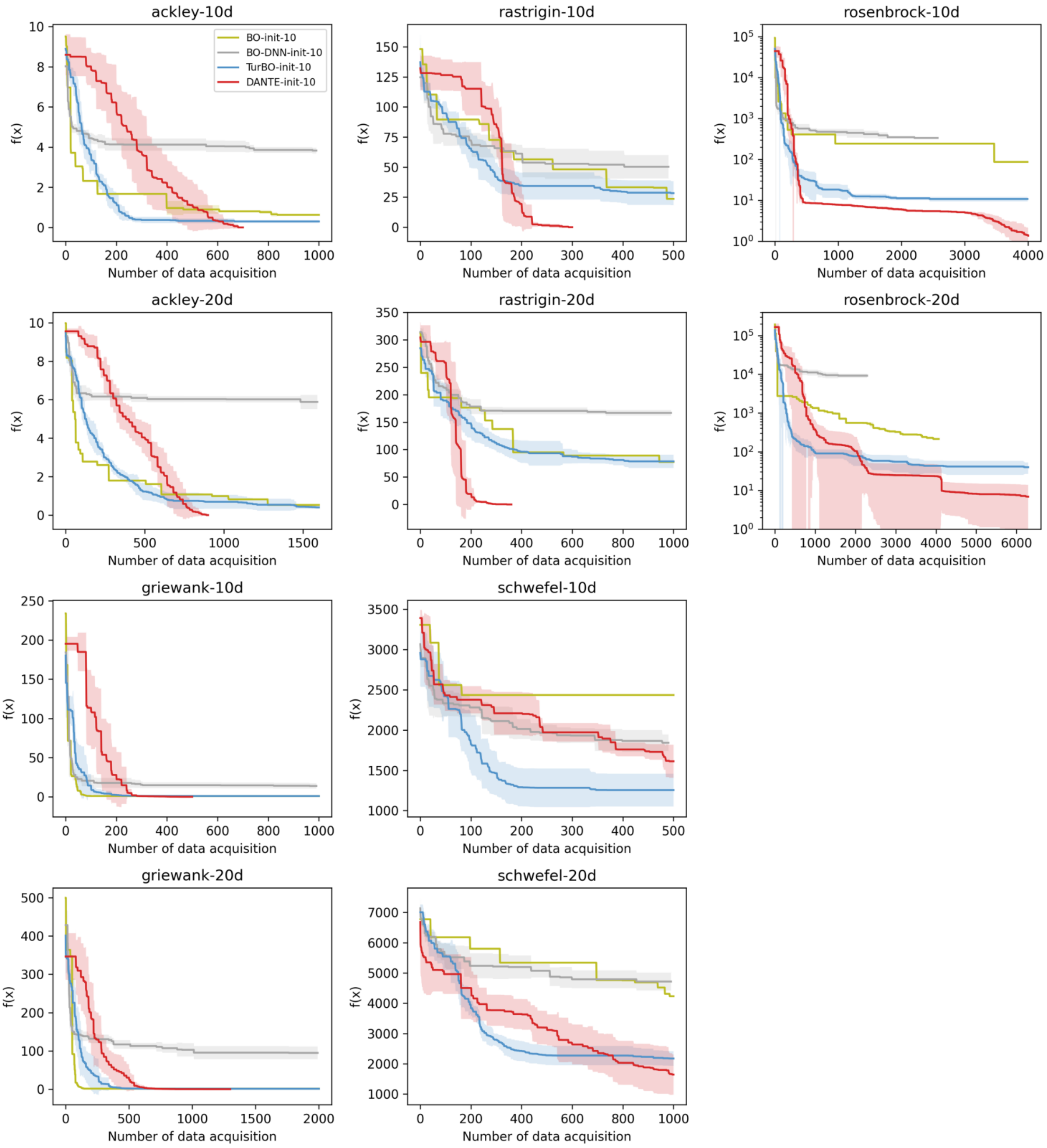


**Supplementary Figure 4: Evaluations on synthetic functions at initial dataset = 10**

Test functions are Ackley-10d, 20d; Rastrigin-10d, 20d; Rosenbrock-10, 20d; Griewank-10d, 20d; Schwefel-10d, 20d. Selected algorithms: Vanilla BO, TurBO5, BO-CNN and DANTE. Data are presented as mean values ± SD, $n = 5$.

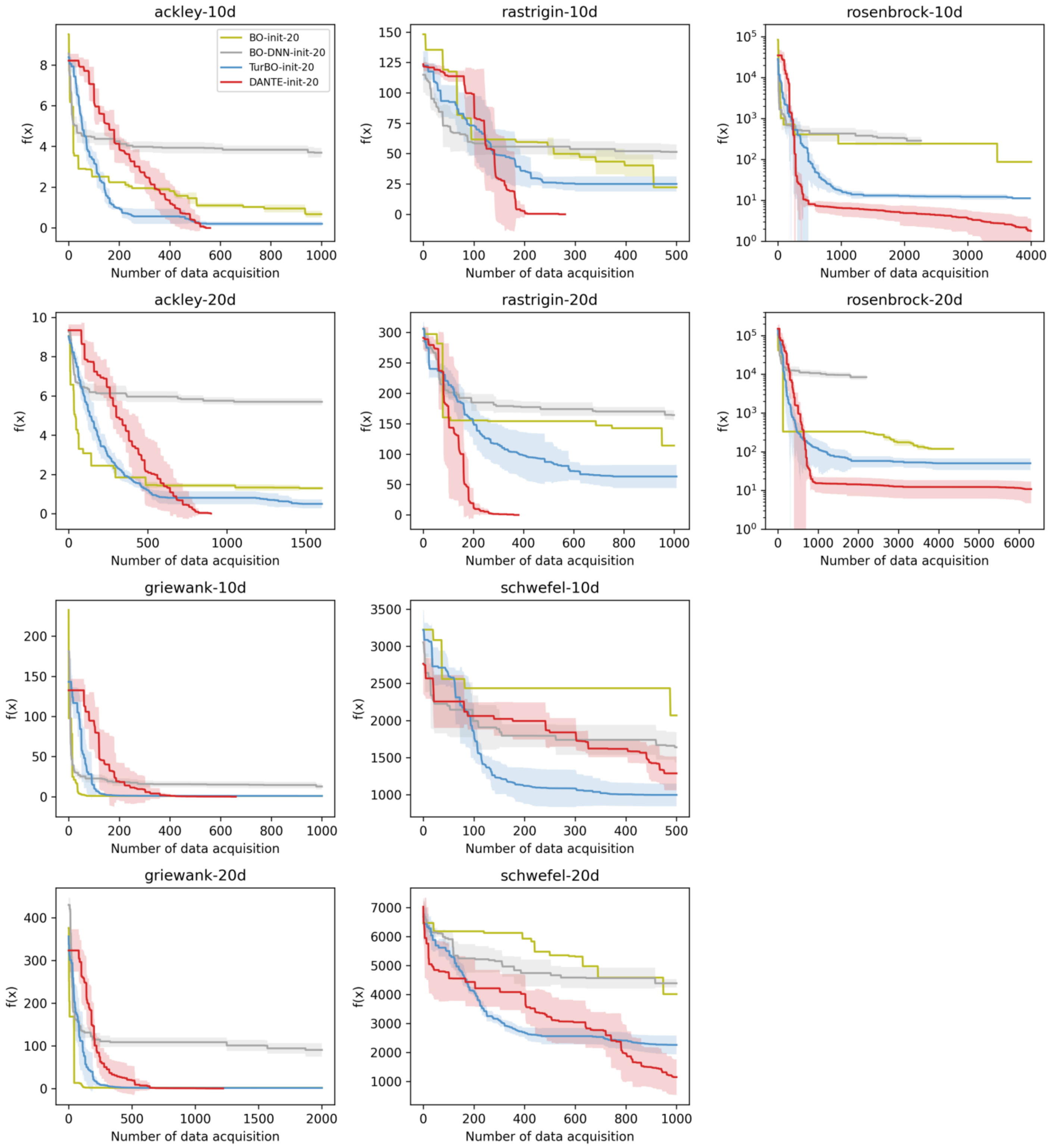


**Supplementary Figure 5: Evaluations on synthetic functions at initial dataset = 20**

Test functions are Ackley-10d, 20d; Rastrigin-10d, 20d; Rosenbrock-10, 20d; Griewank-10d, 20d; Schwefel-10d, 20d. Selected algorithms: Vanilla BO, TurBO5, BO-CNN and DANTE. Data are presented as mean values ± SD, $n = 5$.

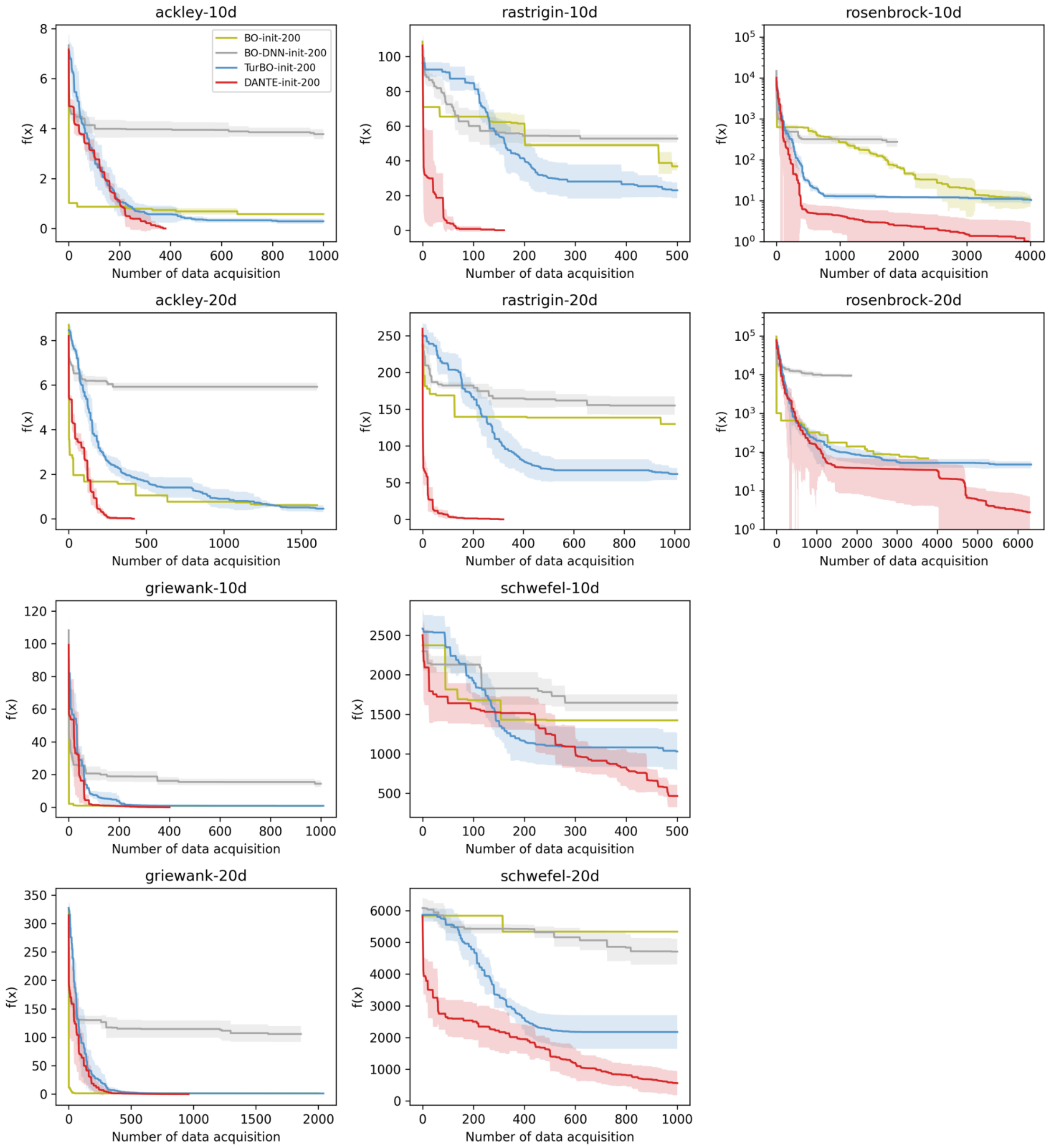


**Supplementary Figure 6: Evaluations on synthetic functions at initial dataset = 200**

Test functions are Ackley-10d, 20d; Rastrigin-10d, 20d; Rosenbrock-10, 20d; Griewank-10d, 20d; Schwefel-10d, 20d. Selected algorithms: Vanilla BO, TurBO5 (State-of-the-art BO-variant), BO-CNN and DANTE. Data are presented as mean values ± SD, $n = 5$.

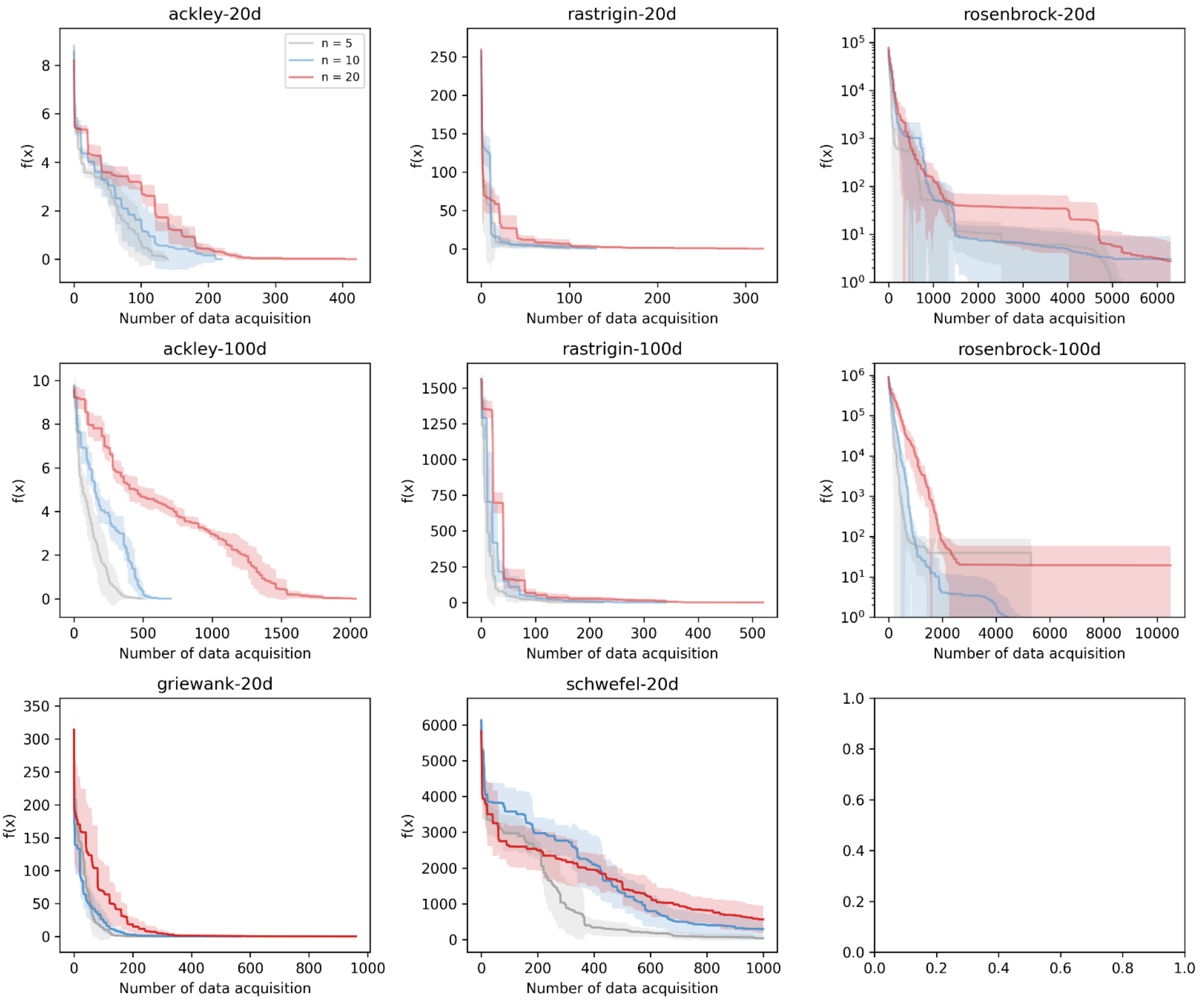


**Supplementary Figure 7: Evaluations of DANTE on synthetic functions with different sampling batch sizes**

Test functions are Ackley-20d, 100d; Rastrigin-20d, 100d; Rosenbrock-20, 100d; Griewank-20d; Schwefel-20d. Data are presented as mean values ± SD, $n = 5$.

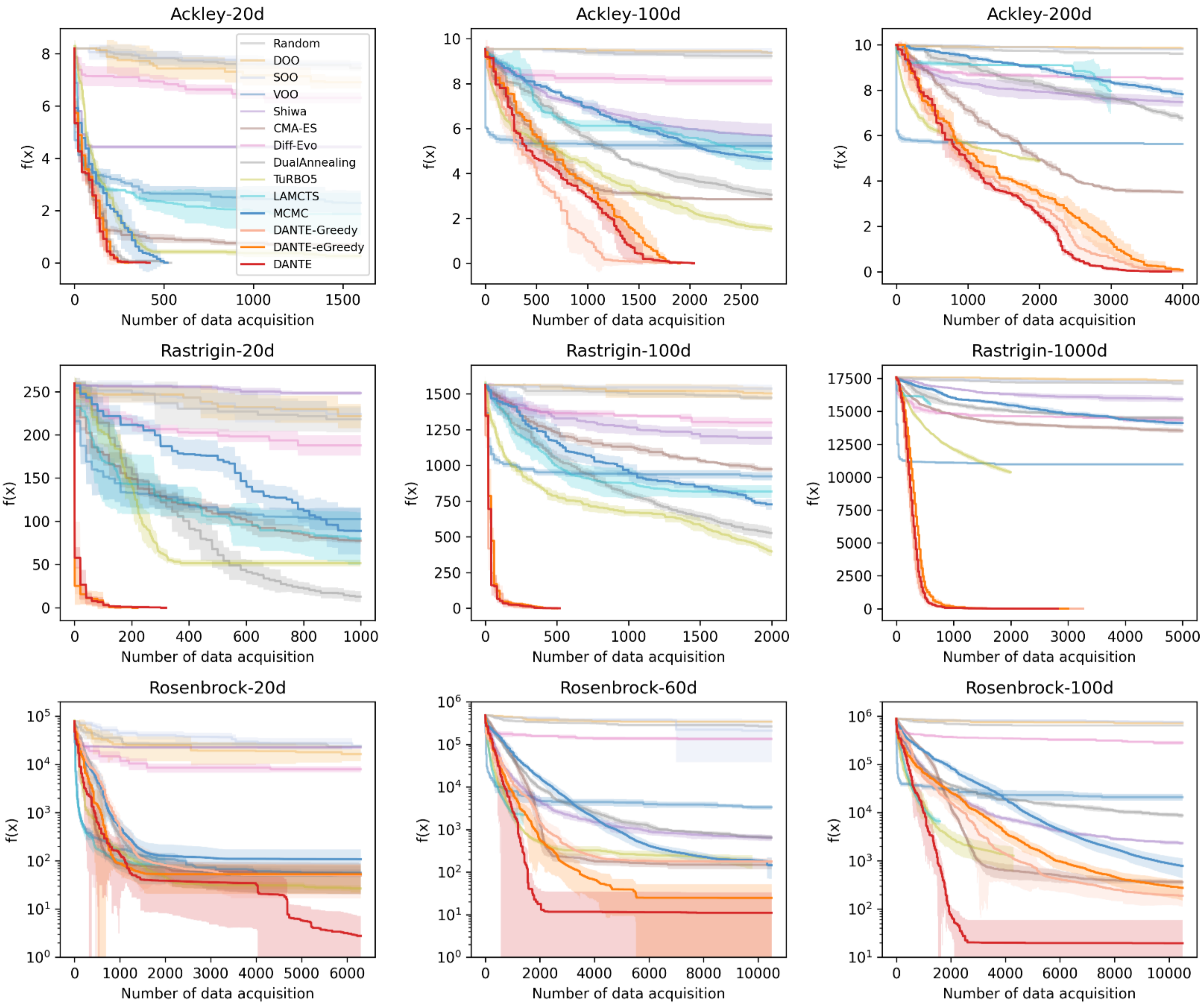


**Supplementary Figure 8: Evaluations on synthetic functions using surrogate model**

We run extensive tests on well-known non-convex functions (Ackley, Rastrigin, Rosenbrock) of diverse types using surrogate model predictions and compare the performance of DANTE with other state-of-the-art algorithms. DANTE-based methods outperform other benchmark methods. In this figure, Ackley-20d means the tests on 20-dimension Ackley function. Supplementary Fig. 46 shows the curve of each evaluation on Rosenbrock. Data are presented as mean values ± SD, $n = 5$.

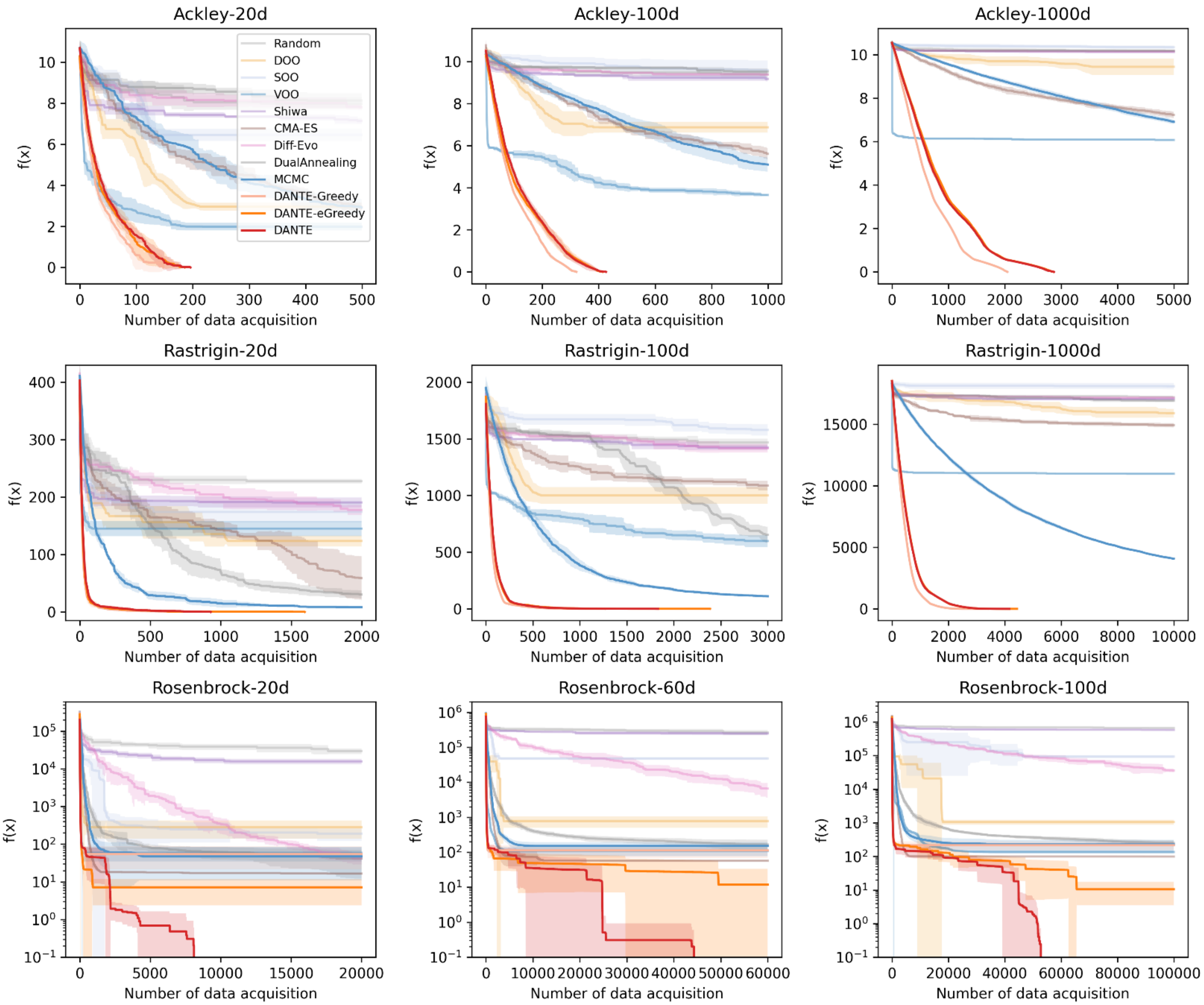


**Supplementary Figure 9: Evaluations on synthetic functions (Ackley, Rastrigin, Rosenbrock) using exact function**

We run extensive evaluations on Ackley, Rastrigin, and Rosenbrock functions using exact function. DANTE outperforms other benchmark methods. In this figure, Ackley-20d means the tests on 20-dimension Ackley function. Data are presented as mean values ± SD, $n = 5$.

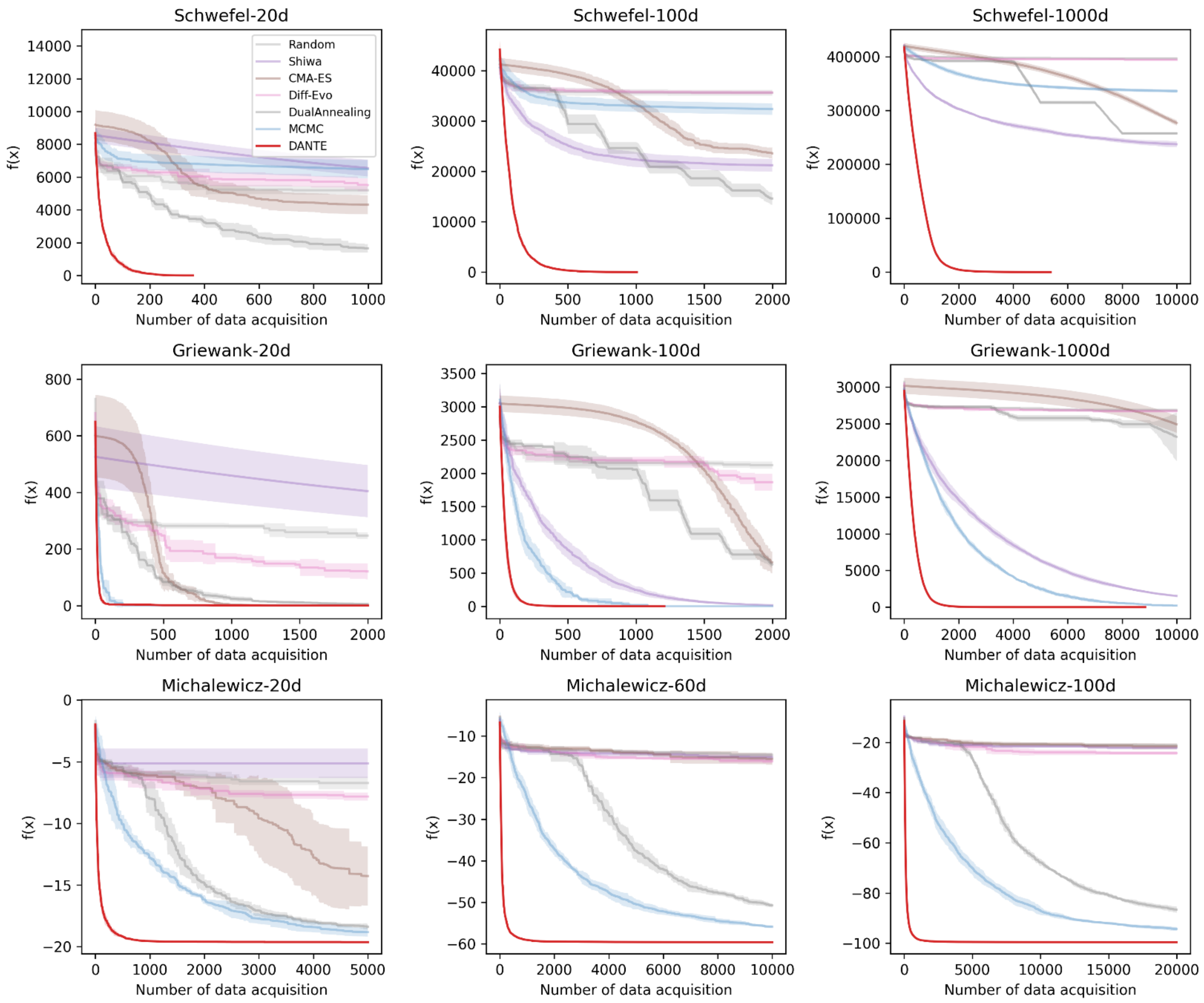


**Supplementary Figure 10: Evaluations on synthetic functions (Schwefel, Griewank, Michalewicz) using exact function**

We also run extensive evaluations on Schwefel, Griewank, and Michalewicz functions using exact function, where DANTE demonstrated superior performance compared to other benchmarked methods. In this context, "Schwefel-20d" refers to the tests conducted on the 20-dimensional Schwefel function. Data are presented as mean values ± SD, $n = 5$.

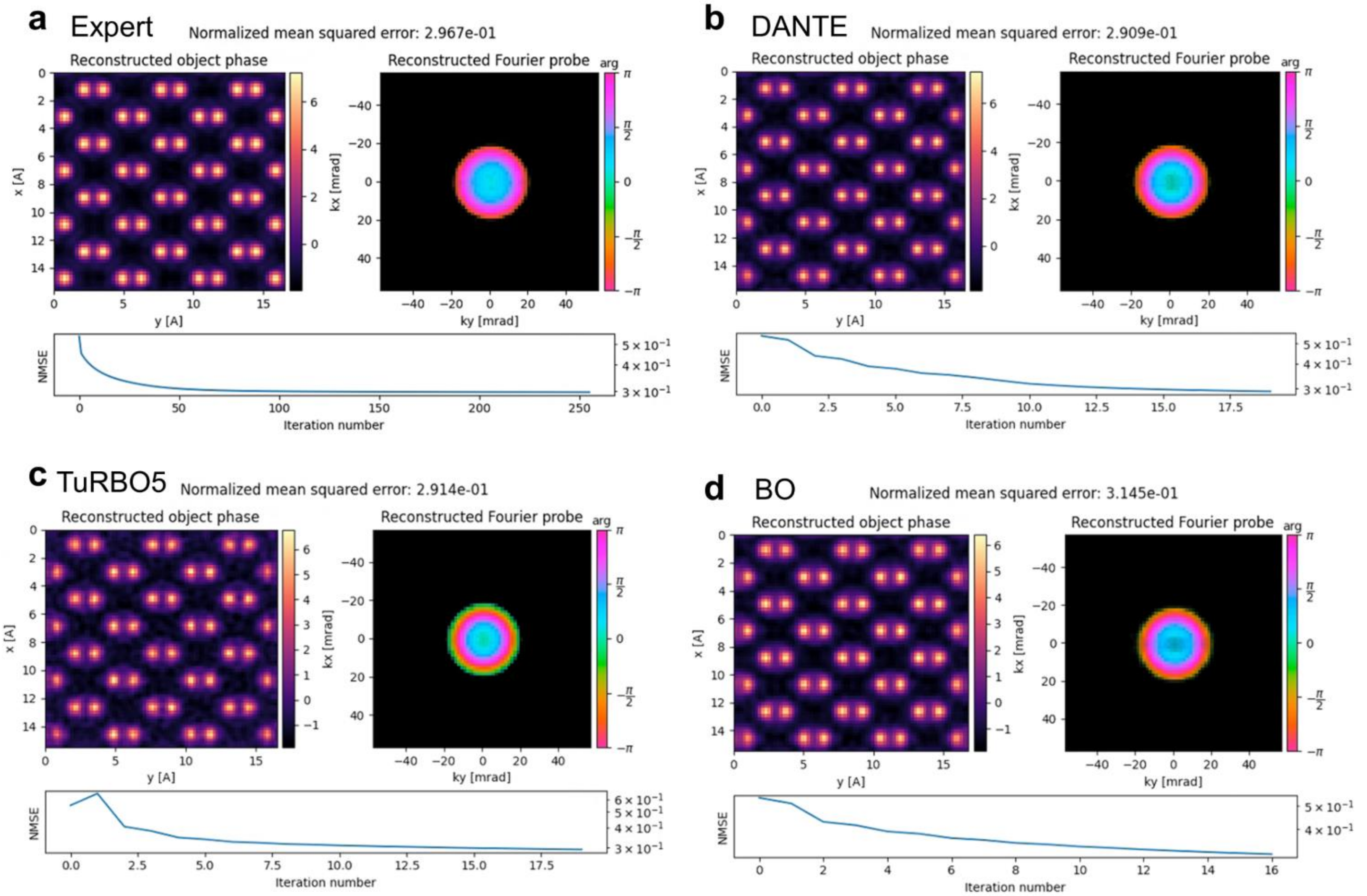


**Supplementary Figure 11: Electron ptychography reconstruction process**

Reconstructed object phase, reconstructed Fourier probe, and NMSE evolution curve while reconstruction using py4DSTEM of (**a**) expert, (**b**) DANTE, (**c**) TuRBO5, and (**d**) BO.

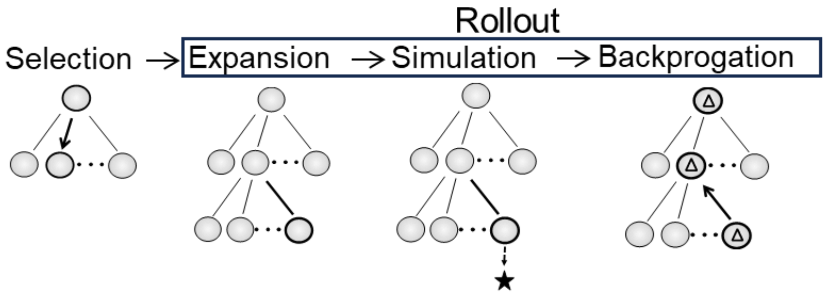


**Supplementary Figure 12: Phases of the Monte Carlo tree search algorithm**

Monte Carlo tree search (MCTS) is grown through repeated application of the above four phases: selection, expansion, simulation, and backpropagation.

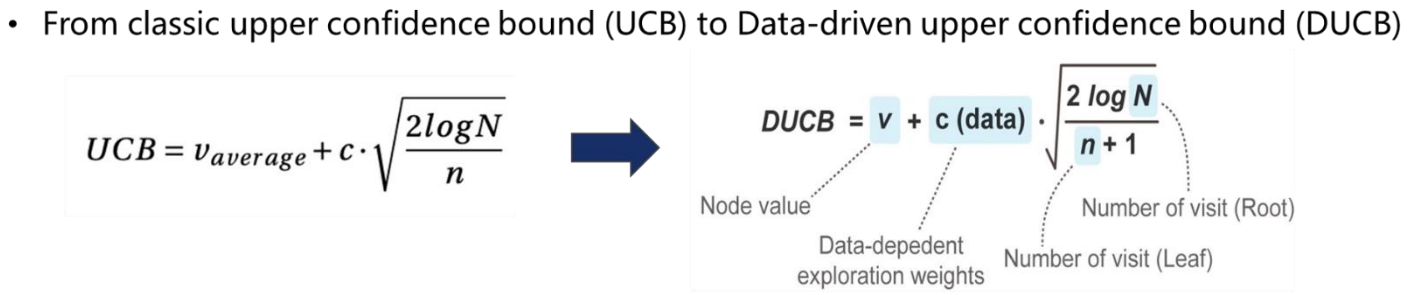


| | Node value | Weight | Backprogation | Exploration |
|---|---|---|---|---|
| **DUCB** | • **Machine learning**<br>• **Trajectory-independent** | **Adaptive** | • **Short range**<br>• **# of visit only** | **Finite value** |
| UCB | • Large # of Simulation<br>• Trajectory-dependent | Constant | • Long range<br>• # of visit & value | Infinity everywhere |

**Supplementary Figure 13: The key difference between UCB and DUCB.**

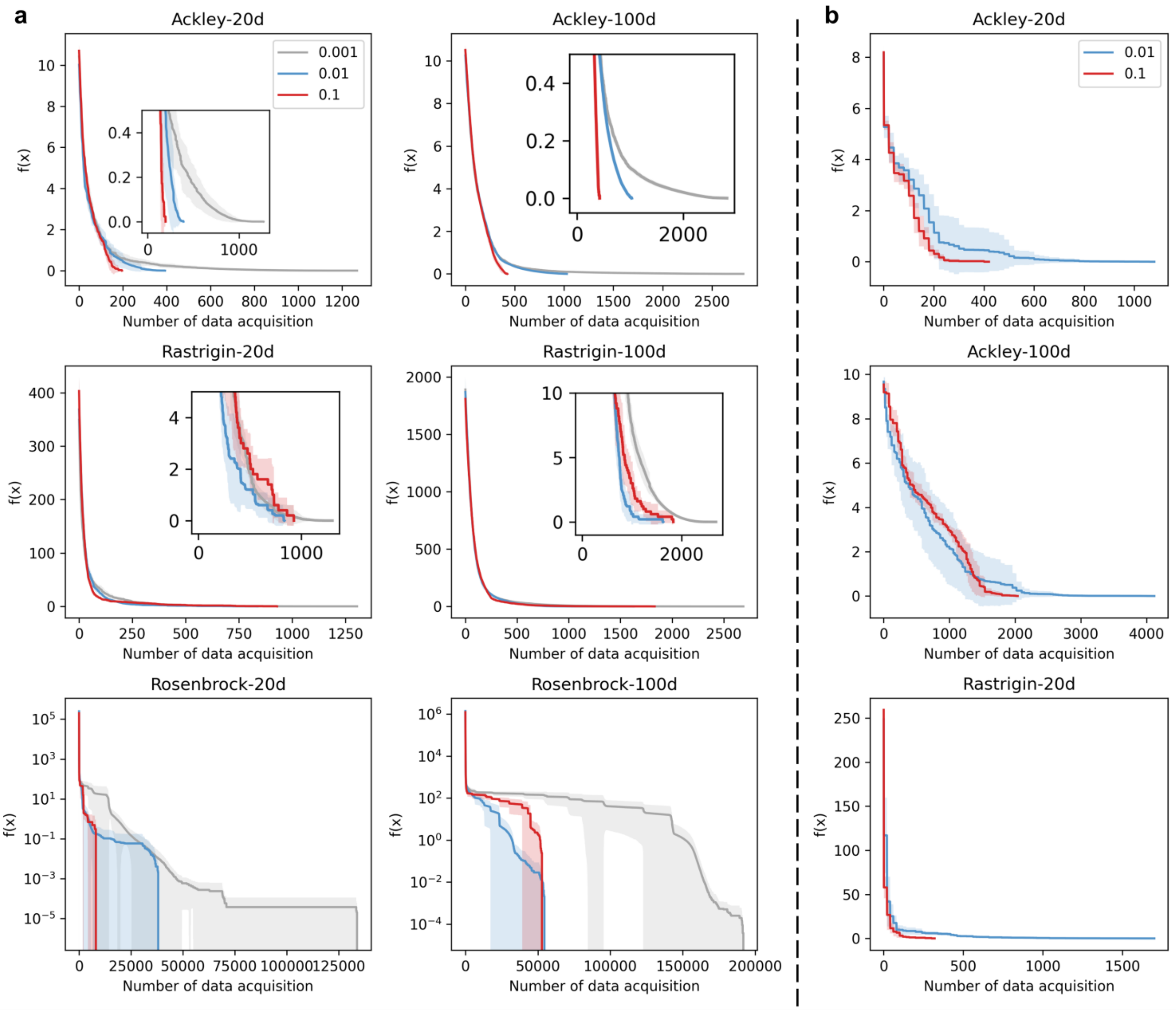


**Supplementary Figure 14: Ablation study of DANTE on synthetic functions with different intervals**

(**a**) exact functions, (**b**) surrogate model predictions. Data are presented as mean values ± SD, $n = 5$.

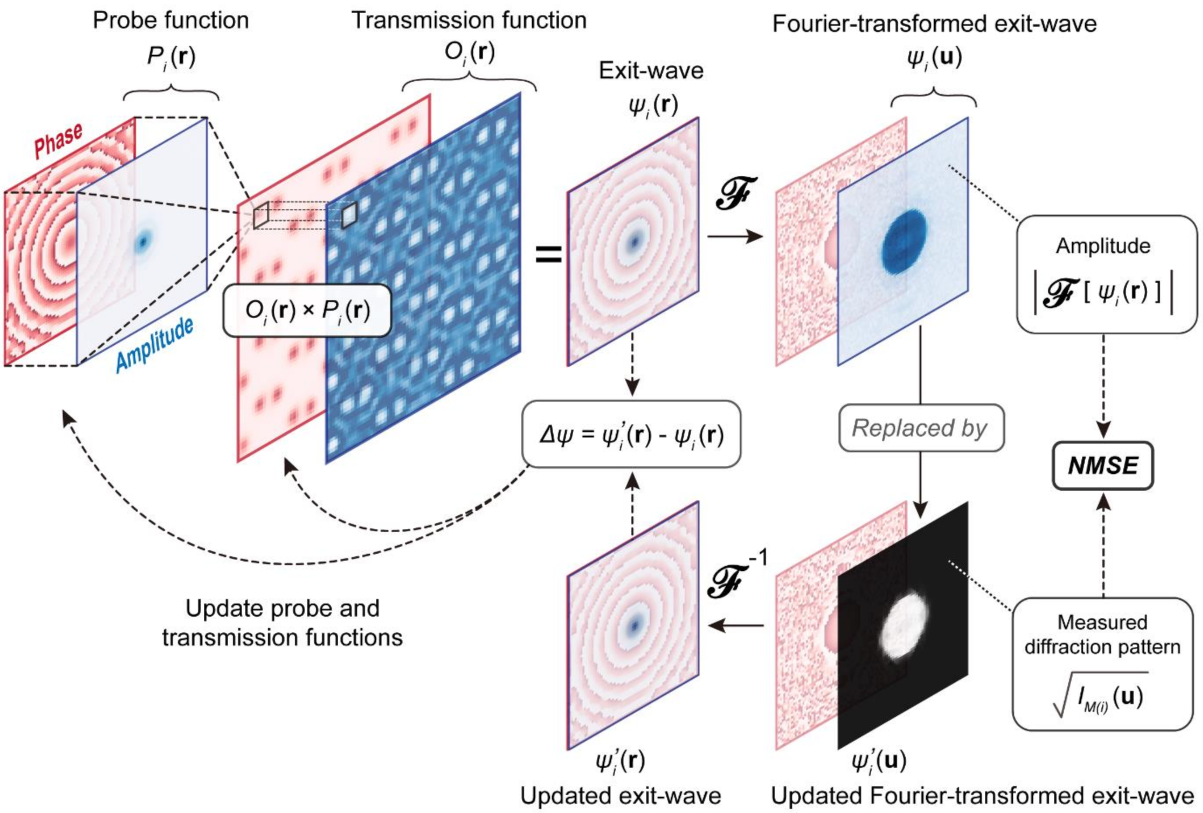


**Supplementary Figure 15: Ptychographic reconstruction procedure and the computing of normalized mean squared error.**

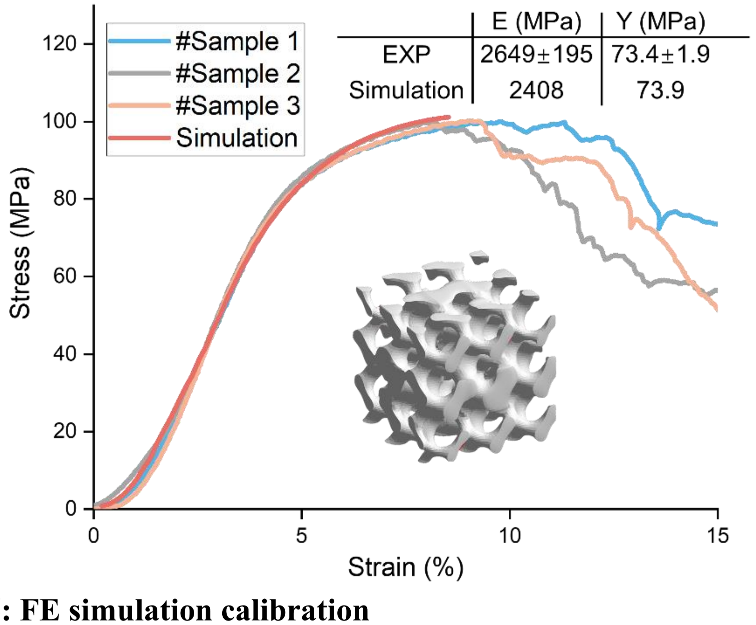


**Supplementary Figure 16: FE simulation calibration**

The simulation curve agrees well with the experiment compression curves. Three replicates were tested to ensure reproducibility. The error of the elastic modulus (E) and yield strength (Y) between the FE simulation and experimental results is less than 10%.

```
from tensorflow.keras import layers
from tensorflow import keras
from sklearn.model_selection import train_test_split
from tensorflow.keras.optimizers import Adam
from tensorflow.keras.callbacks import EarlyStopping

# Input: (None, 60, 60, 60, 1)
X_train, X_test, y_train, y_test = train_test_split(X, y, test_size=0.2, random_state=i)

# model architecture
inputs = keras.Input((60, 60, 60, 1))
x = layers.Conv3D(filters=8, kernel_size=3, activation="elu",padding='same')(inputs)
x = layers.MaxPool3D(pool_size=2,padding='same')(x)
x = layers.Conv3D(filters=4, kernel_size=3, activation="elu",padding='same')(x)
x = layers.MaxPool3D(pool_size=2,padding='same')(x)
x = layers.Conv3D(filters=2, kernel_size=3, activation="elu",padding='same')(x)
x = layers.MaxPool3D(pool_size=2,padding='same')(x)
x = layers.Flatten()(x)
x = layers.Dense(units=128, activation="elu")(x)
x = layers.Dense(units=64, activation="elu")(x)
x = layers.Dense(units=32, activation="elu")(x)
outputs = layers.Dense(units=1, activation="linear")(x)
model = keras.Model(inputs, outputs, name="3dcnn")

# model compiling and training
early_stop = EarlyStopping(monitor='val_loss', patience=100, restore_best_weights=True)
model.compile(optimizer=Adam(learning_rate=0.001), loss='mean_squared_error')
model.fit(X_train, y_train, batch_size=32, epochs=5000, validation_data=(X_test, y_test),
          callbacks=[early_stop])
```

**Supplementary Figure 17: Model architecture of 3D-CNN for architected material property predictions**

This figure details the model architectures of 3D convolutional neural networks used for elastic modulus and yield strength predictions of architected materials. The hyperparameters and model architectures were determined by trial and error.

```python
from sklearn.model_selection import train_test_split
from tensorflow.keras.optimizers import Adam
from tensorflow.keras.models import Sequential
from tensorflow.keras.callbacks import EarlyStopping
from tensorflow.keras import layers
from tensorflow import keras

# Input: (None, 27, 1)
X_train, X_test, y_train, y_test = train_test_split(X, y, test_size=0.2, random_state=i)

# model architecture
model = Sequential([
    layers.Conv1D(64,kernel_size=3,strides=2,padding='same', activation='elu', input_shape=(27,1)),
    layers.BatchNormalization(),
    layers.Conv1D(32,kernel_size=3,strides=2, padding='same', activation='elu'),
    layers.Conv1D(16,kernel_size=3,strides=2, padding='same', activation='elu'),
    layers.Dropout(0.2),
    layers.Conv1D(8,kernel_size=3,strides=1, padding='same', activation='elu'),
    layers.Flatten(),
    layers.Dense(128, activation='elu'),
    layers.Dense(1, activation='linear')
])

# model compiling and training
optimizer = keras.optimizers.Adam(learning_rate=0.001)
model.compile(optimizer=optimizer, loss='mse', metrics=["mean_squared_error"])
es = EarlyStopping(monitor='val_loss', mode='min', verbose=1,patience=1000)
model.fit(X_train, y_train, validation_data=(X_test, y_test),  batch_size=50, epochs=5000,
          callbacks=[es])
```

**Supplementary Figure 18: Model architecture of 1D-CNN for CCA property predictions**

This figure details the model architectures of 1D convolutional neural networks used for formation energy, AHA, and AHC predictions of CCAs. The hyperparameters and model architectures were determined by trial and error.

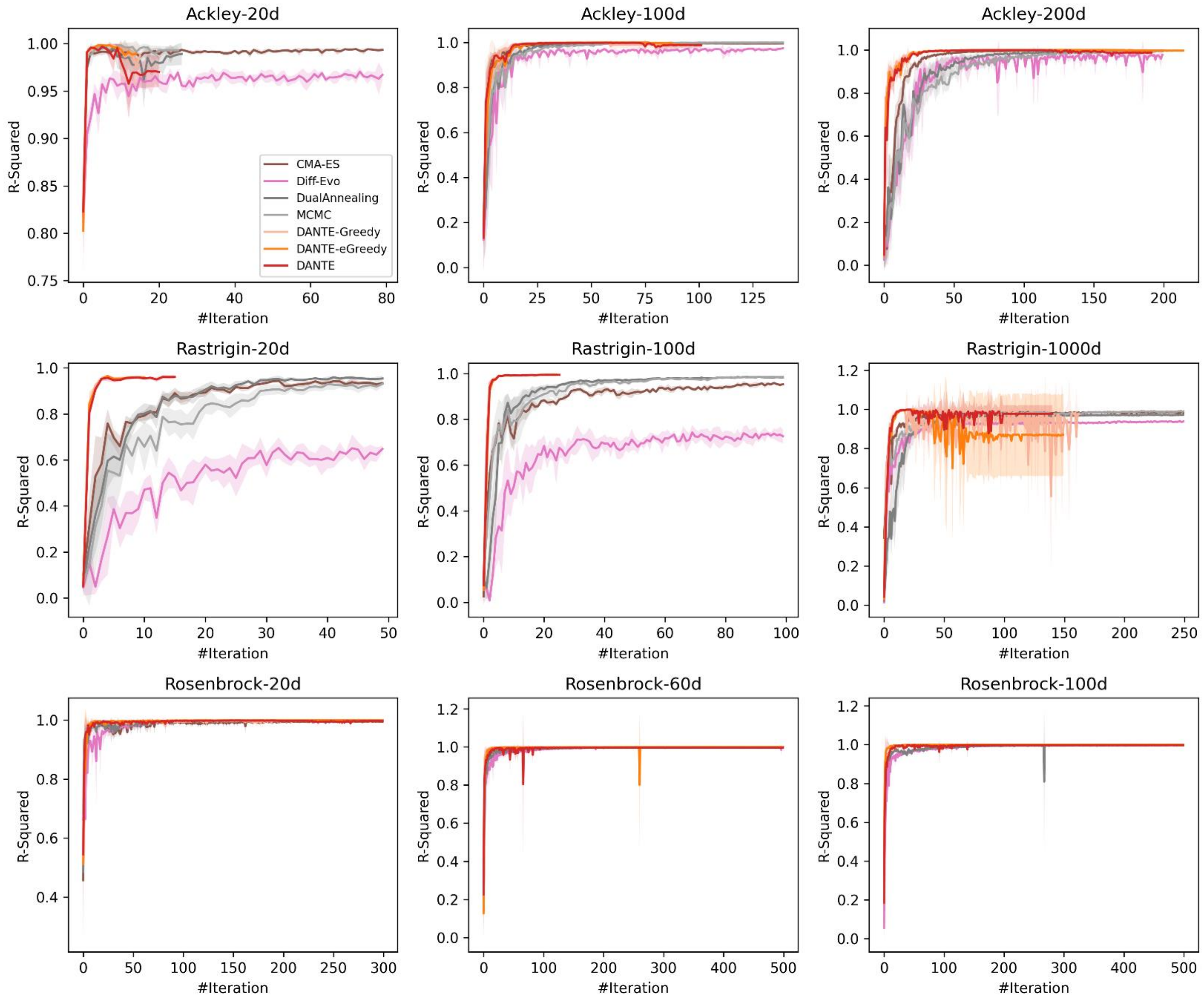


**Supplementary Figure 19: $R^2$ versus number of iterations while evaluations on synthetic functions using surrogate model.**

Data are presented as mean values ± SD, $n = 5$.

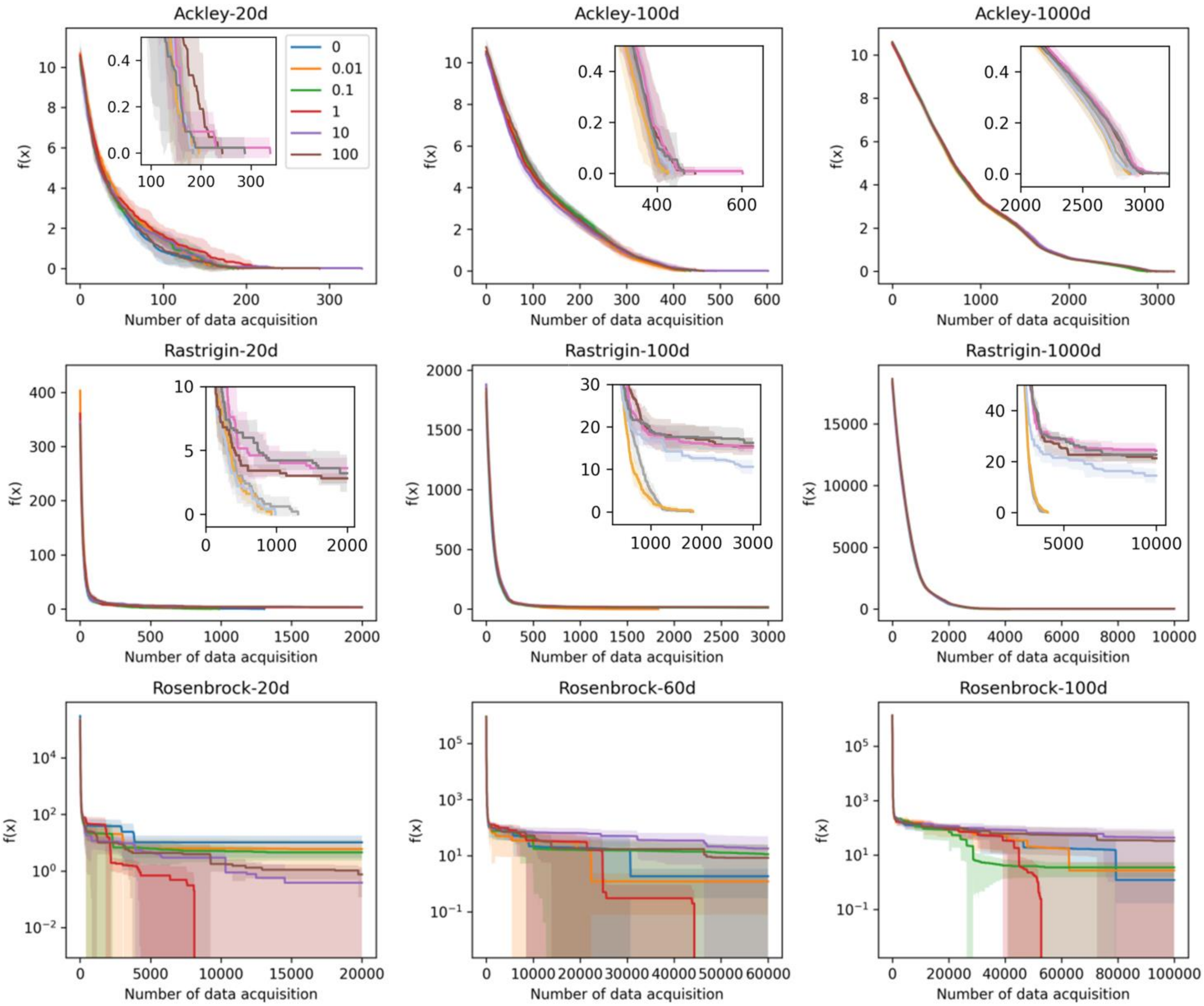


**Supplementary Figure 20: Ablation study of DANTE on synthetic functions with different exploration weight ratios $c_0$**

We evaluated the performance of the DANTE algorithm across different exploration weight ratios on the Ackley, Rastrigin, and Rosenbrock functions, using exact function. The default ratios are set at 0.01 for Ackley, Rastrigin, Griewank and Schwefel functions, 0.1 for Rosenbrock and Michalewicz functions. Data are presented as mean values ± SD, $n = 5$.

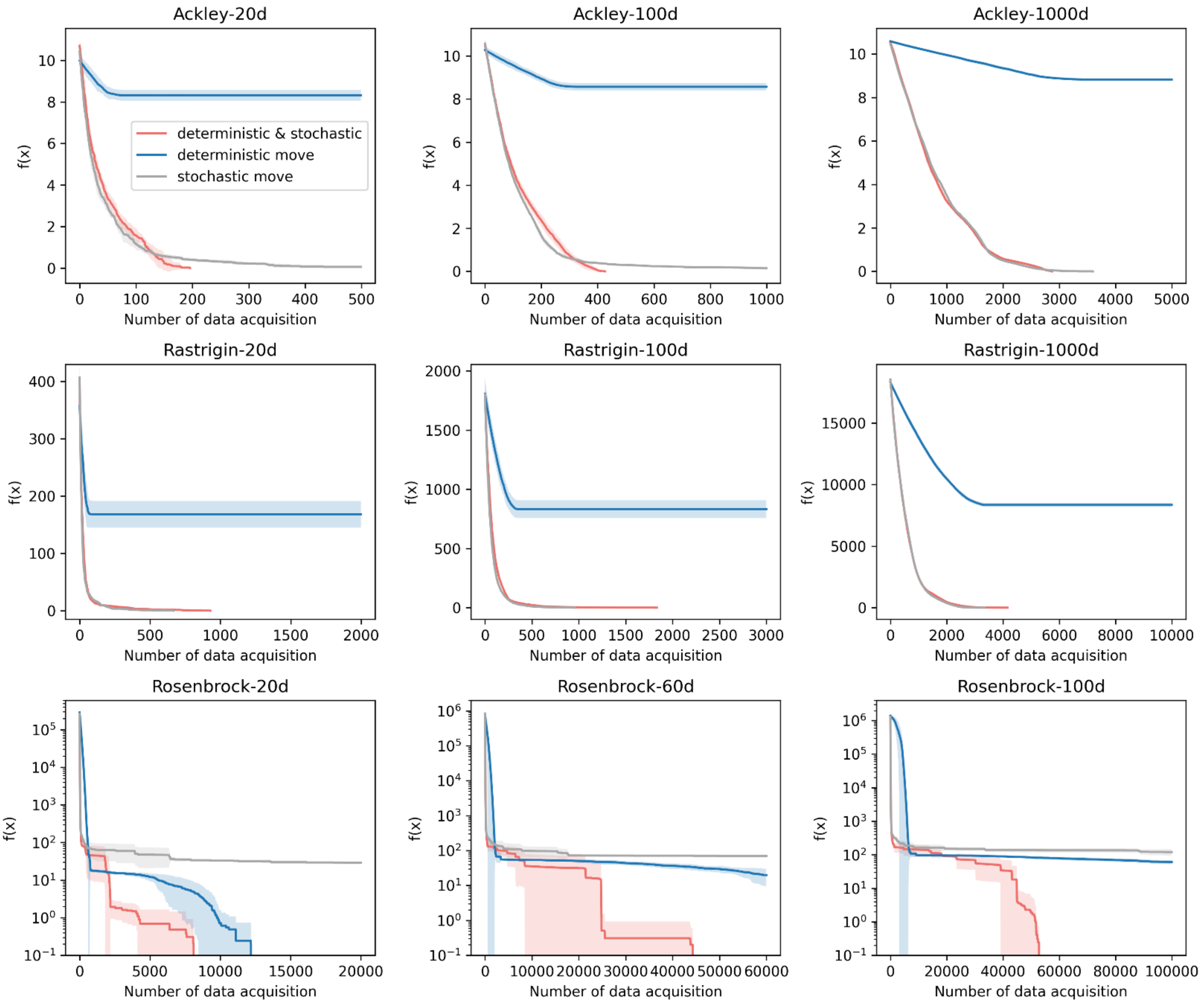


**Supplementary Figure 21: Ablation study of DANTE on synthetic functions with different expansion actions**

We evaluated the DANTE algorithm's performance across different expansion actions—stochastic expansion, deterministic expansion, and a combination of both—on the Ackley, Rastrigin, and Rosenbrock functions using exact function. Utilizing both expansion strategies represents the standard configuration for DANTE. Data are presented as mean values ± SD, $n = 5$.

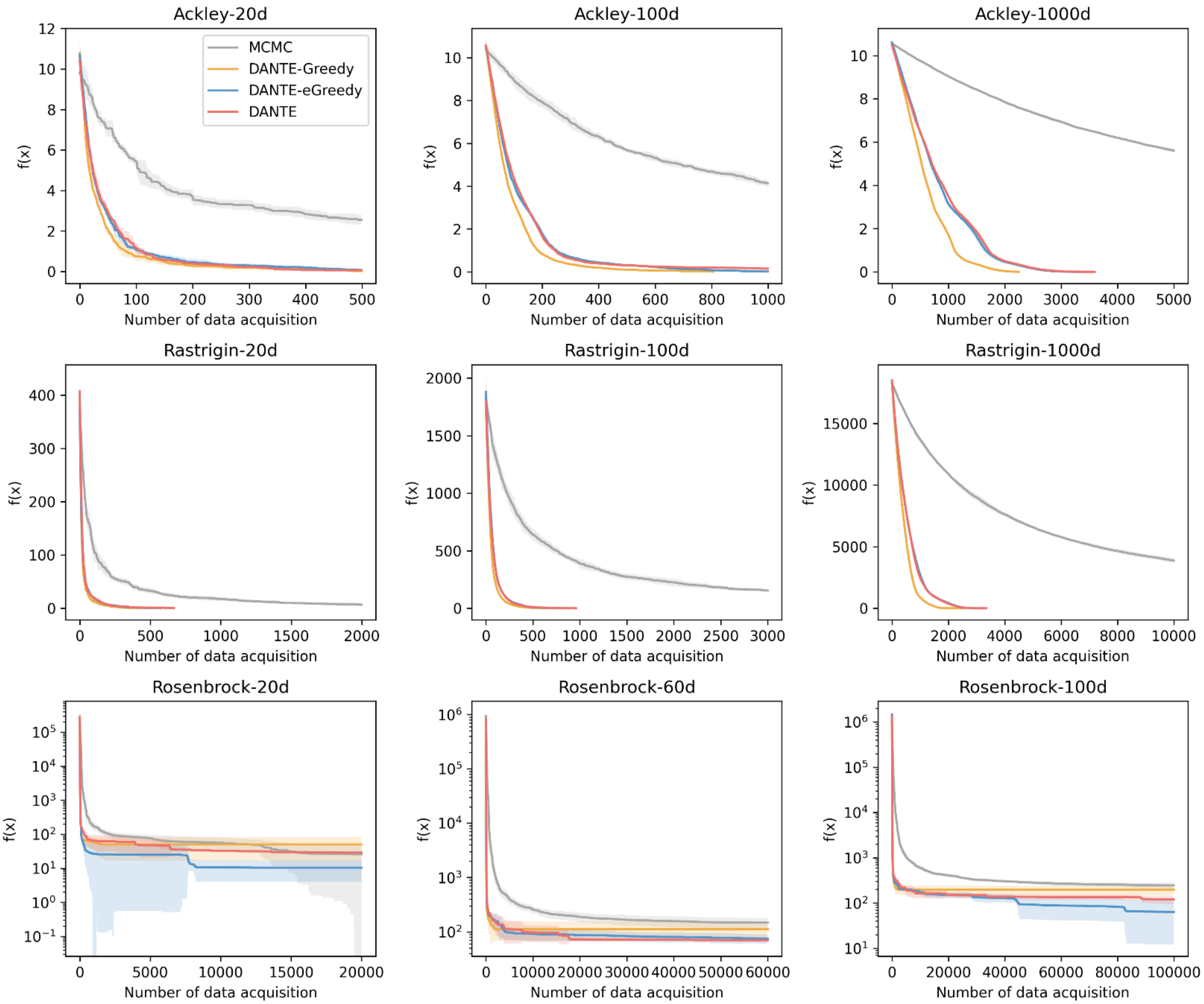


**Supplementary Figure 22: Evaluations on synthetic functions with only stochastic expansion**

We run DANTE variants with only stochastic moves on Ackley, Rastrigin, and Rosenbrock functions using exact function. Data are presented as mean values ± SD, $n = 5$.

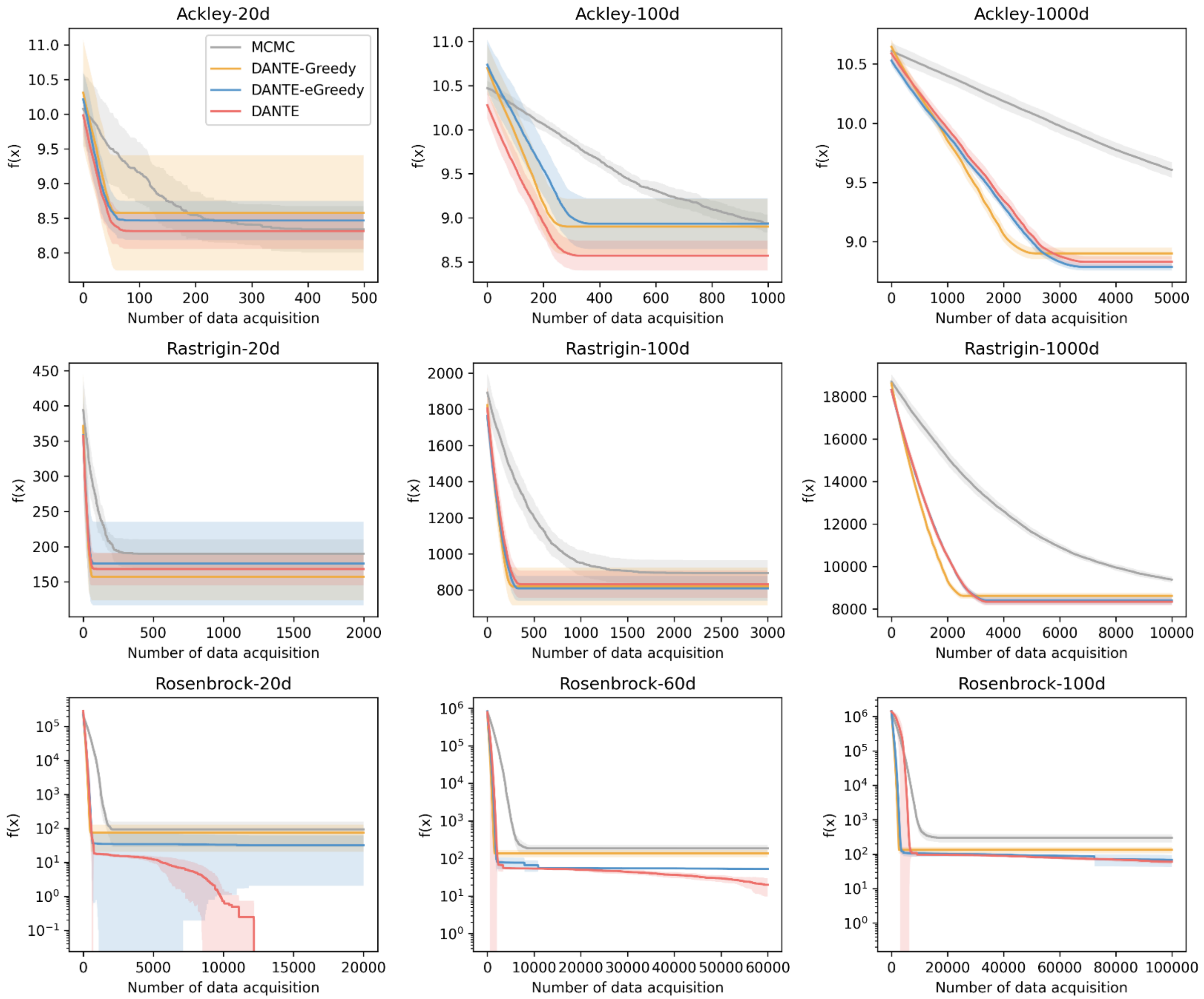


**Supplementary Figure 23: Evaluations on synthetic functions with only deterministic expansion**

We run DANTE variants with only deterministic moves on Ackley, Rastrigin, and Rosenbrock functions using exact function. Data are presented as mean values ± SD, $n = 5$.

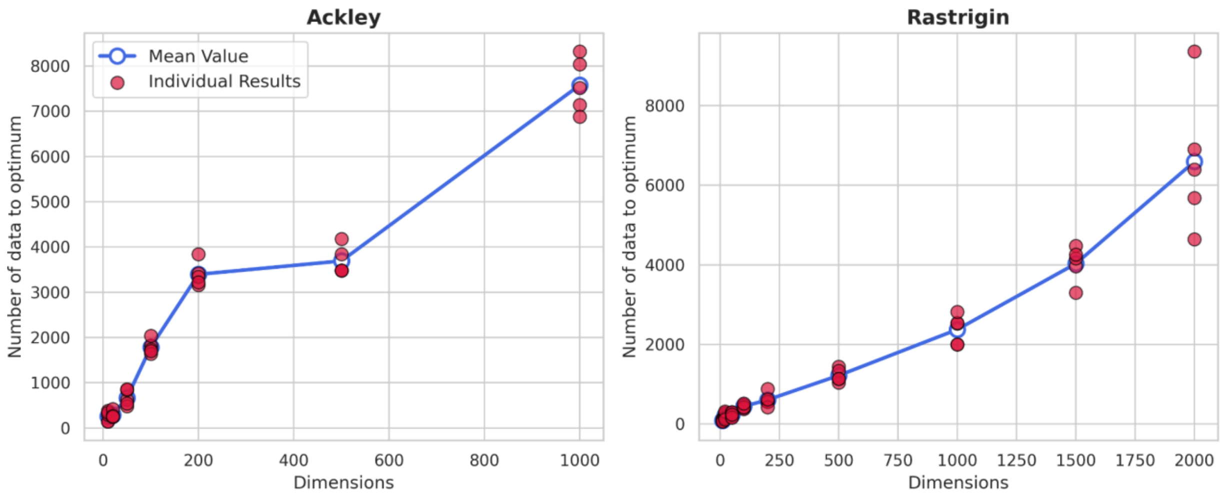


**Supplementary Figure 24: Dimensions versus the number of data points needed to achieve the optimum**

We evaluated DANTE on Ackley and Rastrigin functions with different dimensions. Data are presented as mean values ± SD, $n = 5$.

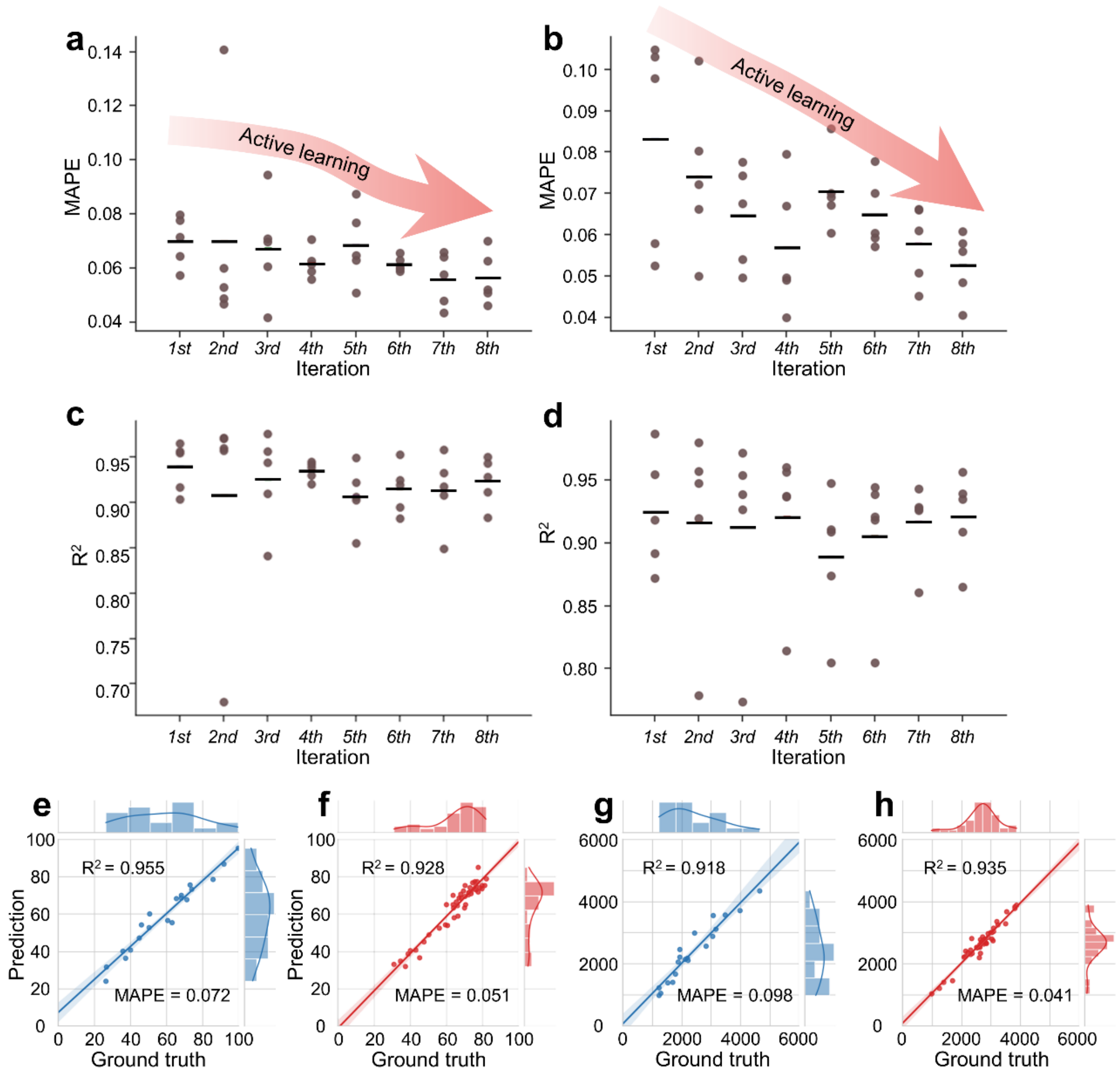


**Supplementary Figure 25: Model performance of 3D-CNNs**

(**a**, **b**) MAPE in iteration (Black short lines in the figure depicts the mean values, $n$ = 5): Panels (**a**) and (**b**) illustrate the MAPE for yield strength (Y) and elastic modulus (E) predictions respectively, of the architected materials. The DANTE indicate the MAPE between Finite Element (FE) simulation results and the model predictions. Five statistically independent (orthogonal) models are utilized for these predictions, demonstrating a rapid decline in MAPE, reminiscent of a natural learning curve. (**c**, **d**) $R^2$ in iteration (Black short lines in the figure depicts the mean values, $n$ = 5): Panels (**c**) and (**d**) present the $R^2$ values for Y and E, respectively. The dots depict the $R^2$ values comparing FE simulations to predictions. (**e**, **f**) Regression plots for Y predictions: Panels (**e**) and (**f**) display regression plots for the initial and final rounds of iteration, respectively, focusing on the prediction of Y. (**g**, **h**) Regression plots for E predictions: Panels (**g**) and (**h**) showcase regression plots for the first and last rounds of iteration, respectively, aimed at predicting the E. All $R^2$ and MAPE values are calculated using the validation dataset, providing a robust measure of model accuracy and learning efficiency of the self-driving virtual laboratories.

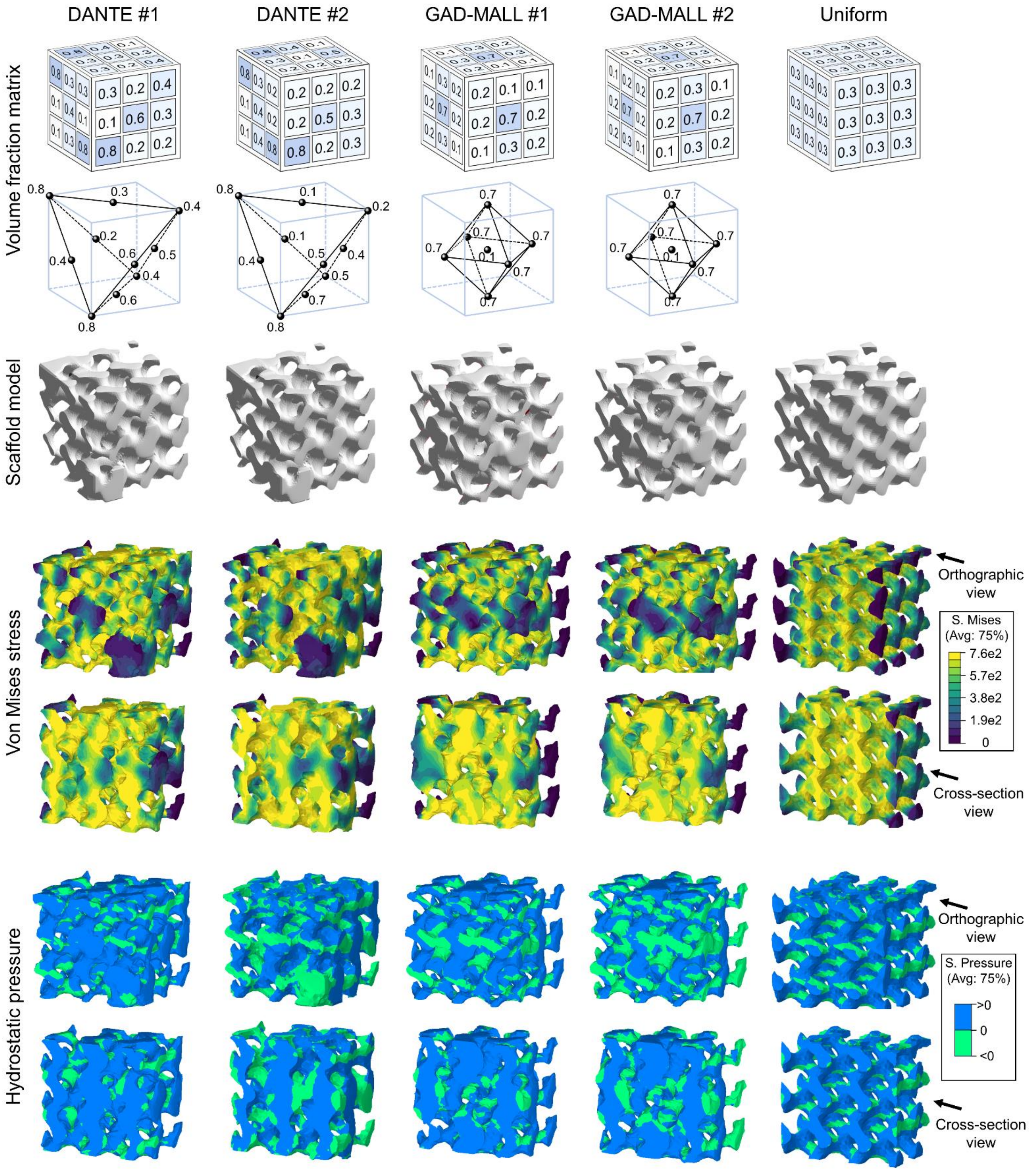


**Supplementary Figure 26: Analysis of architected materials**

Density matrices, scaffold models, Von Mises stress, and hydrostatic pressure of the architected materials labeled DANTE #1, DANTE #2, GAD-MALL #1, GAD-MALL #2, and Uniform. Density is defined as one minus the porosity.

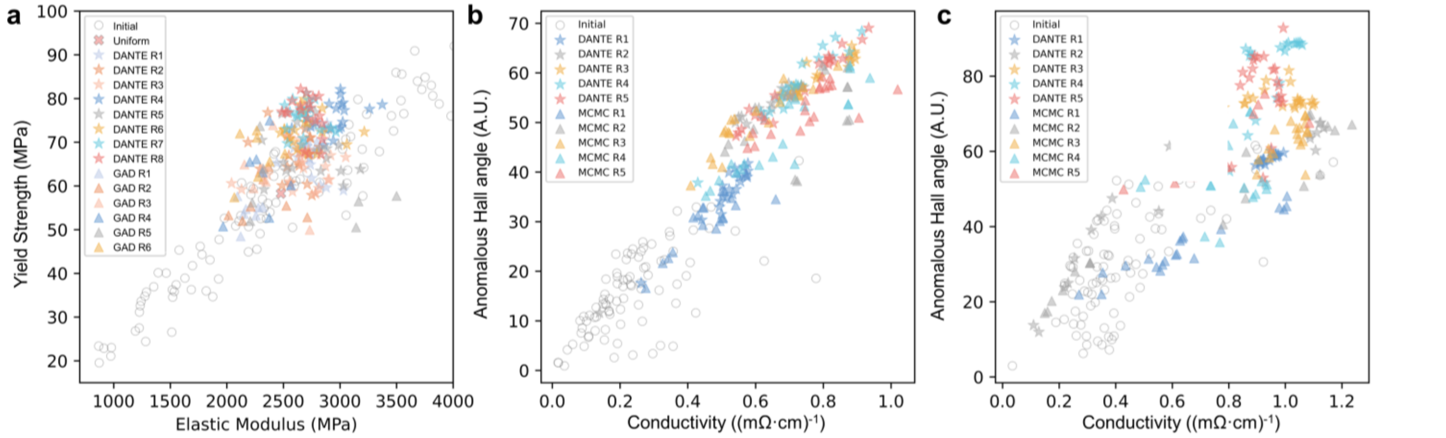


**Supplementary Figure 27: Results of autonomous virtual laboratories (AVL)-DANTE**

(**a**) Architected materials design (**b**, **c**) Compositionally complex alloy design: (**b**) fcc, (**c**) bcc. Initial data points are displayed using gray circles. The sampled data points of DANTE are displayed using five-pointed stars, and different colors denote different iterations. The sampled data points of SOTA methods are displayed using triangles. "DANTE R1" denotes the sampled data points of DANTE in the first iteration. The unit of AHC ($\sigma_{xy}$) is $(\mathrm{m\Omega\ cm})^{-1}$

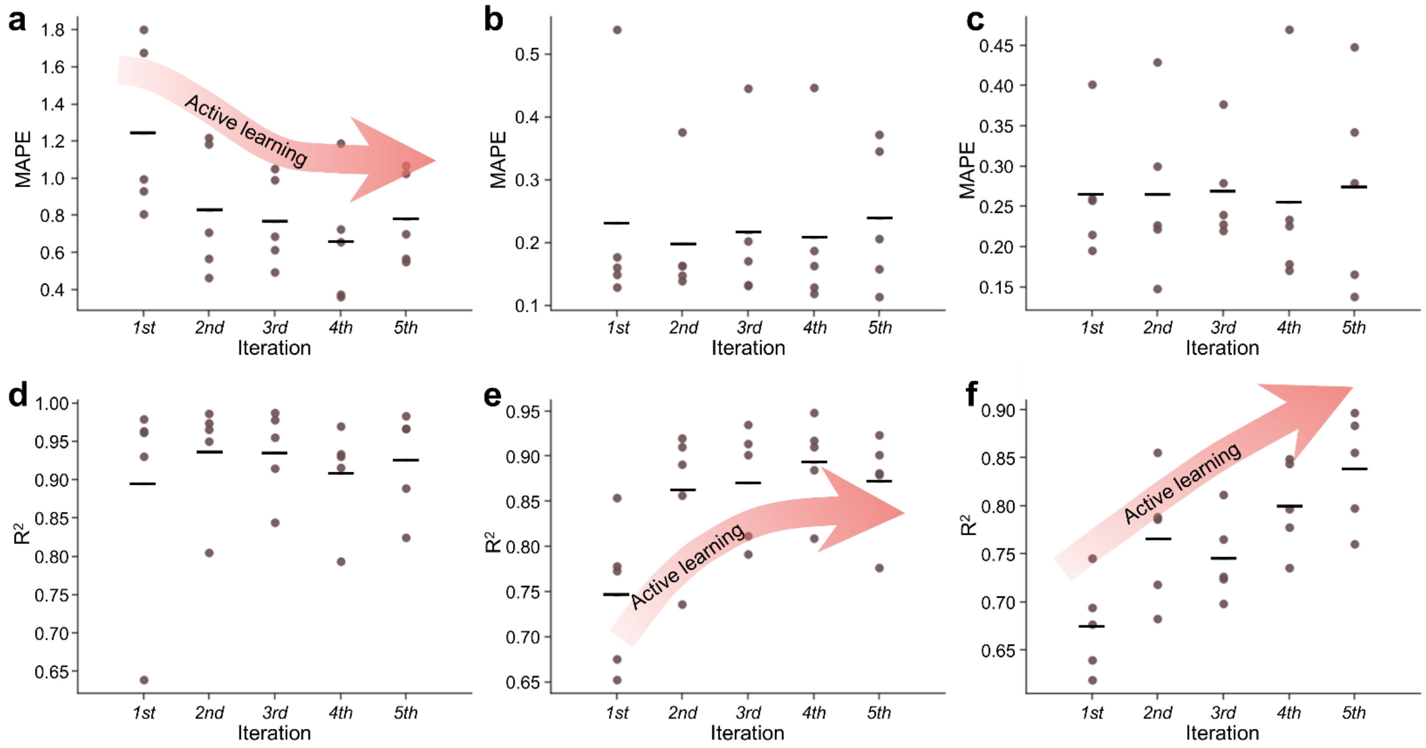


**Supplementary Figure 28: Model performance of 1D-CNNs utilizing DANTE for bcc CCA design**

(**a**-**c**) MAPE in iteration: Panels (**a**), (**b**), and (**c**) illustrate the MAPE for the formation energy, AHC, and AHA predictions respectively, of the CCAs. The dots indicate the MAPE between DFT results and the model predictions. Five statistically independent (orthogonal) models are utilized for these predictions, demonstrating a rapid decline in MAPE, reminiscent of a natural learning curve. (**d**-**f**) $R^2$ in iteration: Panels (**d**), (**e**) and (**f**) present the $R^2$ values for the formation energy, AHC, and AHA, respectively. The dots depict the $R^2$ values comparing DFT results to predictions. All $R^2$ and MAPE values are calculated using the validation dataset. Black short lines in the figure depicts the mean values, *n* = 5.

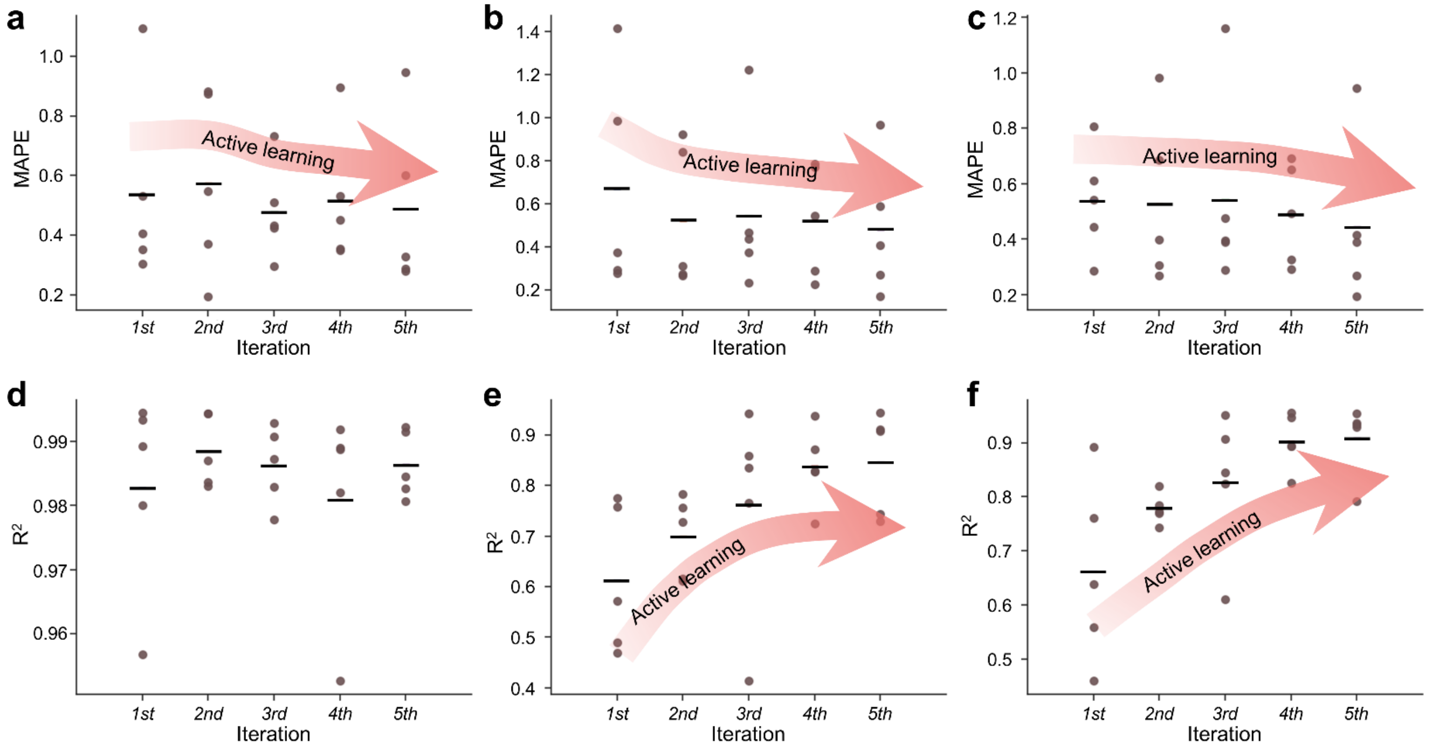


**Supplementary Figure 29: Model performance of 1D-CNNs utilizing DANTE for fcc CCA design**

(**a-c**) MAPE in iteration: Panels (**a**), (**b**), and (**c**) illustrate the MAPE for the formation energy, AHC, and AHA predictions respectively, of the CCAs. The dots indicate the MAPE between DFT results and the model predictions. Five statistically independent (orthogonal) models are utilized for these predictions, demonstrating a rapid decline in MAPE, reminiscent of a natural learning curve. (**d-f**) $R^2$ in iteration: Panels (**d**), (**e**) and (**f**) present the $R^2$ values for the formation energy, AHC, and AHA, respectively. The dots depict the $R^2$ values comparing DFT results to predictions. All $R^2$ and MAPE values are calculated using the validation dataset. Black short lines in the figure depicts the mean values, $n = 5$.

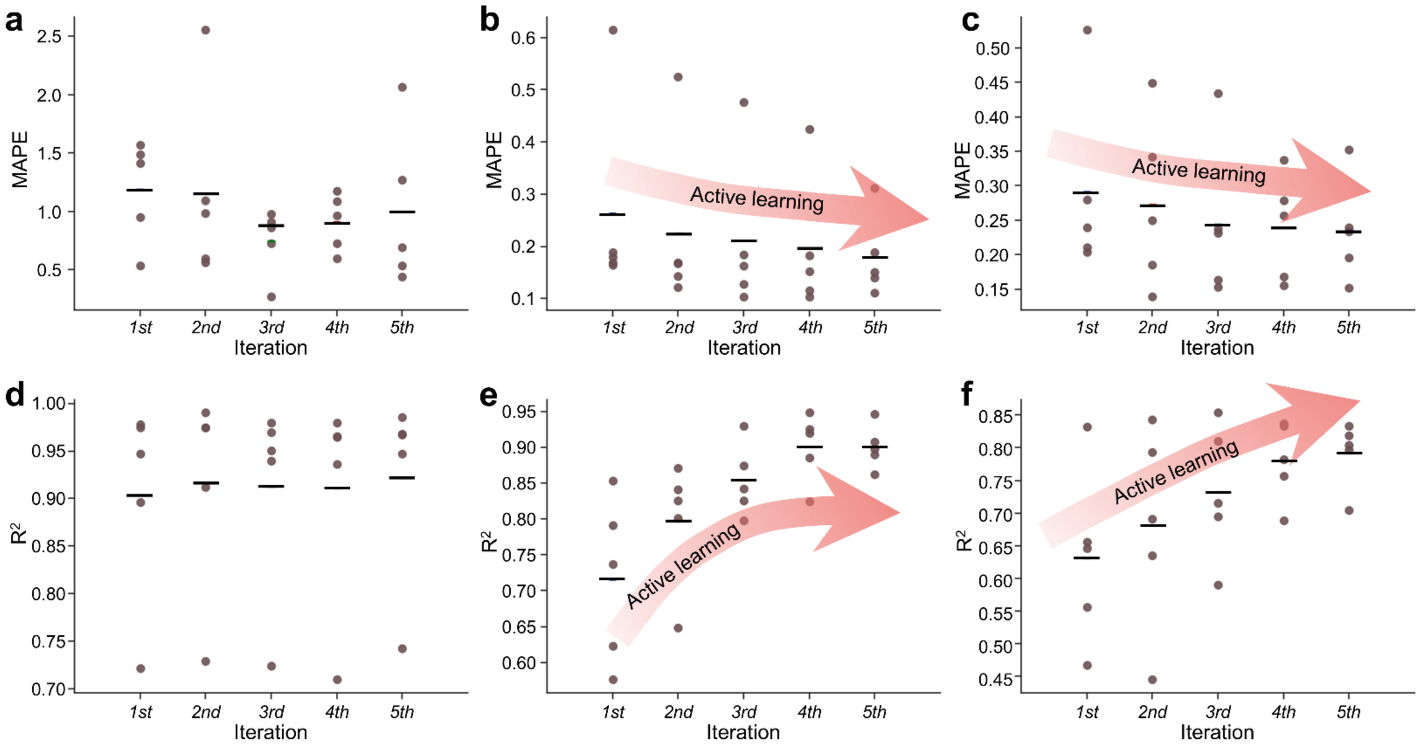


**Supplementary Figure 30: Model performance of 1D-CNNs utilizing MCMC for bcc CCA design**

(**a**-**c**) MAPE in iteration: Panels (**a**), (**b**), and (**c**) illustrate the MAPE for the formation energy, AHC, and AHA predictions respectively, of the CCAs. The dots indicate the MAPE between DFT results and the model predictions. Five statistically independent (orthogonal) models are utilized for these predictions, demonstrating a rapid decline in MAPE, reminiscent of a natural learning curve. (**d**-**f**) $R^2$ in iteration: Panels (**d**), (**e**) and (**f**) present the $R^2$ values for the formation energy, AHC, and AHA, respectively. The dots depict the $R^2$ values comparing DFT results to predictions. All $R^2$ and MAPE values are calculated using the validation dataset. Black short lines in the figure depicts the mean values, $n = 5$.

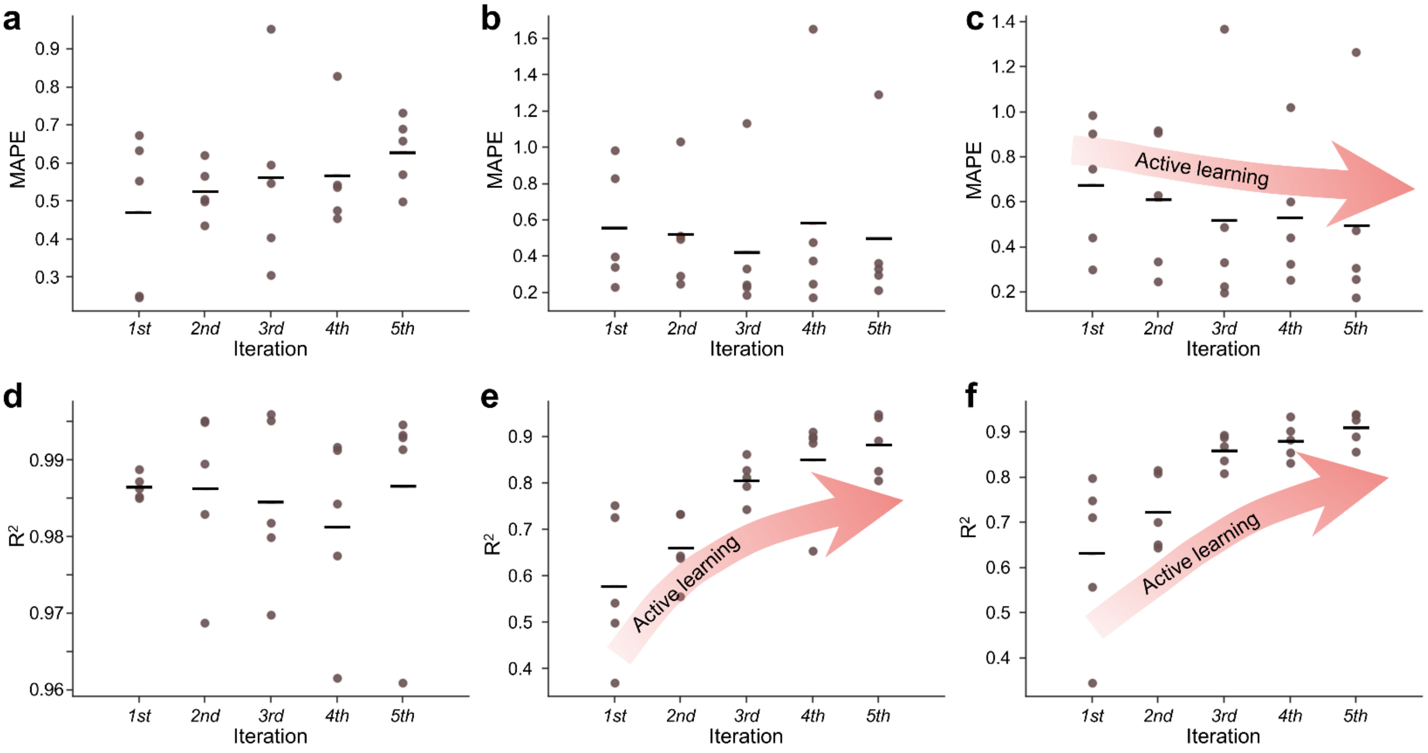


**Supplementary Figure 31: Model performance of 1D-CNNs utilizing MCMC for fcc CCA design**

(**a-c**) MAPE in iteration: Panels (**a**), (**b**), and (**c**) illustrate the MAPE for the formation energy, AHC, and AHA predictions respectively, of the CCAs. The dots indicate the MAPE between DFT results and the model predictions. Five statistically independent (orthogonal) models are utilized for these predictions, demonstrating a rapid decline in MAPE, reminiscent of a natural learning curve. (**d-f**) $R^2$ in iteration: Panels (**d**), (**e**) and (**f**) present the $R^2$ values for the formation energy, AHC, and AHA, respectively. The dots depict the $R^2$ values comparing DFT results to predictions. All $R^2$ and MAPE values are calculated using the validation dataset. Black short lines in the figure depicts the mean values, $n = 5$.

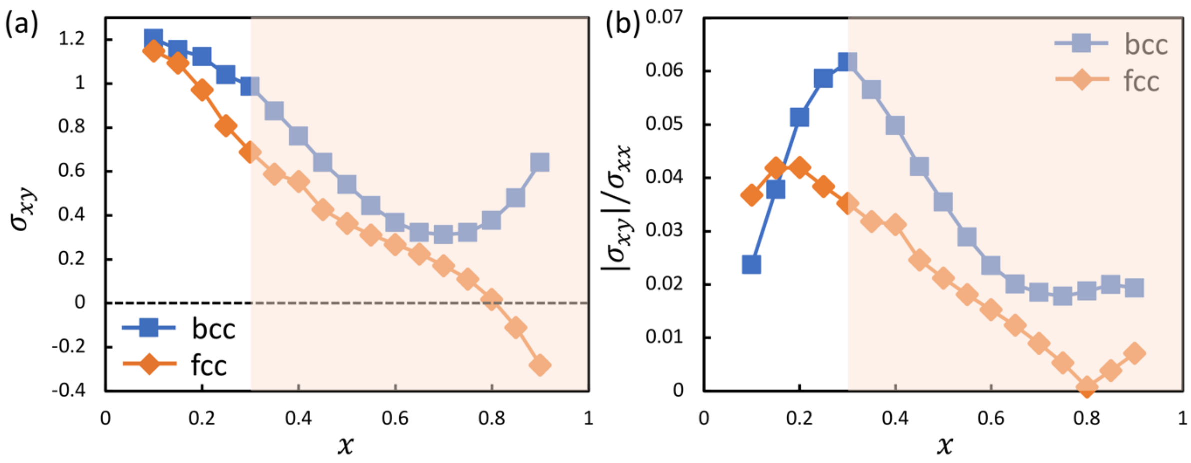


**Supplementary Figure 32: Calculated AHC and AHA of bcc and fcc $Fe_{1-x}Ir_x$.**

Calculated (a) AHC ($\sigma_{xy}$) and (b) AHA ($|\sigma_{xy}|/\sigma_{xx}$) of bcc and fcc $Fe_{1-x}Ir_x$. The shadow marks the critical composition from which the fcc phase is more thermodynamically stable based on the calculated formation energy.

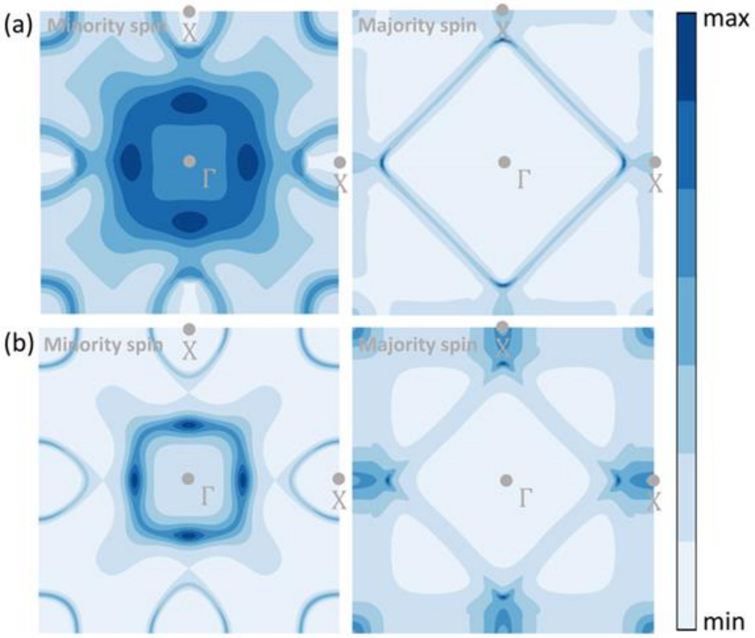


**Supplementary Figure 33: Bloch spectral functions ($E = E_{Fermi}$) in the (001) plane through Γ corresponding to minority and majority spin channels of fcc (a) $Fe_{43.5}Co_{18.5}Ni_{10}Al_{4.5}Zn_{9.5}Ir_{14}$ and (b) $Fe_{65}Ir_{35}$.**

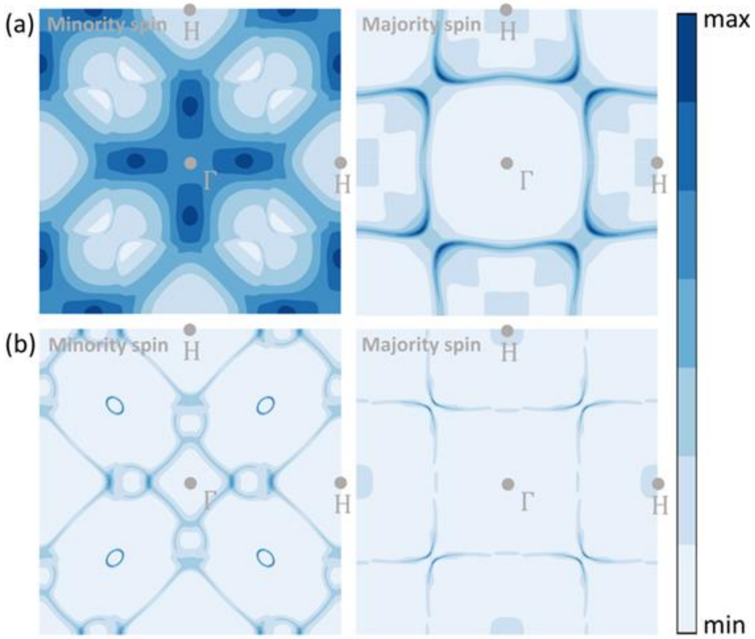


**Supplementary Figure 34: Bloch spectral functions ($E = E_{Fermi}$) in the (001) plane through Γ corresponding to minority and majority spin channels of bcc (a) $Fe_{61.5}Co_{0.5}Ni_{0.5}Si_{2.5}Zn_{19}Ir_{16}$ and (b) $Fe_{80}Ir_{20}$.**

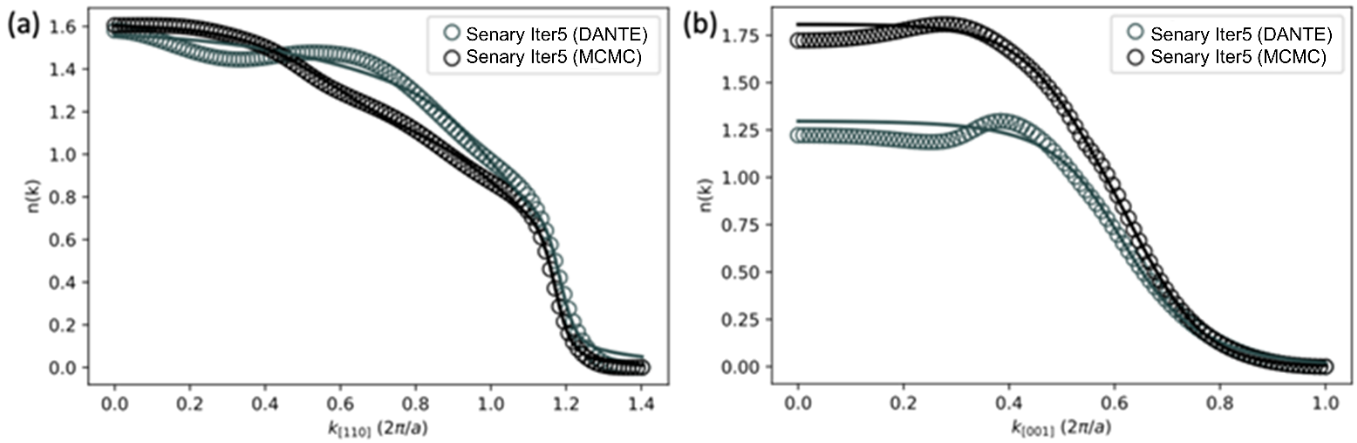


**Supplementary Figure 35: Fitting of n(k) for CCAs**

Fitting of n(k) for (**a**) fcc and (**b**) bcc CCAs predicted by DANTE and MCMC at the 5th iteration. The degrees of smearing corresponding to DANTE and MCMC in fcc CCAs are 0.24 and 0.15, respectively. The degrees of smearing corresponding to DANTE and MCMC in bcc CCAs are 0.16 and 0.13, respectively.

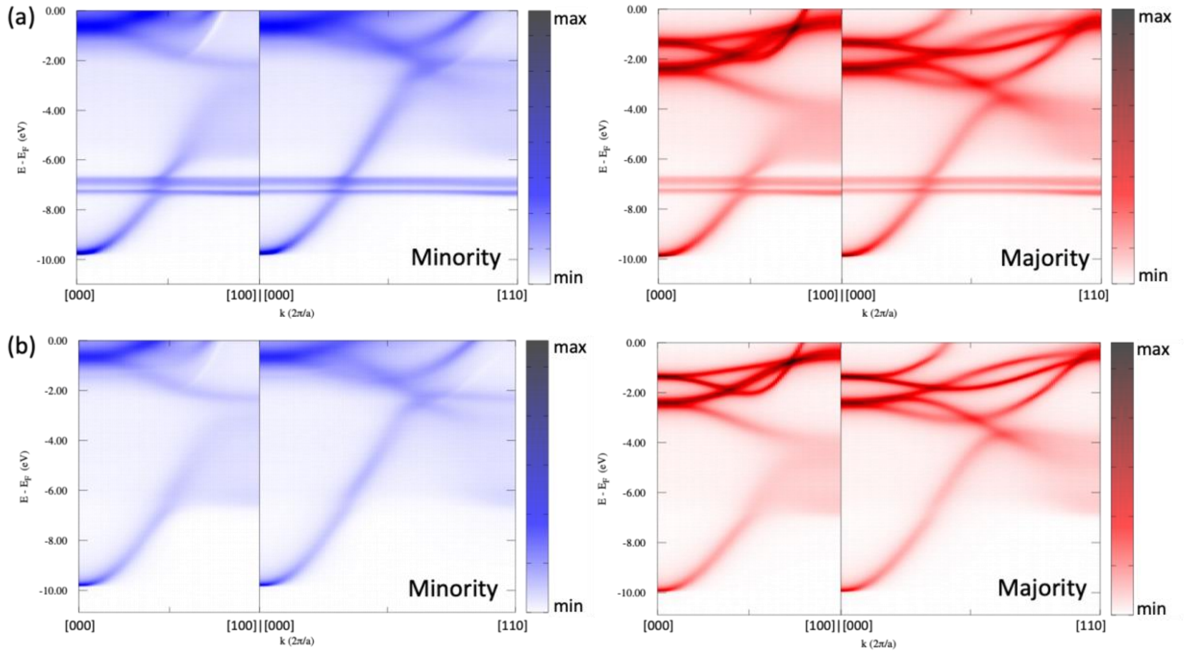


**Supplementary Figure 36: Band structures of fcc CCAs**

(**a**) Band structures of fcc $Fe_{43.5}Co_{19}Ni_{9.5}Ir_{14.5}Al_{5}Zn_{8.5}$ (predicted by DANTE at the 5th iteration) for minority (left panel) and majority (right panel) spin channels (**b**) Band structures of fcc $Fe_{47}Co_{17}Ni_{14.5}Ir_{7}Al_{5.5}Pt_{9}$ (predicted by MCMC at the 5th iteration) for minority (left panel) and majority (right panel) spin channels.

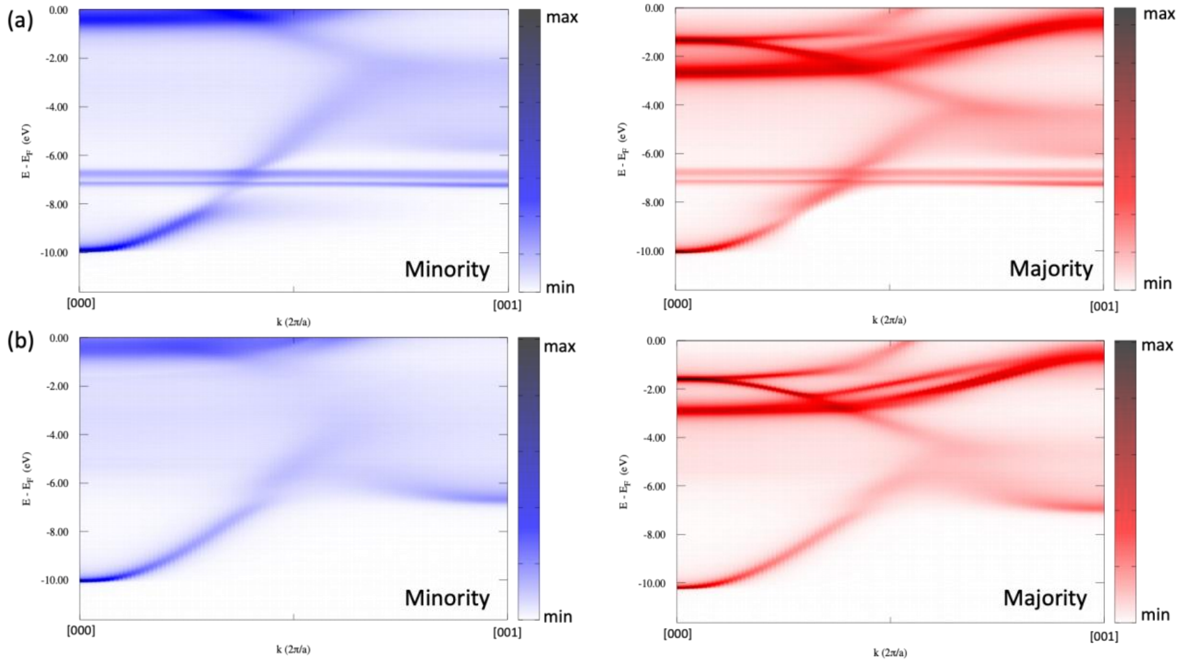


**Supplementary Figure 37: Band structures of bcc CCAs**

(**a**) Band structures of bcc $Fe_{63.5}Co_{0.5}Ni_{0.5}Ir_{18.5}Al_{9}Zn_{8}$ (predicted by DANTE at the 5th iteration) for minority (left panel) and majority (right panel) spin channels (**b**) Band structures of bcc $Fe_{58.5}Co_{1}Mg_{1.5}Ir_{5}Al_{5}Pt_{29}$ (predicted by MCMC at the 5th iteration) for minority (left panel) and majority (right panel) spin channels.

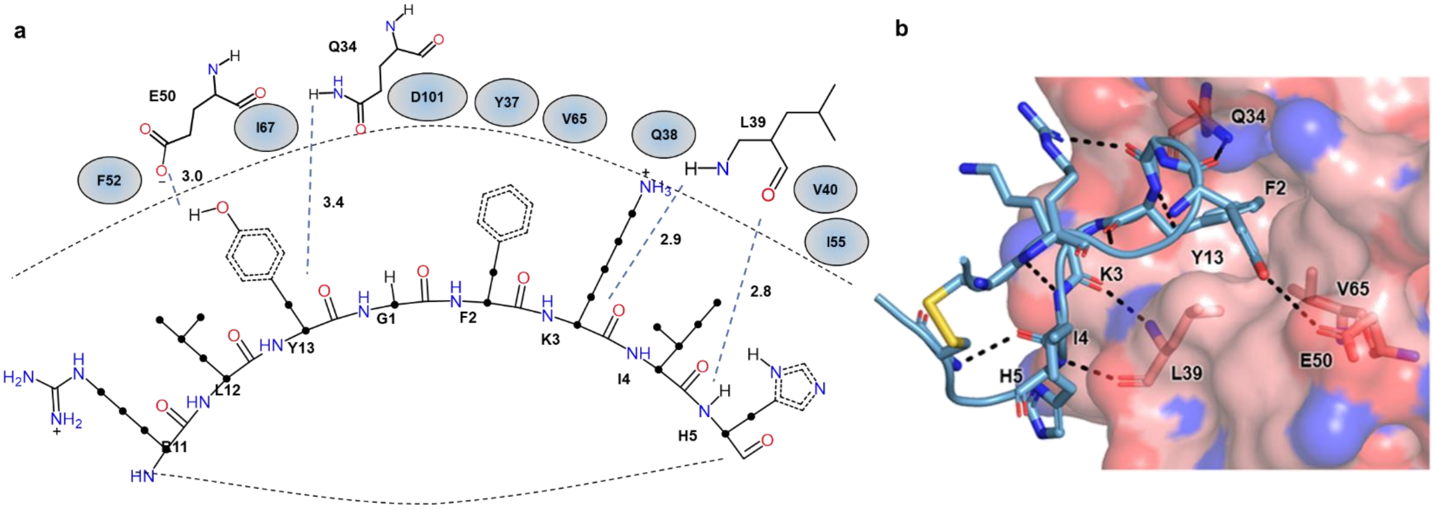


**Supplementary Figure 38: Interaction diagram and 3D conformation of the CK2α1−335/Pc cocrystals (PDBID: 4ib5)**

(**a**) Interaction diagram shows that Pc as a native cyclic peptide binder binds CK2α1−335 subunit A via the known CK2β-binding site at the outer surface of the N-terminal β-sheet. The binding site features a distinctive architecture where a hydrophobic cavity is situated adjacent to a solvent-accessible surface. (**b**) 3D conformation of cyclic peptide at the pocket shows that this cavity is encircled by key residues, including Y37, V65, V99, and A108. The Pc ligand plunges deeply into the hydrophobic cavity with a phenyl group of residue F2. Within the native cyclic peptide binder Pc, the hydrophilic amino acids Y13, K3, and H5 are pivotal, engaging in interactions with crucial CK2 residues including Q34, E50, and L39.

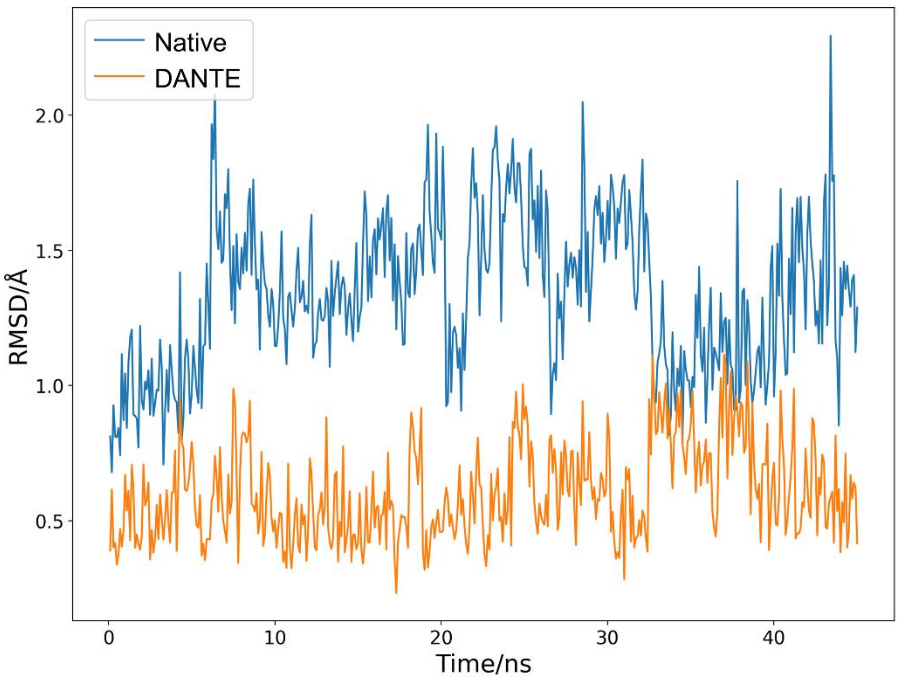


**Supplementary Figure 39: RMSD of backbone atoms of peptide in native and DANTE-designed complex (PDB ID: 4ib5)**

The mean RMSD for the native complex is 1.34 Å, compared to 0.60 Å for the DANTE-designed complex.

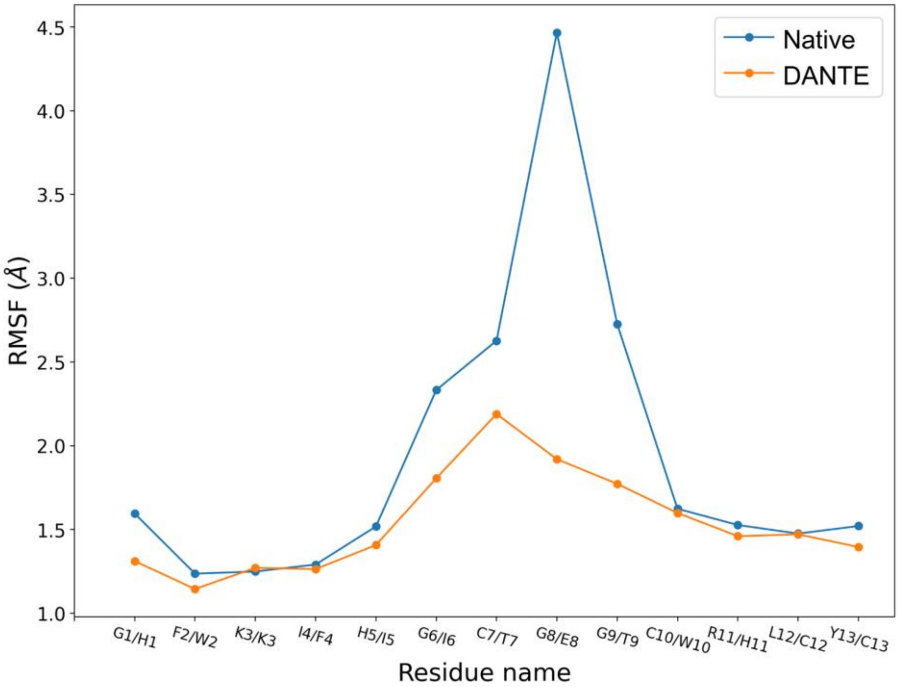


**Supplementary Figure 40: RMSF of c alpha atoms of native and DANTE-designed complex structures (PDB ID: 4ib5)**

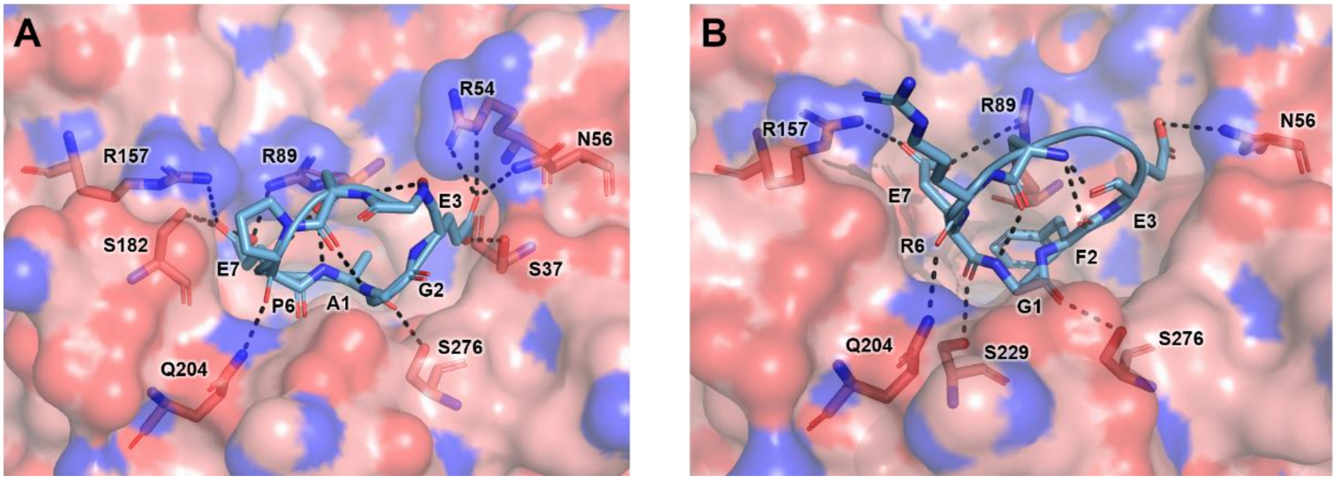


**Supplementary Figure 41: 3D interaction plot of complex with PDB ID: 7k2j**

Black dash shows the hydrogen bond. (A) Native complex with SC = 0.65, dSASA = 865Å². (B) DANTE-designed complex and numbering DANTE_3 with SC = 0.66, dSASA = 956Å². A benzene ring of F2 inserts into the cavity and increase the size of the interface.

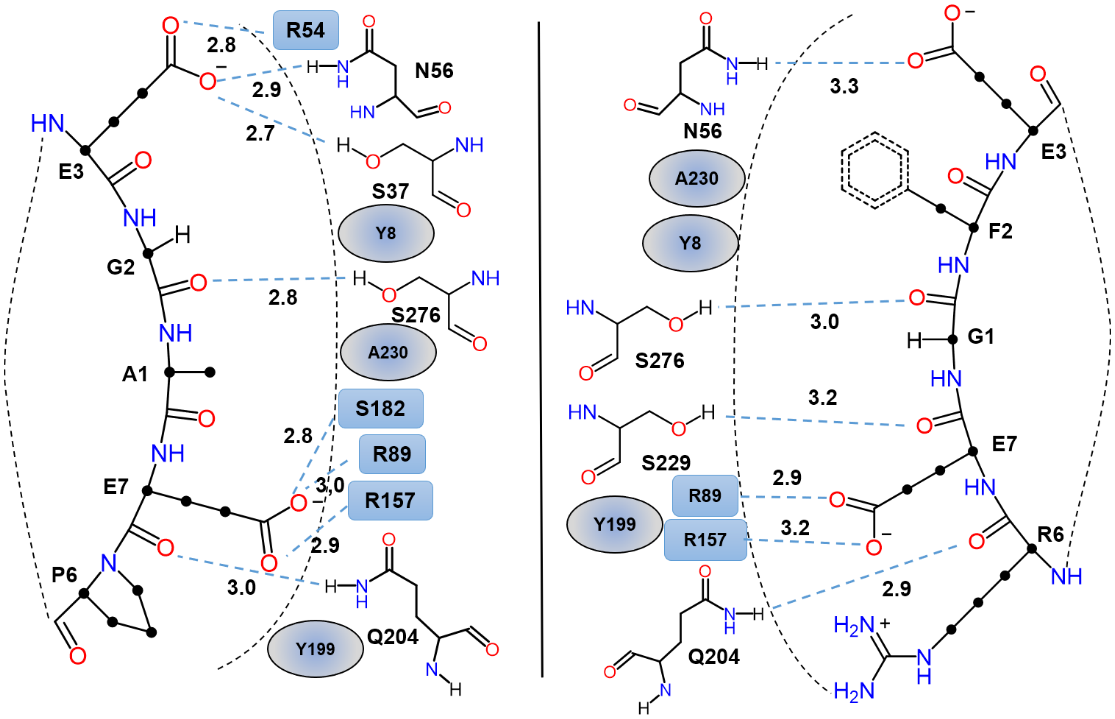


**Supplementary Figure 42: 2D interaction plot of complex with PDB ID: 7k2j**

Some interactions are not shown in the graph due to space limitations. The blue dash represents a hydrogen bond, while the oval-shaped residue indicates its involvement in hydrophobic interactions. Left: native 7k2j complex. Right: DANTE-designed complex.

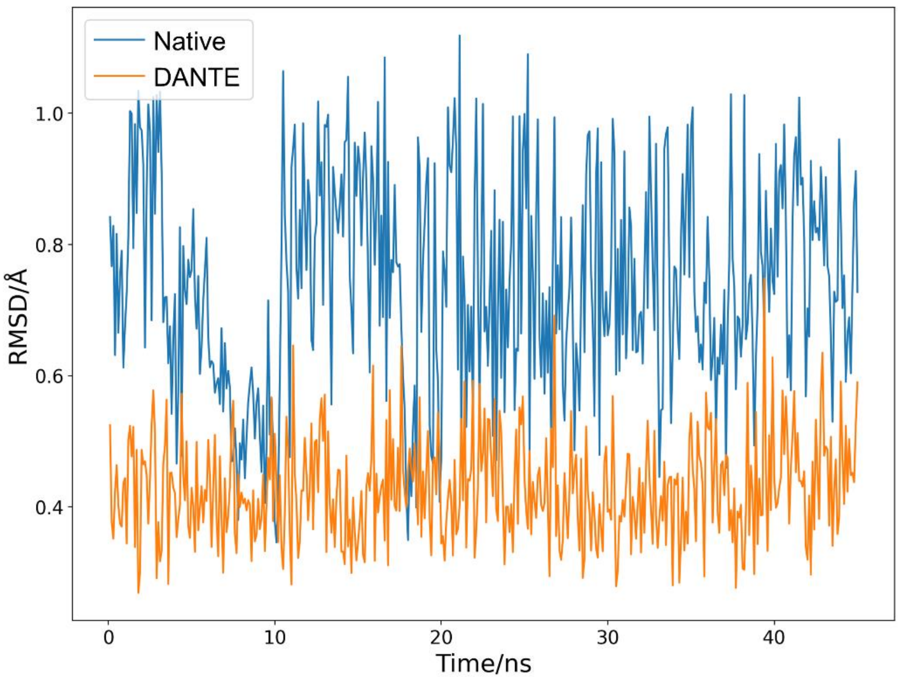


**Supplementary Figure 43: RMSD of backbone atoms of peptide in native and DANTE-designed complex (PDB ID: 7k2j)**

The mean RMSD for the native complex is 0.74 Å, compared to 0.43 Å for the DANTE-designed complex.

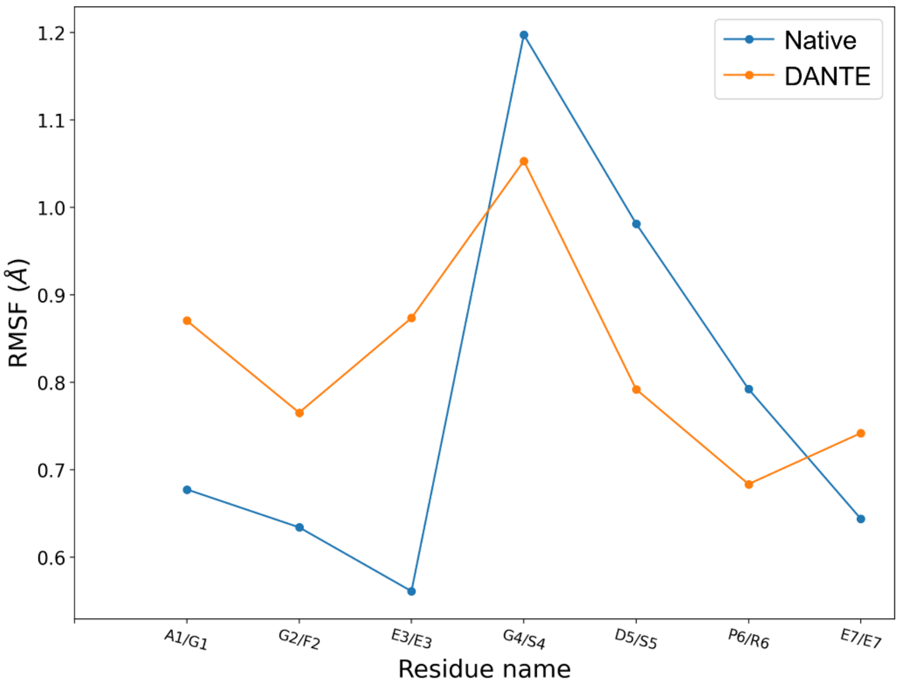


**Supplementary Figure 44: RMSF of c alpha atoms of native and DANTE-designed complex structures (PDB ID: 7k2j)**

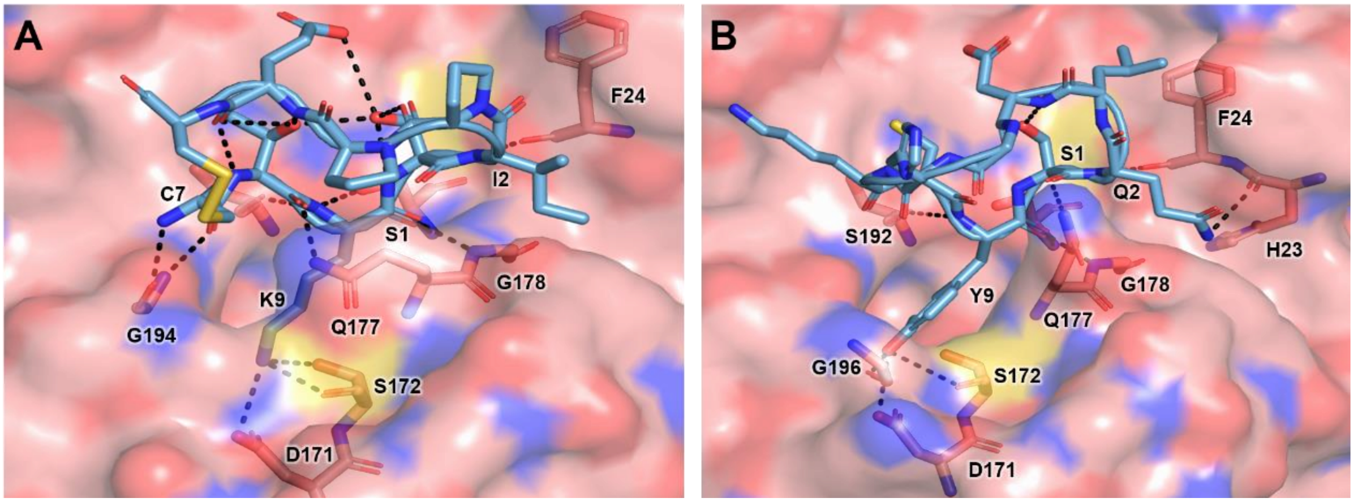


**Supplementary Figure 45: 3D interaction plot of complex with PDB ID: 1smf.**

Black dash shows the hydrogen bond. (A) Native complex with SC = 0.75, dSASA = 1005Å². (B) DANTE-designed complex and numbering DANTE_2 with SC = 0.76, dSASA = 1137Å². A Phenol ring of Y9 replacing the native alkyl chain of K9 inserts into the cavity and increase the size of the interface.

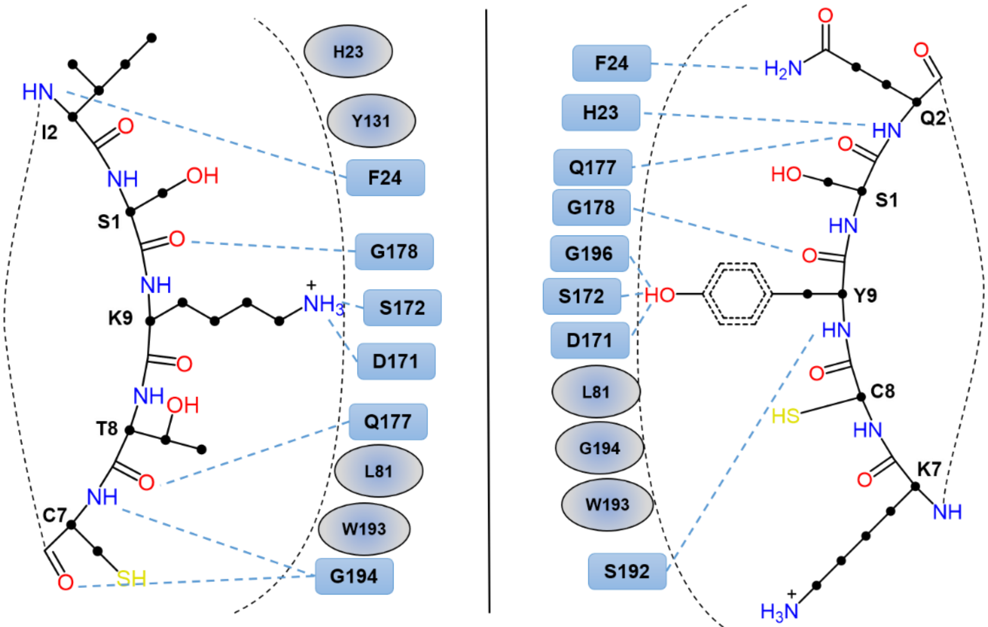


**Supplementary Figure 46: 2D interaction plot of complex with PDB ID: 1smf.**

Some interactions are not shown in the graph due to space limitations. The blue dash represents a hydrogen bond, while the oval-shaped residue indicates its involvement in hydrophobic interactions. Left: native 1smf complex. Right: DANTE-designed complex.

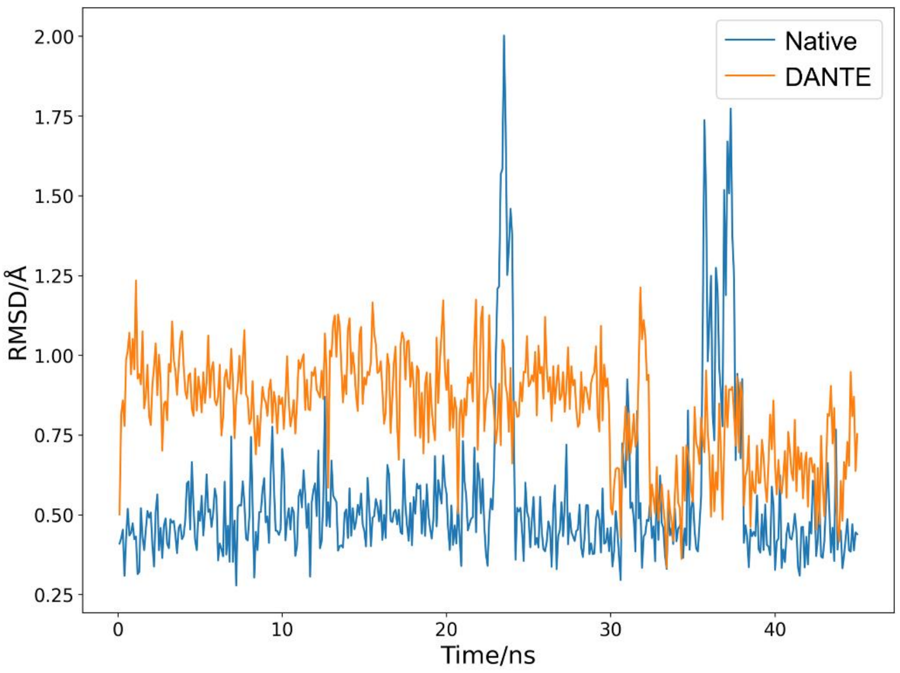


**Supplementary Figure 47: RMSD of backbone atoms of peptide in native and DANTE-designed complex (PDB ID: 1smf)**

The mean RMSD for the native complex is 0.54 Å, compared to 0.83 Å for the DANTE-designed complex.

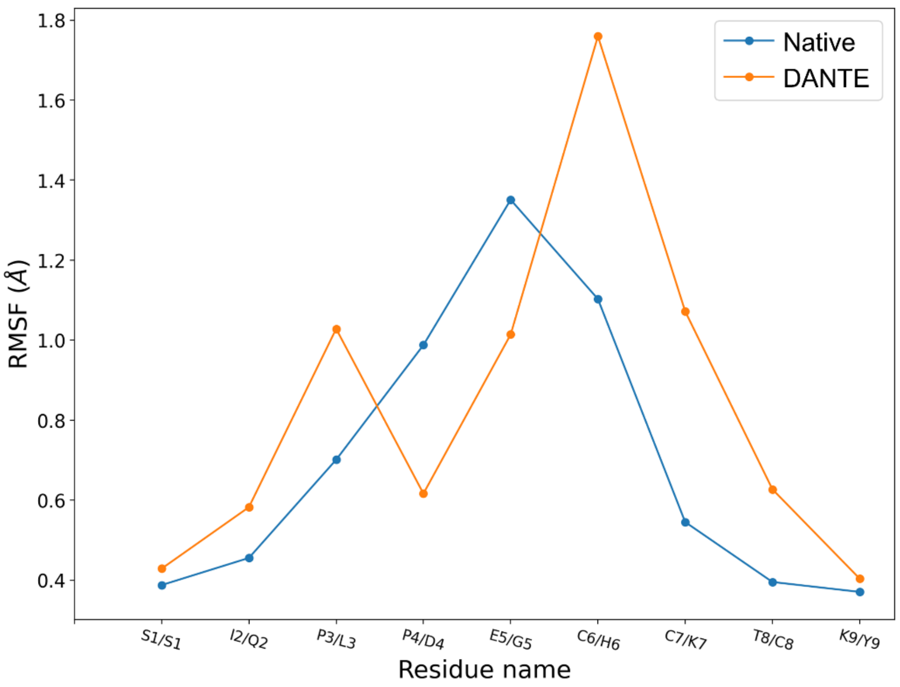


**Supplementary Figure 48 RMSF of c alpha atoms of native and DANTE-designed complex structures (PDB ID: 1smf)**

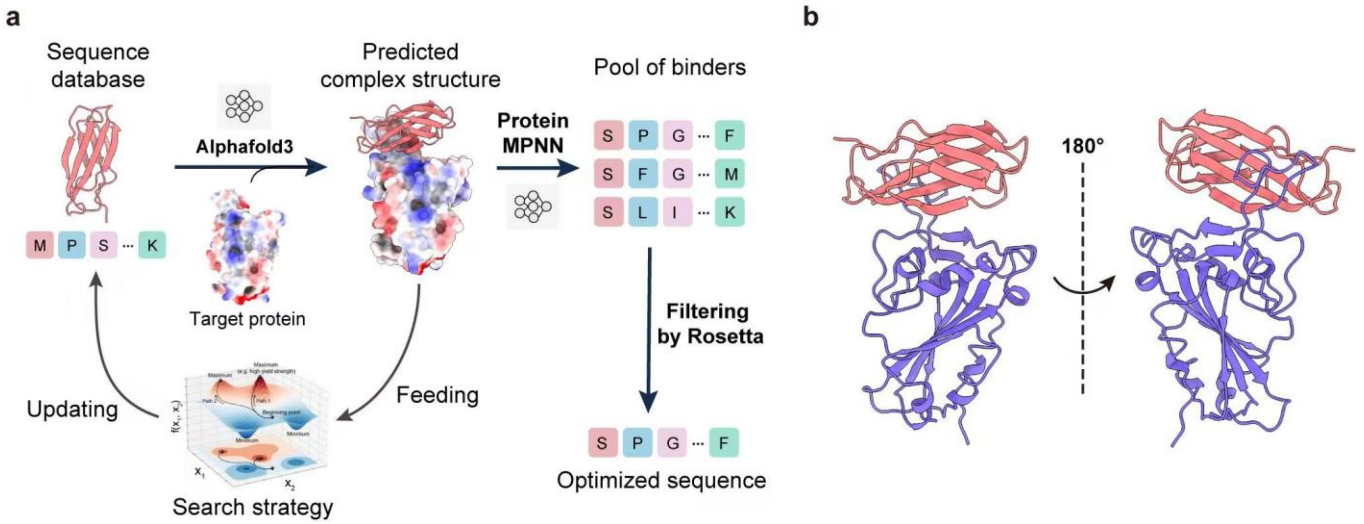


**Supplementary Figure 49: Pipeline of designing monobody binder to SARS-COVID-2 spike protein**

Search strategies are DANTE, MCMC and TuRBO5.

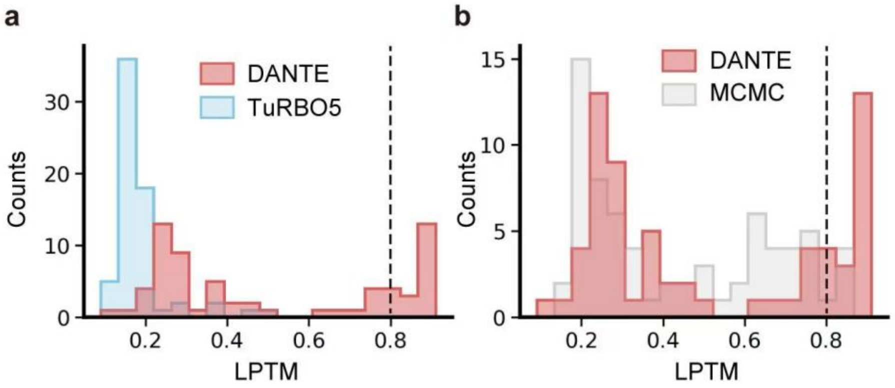


**Supplementary Figure 50: The ipTM distributions obtained from different methods**

The results reveal that DANTE notably outperforms TurBO5 and generates a greater number of high-quality designs compared to MCMC.

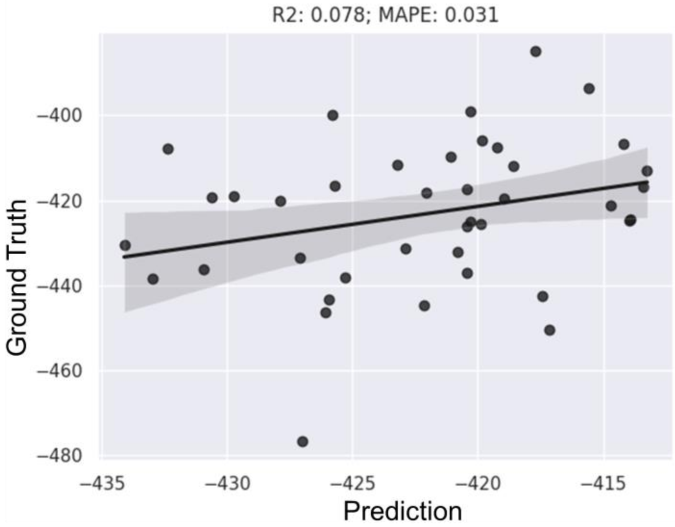


**Supplementary Figure 51: Evaluation of 1D-CNN Performance for Schwefel Function Prediction**

We conducted extensive testing on various 1D-CNN architectures to assess their performance in predicting the 100-dimensional Schwefel function. We found that 1D-CNNs struggle to accurately predict the Schwefel function, primarily due to their difficulty in learning the function's gradient.

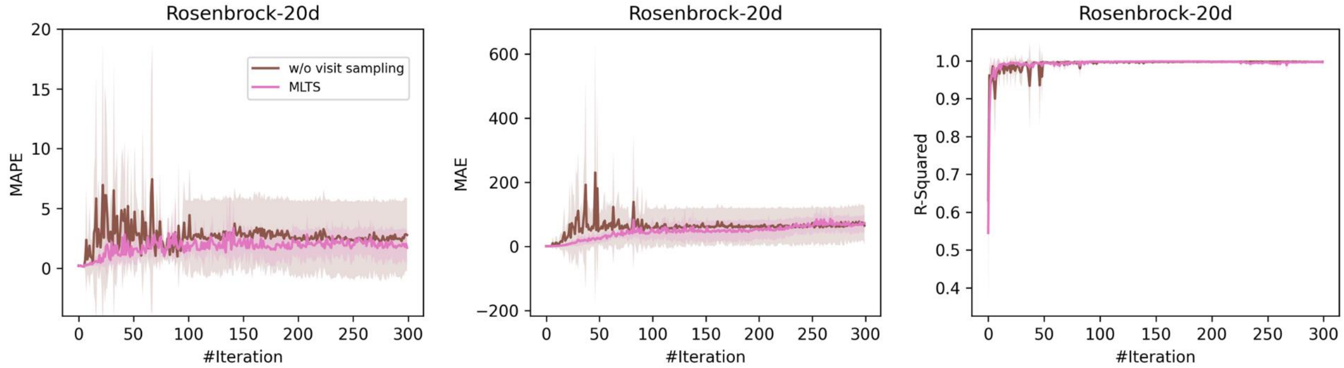


**Supplementary Figure 52: Surrogate model performance with and without top-visit sampling**

(pink and brown line, respectively). It can be observed that the loss without top-visit sampling is considerably higher and oscillates much more than the one with top-visit sampling. Data are presented as mean values ± SD, $n = 5$.

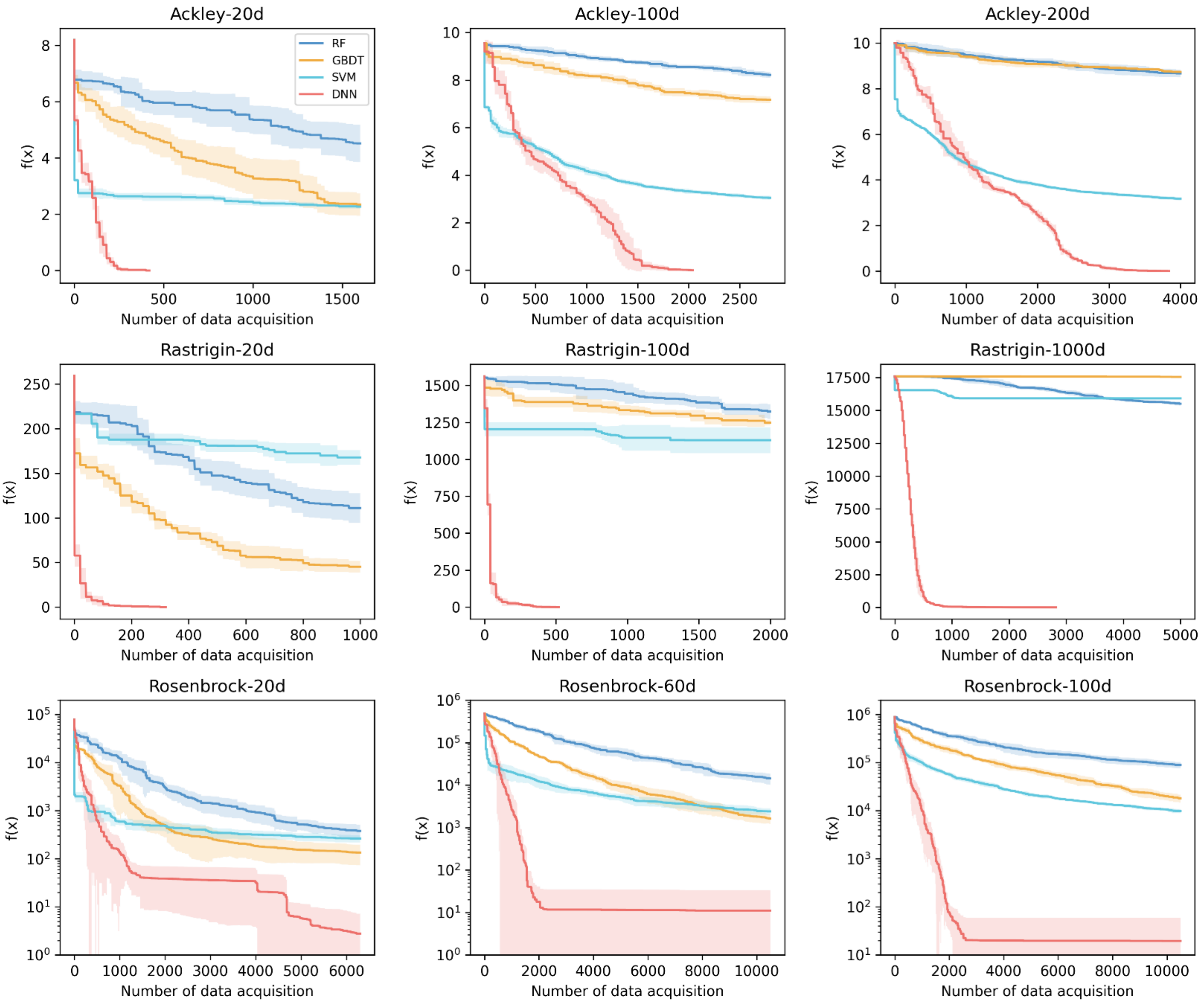


**Supplementary Figure 53: Evaluations on synthetic functions of DANTE equipped with various machine learning model**

We use different machine learning models as the surrogate model of DANTE and do the evaluations on Ackley, Rastrigin, and Rosenbrock functions. In this figure, Ackley-20d means the tests on 20-dimension Ackley function. The benchmark study shows that the convolutional neural network outperforms the other three machine learning models, e.g. GBDT (Gradient Boosting Decision Tree), RF (Random Forest), and SVM (Support Vector Machine). Data are presented as mean values ± SD, $n = 5$.

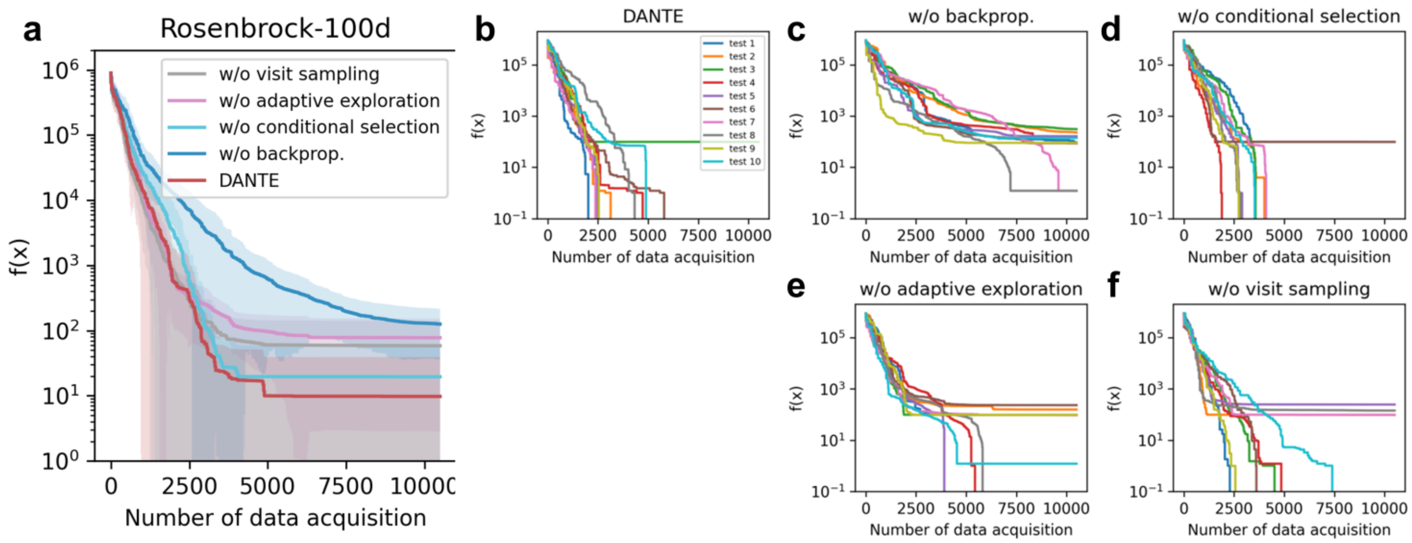


**Supplementary Figure 54: DANTE performance on Rosenbrock-100d using surrogate model**

(**a**) Ablation study on the individual mechanism. Data are presented as mean values ± SD, $n = 10$. (**b-e**) 10-time individual tests' results: (**b**) DANTE, (**c**) without local backpropagation, (**d**) without conditional selection (**e**) without adaptive exploration, (**f**) without top-visit sampling.

# Supplementary Tables

**Supplementary Table 1: Setup for synthetic functions**

The asterisk (*) represents the global minimum of the function, definition of these functions can be found here: https://www.sfu.ca/~ssurjano/optimization.html

| Feature | Ackley | Rastrigin | Rosenbrock | Schwefel | Griewank | Michalewicz | Levy |
|---|---|---|---|---|---|---|---|
| **Upper bound** | 5 | 5 | 5 | 500 | 600 | $\pi$ | 10 |
| **Lower bound** | -5 | -5 | -5 | -500 | -600 | 0 | -10 |
| **Step size** | 0.1 | 0.1 | 0.1 | 1 | 1 | 0.0001 | 0.1 |
| $f(x^*)$ | 0 | 0 | 0 | 0 | 0 | -9.66015 at $d = 10$ | 0 |
| $x^*$ | (0, …, 0) | (0, …, 0) | (1, …, 1) | (420.9687, …, 420.9687) | (0, …, 0) | (2.20, 1.57, …) | (1, …, 1) |

**Supplementary Table 2: Optimized reconstruction parameters by different methods**

| Methods | Probe-forming semi-angle | Defocus (Å) | Beam energy (kV) | Max iteration | Update step size | Identical slices iteration | Slice thickness (Å) | Number of slices | NMSE |
|---|---|---|---|---|---|---|---|---|---|
| **Expert** | 20 | 100 | 200 | 256 | 0.175 | 256 | 30.79423 | 6 | 0.2967 |
| **DOST** | 19.7 | 95 | 220 | 20 | 0.28 | 8 | 20.4 | 10 | 0.2909 |
| **TuRBO5** | 19.6 | 129 | 238 | 20 | 0.11 | 1 | 6.2 | 46 | 0.2914 |
| **BO** | 20.8 | 112 | 203 | 17 | 0.33 | 123 | 24.8 | 8 | 0.3145 |

**Supplementary Table 3: The convergence table of the benchmark functions.**

This table shows the max dimension at which DANTE converge to global optimum in our tests. "None" means not be tested. All results can be found at the GitHub, Source Data file folder. Schwefel and Michalewicz functions are not be tested using surrogate model since we find their gradient are hard to learn using neural networks.

| Objective | Ackley | Rastrigin | Rosenbrock | Griewank | Schwefel | Michalewicz | Levy |
|---|---|---|---|---|---|---|---|
| Exact function | 5000 | 5000 | 1000 | 5000 | 5000 | 500 | 5000 |
| Surrogate model | 1500 | 2000 | 200 | 500 | None | None | None |

**Supplementary Table 4: Detailed parameters and their bounds for electron ptychography reconstruction optimization**

| | Probe-forming semi-angle | Defocus (Å) | Beam energy (kV) | Max iteration | Update step size | Identical slices iteration | Slice thickness (Å) | Number of slices |
|---|---|---|---|---|---|---|---|---|
| **Upper bound** | 30 | 200 | 300 | 20 | 1 | 500 | 50 | 100 |
| **Lower bound** | 1 | 1 | 1 | 1 | 0.01 | 1 | 1 | 1 |

| | | | | | | | | |
|---|---|---|---|---|---|---|---|---|
| **Step size** | 0.1 | 1 | 1 | 1 | 0.01 | 1 | 0.1 | 1 |

**Supplementary Table 5: Hyperparameters for GBDT and RF models**

| Model | Objective | Evaluation metric | Learning rate | Feature fraction | Bagging fraction | Bagging frequency | Maximum depth | Number of leaves | Maximum number of bins | Number of iterations |
|---|---|---|---|---|---|---|---|---|---|---|
| **GBDT** | Regression | L2 for Ackley and Rosenbrock; MAPE for Rastrigin | 0.1 | 0.8 | 0.8 | 50 | 16 | 127 | 256 | 1000 |
| **RF** | Regression | | 0.1 | 0.8 | 0.8 | 50 | 16 | 127 | 256 | 1000 |

**Supplementary Table 6: Hyperparameters settings for all optimization algorithms in benchmarks**

In this table, the asterisk (*) denotes the samples with the highest prediction values. The double asterisk (**) represents the randomly chosen samples. The triple asterisk (***) means the most frequently visited nodes. These hyperparameters are fine-tuned such that the methods can be performed within 1 day on current hardware settings

| Algorithm | Objective | #Samples per iteration | Hyperparameters settings |
|---|---|---|---|
| **DANTE** | Exact function | 1 | The exploration weight is defined as $c_0 \times \max(1000/(f(x)+0.1))$, where the $c_0$ is set at 0.01 for Ackley, Rastrigin, Schwefel, and Griewank functions, and 0.1 for Rosenbrock and Michalewicz functions. Two types of expansion actions are possible: a stochastic move and a deterministic move (e.g., an adjustment of ± 0.1 for Ackley), each with an equal probability of 50%. |
| | Surrogate model | 20 (15* + 2** + 3***) | The exploration weight is defined as $c_0 \times \max(F(x))$, where the $c_0$ is set at 0.1 for Rosenbrock and 0.02 for other functions. In each iteration, the process involves 200 rollouts for Ackley and Rastrigin functions, and 100 rollouts for other functions. In each rollout, two expansion action categories are considered: stochastic moves, with a likelihood of 2/3, and deterministic moves (± 0.1), accounting for the remaining 1/3. Within the stochastic category, three probable variations are identified: altering a single variable in $x$, modifying $d/5$ variables in $x$, and adjusting $d/10$ variables in $x$. |
| **DANTE-Greedy** | Exact function | 1 | Similar to DANTE, yet without backpropagation (i.e., exploration weight equals 0) |
| | Surrogate model | 20 (20*) | |
| **DANTE-eGreedy** | Exact function | 1 | Similar to DANTE-Greedy, yet introduces an epsilon parameter to increase the possibility to choose random nodes, set to 0.2. |
| | Surrogate model | 20 (16* + 4**) | |
| **TuRBO5** | Surrogate model | 1 | The configuration includes five independent trust regions, with all other settings adhering to the defaults specified in the reference implementation (https://github.com/uber-research/TuRBO). |
| **LAMCTS** | Surrogate model | 1 | Default settings in the reference implementation (https://github.com/facebookresearch/LaMCTS). |
| **CMA-ES** | Exact function | 1 | The initial standard deviation is set to 1, and the rest parameters default in Scipy. |
| | Surrogate model | 20 (16* + 4**) | The initial standard deviation is set to 0.5, the maximum iteration is set to 10 for time consideration. The rest parameters are default in Scipy. |

| | | | |
|---|---|---|---|
| **Diff-Evo** | Exact function | 1 | Default settings in Scipy. |
| | Surrogate model | 20<br>(16* + 4**) | The maximum iteration is set to 1, and popsize is defined as MAX(1, 100/$d$). The rest parameters are default in Scipy. |
| **DA** | Exact function | 1 | Default settings in Scipy. |
| | Surrogate model | 20<br>(16* + 4**) | The initial temperature is set to 0.05. The maximum number of function calls is set to 200 for Ackley and Rastrigin, and 100 for other functions. The rest parameters are default in Scipy. |
| **Shiwa** | Exact function | 1 | The budget is set to 20, other settings are default as in Nevergrad (https://github.com/facebookresearch/nevergrad). |
| | Surrogate model | 20<br>(16* + 4**) | |
| **MCMC** | Exact function | 1 | Similar to DANTE-Greedy, yet with only one child node in each rollout. |
| | Surrogate model | 20<br>(20*) | |
| **DOO** | Exact function | 1 | Default settings in the reference implementation (https://github.com/beomjoonkim/voot). |
| | Surrogate model | 20 | |
| **SOO** | Exact function | 1 | |
| | Surrogate model | 20 | |
| **VOO** | Exact function | 1 | |
| | Surrogate model | 20 | |

**Supplementary Table 7: Density matrices and corresponding mechanical properties of architected materials.**

The porosity matrix is equal to 1 minus the density matrix. # Iteration 0 means initial data. E means elastic modulus. Y means yield strength.

| Iteration | E (MPa) | Y(MPa) | 1 | 2 | 3 | 4 | 5 | 6 | 7 | 8 | 9 | 10 | 11 | 12 | 13 | 14 | 15 | 16 | 17 | 18 | 19 | 20 | 21 | 22 | 23 | 24 | 25 | 26 | 27 |
|---|---|---|---|---|---|---|---|---|---|---|---|---|---|---|---|---|---|---|---|---|---|---|---|---|---|---|---|---|---|
| 0 | 1283 | 24.4 | 0.1 | 0.1 | 0.1 | 0.6 | 0.1 | 0.6 | 0.4 | 0.1 | 0.4 | 0.1 | 0.1 | 0.1 | 0.4 | 0.1 | 0.4 | 0.6 | 0.1 | 0.6 | 0.6 | 0.1 | 0.6 | 0.1 | 0.1 | 0.1 | 0.4 | 0.1 | 0.4 |
| 0 | 2710 | 63.1 | 0.1 | 0.2 | 0.1 | 0.5 | 0.6 | 0.5 | 0.2 | 0.6 | 0.2 | 0.1 | 0.2 | 0.1 | 0.2 | 0.2 | 0.2 | 0.5 | 0.2 | 0.5 | 0.5 | 0.6 | 0.5 | 0.1 | 0.6 | 0.1 | 0.2 | 0.2 | 0.2 |
| 0 | 2597 | 60.0 | 0.1 | 0.2 | 0.6 | 0.1 | 0.2 | 0.6 | 0.1 | 0.2 | 0.6 | 0.3 | 0.3 | 0.3 | 0.6 | 0.6 | 0.6 | 0.3 | 0.3 | 0.3 | 0.1 | 0.2 | 0.6 | 0.1 | 0.2 | 0.6 | 0.1 | 0.2 | 0.6 |
| 0 | 2581 | 50.5 | 0.1 | 0.3 | 0.1 | 0.3 | 0.2 | 0.3 | 0.6 | 0.2 | 0.6 | 0.1 | 0.3 | 0.1 | 0.6 | 0.3 | 0.6 | 0.3 | 0.3 | 0.3 | 0.3 | 0.2 | 0.3 | 0.1 | 0.2 | 0.1 | 0.6 | 0.3 | 0.6 |
| 0 | 2192 | 50.7 | 0.1 | 0.3 | 0.3 | 0.1 | 0.3 | 0.3 | 0.1 | 0.3 | 0.3 | 0.4 | 0.4 | 0.4 | 0.3 | 0.3 | 0.3 | 0.4 | 0.4 | 0.4 | 0.1 | 0.3 | 0.3 | 0.1 | 0.3 | 0.3 | 0.1 | 0.3 | 0.3 |
| 0 | 3808 | 83.0 | 0.1 | 0.3 | 0.5 | 0.1 | 0.3 | 0.5 | 0.1 | 0.3 | 0.5 | 0.5 | 0.5 | 0.5 | 0.6 | 0.6 | 0.6 | 0.5 | 0.5 | 0.5 | 0.1 | 0.3 | 0.5 | 0.1 | 0.3 | 0.5 | 0.1 | 0.3 | 0.5 |
| 0 | 1226 | 27.5 | 0.1 | 0.4 | 0.1 | 0.1 | 0.4 | 0.1 | 0.1 | 0.4 | 0.1 | 0.1 | 0.1 | 0.1 | 0.4 | 0.4 | 0.4 | 0.1 | 0.1 | 0.1 | 0.1 | 0.4 | 0.1 | 0.1 | 0.4 | 0.1 | 0.1 | 0.4 | 0.1 |
| 0 | 3495 | 85.9 | 0.1 | 0.4 | 0.1 | 0.4 | 0.6 | 0.4 | 0.1 | 0.4 | 0.1 | 0.4 | 0.6 | 0.4 | 0.6 | 0.2 | 0.6 | 0.4 | 0.6 | 0.4 | 0.1 | 0.4 | 0.1 | 0.4 | 0.6 | 0.4 | 0.1 | 0.4 | 0.1 |
| 0 | 2124 | 53.2 | 0.1 | 0.4 | 0.2 | 0.4 | 0.2 | 0.4 | 0.2 | 0.4 | 0.1 | 0.4 | 0.2 | 0.4 | 0.2 | 0.3 | 0.2 | 0.4 | 0.2 | 0.4 | 0.2 | 0.4 | 0.1 | 0.4 | 0.2 | 0.4 | 0.1 | 0.4 | 0.2 |
| 0 | 1686 | 36.3 | 0.1 | 0.4 | 0.3 | 0.1 | 0.4 | 0.3 | 0.1 | 0.4 | 0.3 | 0.2 | 0.2 | 0.2 | 0.3 | 0.3 | 0.3 | 0.2 | 0.2 | 0.2 | 0.1 | 0.4 | 0.3 | 0.1 | 0.4 | 0.3 | 0.1 | 0.4 | 0.3 |
| 0 | 1765 | 46.2 | 0.1 | 0.4 | 0.5 | 0.4 | 0.1 | 0.4 | 0.5 | 0.4 | 0.1 | 0.4 | 0.1 | 0.4 | 0.1 | 0.1 | 0.1 | 0.4 | 0.1 | 0.4 | 0.5 | 0.4 | 0.1 | 0.4 | 0.1 | 0.4 | 0.1 | 0.4 | 0.5 |
| 0 | 1513 | 26.5 | 0.1 | 0.4 | 0.6 | 0.1 | 0.4 | 0.6 | 0.1 | 0.4 | 0.6 | 0.1 | 0.1 | 0.1 | 0.3 | 0.3 | 0.3 | 0.1 | 0.1 | 0.1 | 0.1 | 0.4 | 0.6 | 0.1 | 0.4 | 0.6 | 0.1 | 0.4 | 0.6 |
| 0 | 1979 | 52.6 | 0.1 | 0.5 | 0.1 | 0.1 | 0.5 | 0.1 | 0.1 | 0.5 | 0.1 | 0.5 | 0.5 | 0.5 | 0.1 | 0.1 | 0.1 | 0.5 | 0.5 | 0.5 | 0.1 | 0.5 | 0.1 | 0.1 | 0.5 | 0.1 | 0.1 | 0.5 | 0.1 |
| 0 | 1228 | 31.1 | 0.1 | 0.5 | 0.2 | 0.1 | 0.5 | 0.2 | 0.1 | 0.5 | 0.2 | 0.2 | 0.2 | 0.2 | 0.2 | 0.2 | 0.2 | 0.2 | 0.2 | 0.2 | 0.1 | 0.5 | 0.2 | 0.1 | 0.5 | 0.2 | 0.1 | 0.5 | 0.2 |
| 0 | 3752 | 84.9 | 0.1 | 0.5 | 0.2 | 0.1 | 0.5 | 0.2 | 0.1 | 0.5 | 0.2 | 0.6 | 0.6 | 0.6 | 0.5 | 0.5 | 0.5 | 0.6 | 0.6 | 0.6 | 0.1 | 0.5 | 0.2 | 0.1 | 0.5 | 0.2 | 0.1 | 0.5 | 0.2 |
| 0 | 1876 | 34.7 | 0.1 | 0.5 | 0.5 | 0.1 | 0.5 | 0.5 | 0.1 | 0.5 | 0.5 | 0.2 | 0.2 | 0.2 | 0.3 | 0.3 | 0.3 | 0.2 | 0.2 | 0.2 | 0.1 | 0.5 | 0.5 | 0.1 | 0.5 | 0.5 | 0.1 | 0.5 | 0.5 |
| 0 | 2213 | 58.0 | 0.1 | 0.6 | 0.1 | 0.2 | 0.3 | 0.2 | 0.4 | 0.3 | 0.4 | 0.1 | 0.6 | 0.1 | 0.4 | 0.6 | 0.4 | 0.2 | 0.6 | 0.2 | 0.2 | 0.3 | 0.2 | 0.1 | 0.3 | 0.1 | 0.4 | 0.6 | 0.4 |
| 0 | 2434 | 56.2 | 0.1 | 0.6 | 0.5 | 0.1 | 0.6 | 0.5 | 0.1 | 0.6 | 0.5 | 0.3 | 0.3 | 0.3 | 0.4 | 0.4 | 0.4 | 0.3 | 0.3 | 0.3 | 0.1 | 0.6 | 0.5 | 0.1 | 0.6 | 0.5 | 0.1 | 0.6 | 0.5 |
| 0 | 1192 | 26.8 | 0.2 | 0.1 | 0.2 | 0.1 | 0.3 | 0.1 | 0.2 | 0.1 | 0.2 | 0.1 | 0.3 | 0.1 | 0.3 | 0.5 | 0.3 | 0.1 | 0.3 | 0.1 | 0.2 | 0.1 | 0.2 | 0.1 | 0.3 | 0.1 | 0.2 | 0.1 | 0.2 |
| 0 | 1235 | 32.6 | 0.2 | 0.1 | 0.2 | 0.1 | 0.4 | 0.1 | 0.2 | 0.1 | 0.2 | 0.1 | 0.4 | 0.1 | 0.4 | 0.2 | 0.4 | 0.1 | 0.4 | 0.1 | 0.2 | 0.1 | 0.2 | 0.1 | 0.4 | 0.1 | 0.2 | 0.1 | 0.2 |
| 0 | 872 | 19.5 | 0.2 | 0.1 | 0.2 | 0.2 | 0.1 | 0.2 | 0.2 | 0.1 | 0.2 | 0.2 | 0.1 | 0.2 | 0.2 | 0.1 | 0.2 | 0.2 | 0.1 | 0.2 | 0.2 | 0.1 | 0.2 | 0.2 | 0.1 | 0.2 | 0.2 | 0.1 | 0.2 |
| 0 | 3565 | 76.5 | 0.2 | 0.1 | 0.3 | 0.2 | 0.1 | 0.3 | 0.2 | 0.1 | 0.3 | 0.6 | 0.6 | 0.6 | 0.5 | 0.5 | 0.5 | 0.6 | 0.6 | 0.6 | 0.2 | 0.1 | 0.3 | 0.2 | 0.1 | 0.3 | 0.2 | 0.1 | 0.3 |
| 0 | 913 | 22.9 | 0.2 | 0.1 | 0.6 | 0.1 | 0.1 | 0.1 | 0.6 | 0.1 | 0.2 | 0.1 | 0.1 | 0.1 | 0.1 | 0.5 | 0.1 | 0.1 | 0.1 | 0.1 | 0.6 | 0.1 | 0.2 | 0.1 | 0.1 | 0.1 | 0.2 | 0.1 | 0.6 |
| 0 | 980 | 23.0 | 0.2 | 0.2 | 0.2 | 0.1 | 0.6 | 0.1 | 0.1 | 0.6 | 0.1 | 0.2 | 0.2 | 0.2 | 0.1 | 0.2 | 0.1 | 0.1 | 0.2 | 0.1 | 0.1 | 0.6 | 0.1 | 0.2 | 0.6 | 0.2 | 0.1 | 0.2 | 0.1 |
| 0 | 2211 | 59.9 | 0.2 | 0.2 | 0.5 | 0.2 | 0.4 | 0.2 | 0.5 | 0.2 | 0.2 | 0.2 | 0.4 | 0.2 | 0.4 | 0.4 | 0.4 | 0.2 | 0.4 | 0.2 | 0.5 | 0.2 | 0.2 | 0.2 | 0.4 | 0.2 | 0.2 | 0.2 | 0.5 |
| 0 | 2328 | 54.8 | 0.2 | 0.3 | 0.1 | 0.3 | 0.4 | 0.3 | 0.1 | 0.3 | 0.2 | 0.3 | 0.4 | 0.3 | 0.4 | 0.3 | 0.4 | 0.3 | 0.4 | 0.3 | 0.1 | 0.3 | 0.2 | 0.3 | 0.4 | 0.3 | 0.2 | 0.3 | 0.1 |
| 0 | 2431 | 67.8 | 0.2 | 0.3 | 0.1 | 0.3 | 0.5 | 0.3 | 0.1 | 0.3 | 0.2 | 0.3 | 0.5 | 0.3 | 0.5 | 0.5 | 0.5 | 0.3 | 0.5 | 0.3 | 0.1 | 0.3 | 0.2 | 0.3 | 0.5 | 0.3 | 0.2 | 0.3 | 0.1 |
| 0 | 1354 | 36.9 | 0.2 | 0.3 | 0.2 | 0.1 | 0.2 | 0.1 | 0.3 | 0.2 | 0.3 | 0.2 | 0.3 | 0.2 | 0.3 | 0.3 | 0.3 | 0.1 | 0.3 | 0.1 | 0.1 | 0.2 | 0.1 | 0.2 | 0.2 | 0.2 | 0.3 | 0.3 | 0.3 |
| 0 | 1289 | 35.7 | 0.2 | 0.3 | 0.2 | 0.3 | 0.2 | 0.3 | 0.1 | 0.2 | 0.1 | 0.2 | 0.3 | 0.2 | 0.1 | 0.3 | 0.1 | 0.3 | 0.3 | 0.3 | 0.3 | 0.2 | 0.3 | 0.2 | 0.2 | 0.2 | 0.1 | 0.3 | 0.1 |
| 0 | 2898 | 73.1 | 0.2 | 0.4 | 0.2 | 0.2 | 0.4 | 0.2 | 0.2 | 0.4 | 0.2 | 0.4 | 0.4 | 0.4 | 0.5 | 0.5 | 0.5 | 0.4 | 0.4 | 0.4 | 0.2 | 0.4 | 0.2 | 0.2 | 0.4 | 0.2 | 0.2 | 0.4 | 0.2 |
| 0 | 2543 | 61.4 | 0.2 | 0.4 | 0.2 | 0.3 | 0.5 | 0.3 | 0.3 | 0.5 | 0.3 | 0.2 | 0.4 | 0.2 | 0.3 | 0.4 | 0.3 | 0.3 | 0.4 | 0.3 | 0.3 | 0.5 | 0.3 | 0.2 | 0.5 | 0.2 | 0.3 | 0.4 | 0.3 |
| 0 | 1522 | 34.6 | 0.2 | 0.4 | 0.4 | 0.2 | 0.4 | 0.4 | 0.2 | 0.4 | 0.4 | 0.1 | 0.1 | 0.1 | 0.1 | 0.1 | 0.1 | 0.1 | 0.1 | 0.1 | 0.2 | 0.4 | 0.4 | 0.2 | 0.4 | 0.4 | 0.2 | 0.4 | 0.4 |
| 0 | 3455 | 76.0 | 0.2 | 0.4 | 0.5 | 0.2 | 0.4 | 0.5 | 0.2 | 0.4 | 0.5 | 0.4 | 0.4 | 0.4 | 0.4 | 0.4 | 0.4 | 0.4 | 0.4 | 0.4 | 0.2 | 0.4 | 0.5 | 0.2 | 0.4 | 0.5 | 0.2 | 0.4 | 0.5 |
| 0 | 975 | 21.1 | 0.2 | 0.5 | 0.2 | 0.1 | 0.1 | 0.1 | 0.1 | 0.1 | 0.1 | 0.2 | 0.5 | 0.2 | 0.1 | 0.5 | 0.1 | 0.1 | 0.5 | 0.1 | 0.1 | 0.1 | 0.1 | 0.2 | 0.1 | 0.2 | 0.1 | 0.5 | 0.1 |
| 0 | 2382 | 54.0 | 0.2 | 0.5 | 0.2 | 0.3 | 0.6 | 0.3 | 0.2 | 0.6 | 0.2 | 0.2 | 0.5 | 0.2 | 0.2 | 0.5 | 0.2 | 0.3 | 0.5 | 0.3 | 0.3 | 0.6 | 0.3 | 0.2 | 0.6 | 0.2 | 0.2 | 0.5 | 0.2 |
| 0 | 3685 | 84.0 | 0.2 | 0.5 | 0.2 | 0.6 | 0.6 | 0.6 | 0.1 | 0.6 | 0.1 | 0.2 | 0.5 | 0.2 | 0.1 | 0.5 | 0.1 | 0.6 | 0.5 | 0.6 | 0.6 | 0.6 | 0.6 | 0.2 | 0.6 | 0.2 | 0.1 | 0.5 | 0.1 |
| 0 | 2131 | 60.7 | 0.2 | 0.5 | 0.3 | 0.2 | 0.5 | 0.3 | 0.2 | 0.5 | 0.3 | 0.1 | 0.1 | 0.1 | 0.5 | 0.5 | 0.5 | 0.1 | 0.1 | 0.1 | 0.2 | 0.5 | 0.3 | 0.2 | 0.5 | 0.3 | 0.2 | 0.5 | 0.3 |

| | | | | | | | | | | | | | | | | | | | | | | | | | | | | | |
|---|---|---|---|---|---|---|---|---|---|---|---|---|---|---|---|---|---|---|---|---|---|---|---|---|---|---|---|---|---|
| 0 | 4272 | 95.4 | 0.2 | 0.6 | 0.1 | 0.2 | 0.6 | 0.1 | 0.2 | 0.6 | 0.1 | 0.6 | 0.6 | 0.6 | 0.6 | 0.6 | 0.6 | 0.6 | 0.6 | 0.6 | 0.2 | 0.6 | 0.1 | 0.2 | 0.6 | 0.1 | 0.2 | 0.6 | 0.1 |
| 0 | 3023 | 68.4 | 0.2 | 0.6 | 0.4 | 0.2 | 0.6 | 0.4 | 0.2 | 0.6 | 0.4 | 0.4 | 0.4 | 0.4 | 0.2 | 0.2 | 0.2 | 0.4 | 0.4 | 0.4 | 0.2 | 0.6 | 0.4 | 0.2 | 0.6 | 0.4 | 0.2 | 0.6 | 0.4 |
| 0 | 868 | 23.4 | 0.3 | 0.1 | 0.3 | 0.3 | 0.1 | 0.3 | 0.1 | 0.1 | 0.1 | 0.3 | 0.1 | 0.3 | 0.1 | 0.1 | 0.1 | 0.3 | 0.1 | 0.3 | 0.3 | 0.1 | 0.3 | 0.3 | 0.1 | 0.3 | 0.1 | 0.1 | 0.1 |
| 0 | 1630 | 38.9 | 0.3 | 0.1 | 0.5 | 0.1 | 0.3 | 0.1 | 0.5 | 0.1 | 0.3 | 0.1 | 0.3 | 0.1 | 0.3 | 0.5 | 0.3 | 0.1 | 0.3 | 0.1 | 0.5 | 0.1 | 0.3 | 0.1 | 0.3 | 0.1 | 0.3 | 0.1 | 0.5 |
| 0 | 1276 | 34.8 | 0.3 | 0.2 | 0.3 | 0.3 | 0.2 | 0.3 | 0.3 | 0.2 | 0.3 | 0.1 | 0.1 | 0.1 | 0.2 | 0.2 | 0.2 | 0.1 | 0.1 | 0.1 | 0.3 | 0.2 | 0.3 | 0.3 | 0.2 | 0.3 | 0.3 | 0.2 | 0.3 |
| 0 | 1697 | 41.8 | 0.3 | 0.2 | 0.4 | 0.2 | 0.2 | 0.2 | 0.4 | 0.2 | 0.3 | 0.2 | 0.2 | 0.2 | 0.2 | 0.3 | 0.2 | 0.2 | 0.2 | 0.2 | 0.4 | 0.2 | 0.3 | 0.2 | 0.2 | 0.2 | 0.3 | 0.2 | 0.4 |
| 0 | 2430 | 64.4 | 0.3 | 0.2 | 0.5 | 0.3 | 0.2 | 0.5 | 0.3 | 0.2 | 0.5 | 0.3 | 0.3 | 0.3 | 0.4 | 0.4 | 0.4 | 0.3 | 0.3 | 0.3 | 0.3 | 0.2 | 0.5 | 0.3 | 0.2 | 0.5 | 0.3 | 0.2 | 0.5 |
| 0 | 2362 | 56.0 | 0.4 | 0.4 | 0.3 | 0.4 | 0.1 | 0.4 | 0.3 | 0.4 | 0.4 | 0.4 | 0.1 | 0.4 | 0.1 | 0.1 | 0.1 | 0.4 | 0.1 | 0.4 | 0.3 | 0.4 | 0.4 | 0.4 | 0.1 | 0.4 | 0.4 | 0.4 | 0.3 |
| 0 | 2612 | 61.1 | 0.4 | 0.4 | 0.3 | 0.4 | 0.1 | 0.4 | 0.3 | 0.4 | 0.4 | 0.4 | 0.1 | 0.4 | 0.1 | 0.3 | 0.1 | 0.4 | 0.1 | 0.4 | 0.3 | 0.4 | 0.4 | 0.4 | 0.1 | 0.4 | 0.4 | 0.4 | 0.3 |
| 0 | 3159 | 79.8 | 0.4 | 0.4 | 0.4 | 0.2 | 0.2 | 0.2 | 0.5 | 0.2 | 0.5 | 0.4 | 0.4 | 0.4 | 0.5 | 0.4 | 0.5 | 0.2 | 0.4 | 0.2 | 0.2 | 0.2 | 0.2 | 0.4 | 0.2 | 0.4 | 0.5 | 0.4 | 0.5 |
| 0 | 2299 | 52.5 | 0.4 | 0.4 | 0.4 | 0.3 | 0.2 | 0.3 | 0.1 | 0.2 | 0.1 | 0.4 | 0.4 | 0.4 | 0.1 | 0.4 | 0.1 | 0.3 | 0.4 | 0.3 | 0.3 | 0.2 | 0.3 | 0.4 | 0.2 | 0.4 | 0.1 | 0.4 | 0.1 |
| 0 | 3080 | 74.0 | 0.4 | 0.5 | 0.1 | 0.4 | 0.5 | 0.1 | 0.4 | 0.5 | 0.1 | 0.5 | 0.5 | 0.5 | 0.4 | 0.4 | 0.4 | 0.5 | 0.5 | 0.5 | 0.4 | 0.5 | 0.1 | 0.4 | 0.5 | 0.1 | 0.4 | 0.5 | 0.1 |
| 0 | 3197 | 68.4 | 0.5 | 0.1 | 0.5 | 0.5 | 0.1 | 0.5 | 0.5 | 0.1 | 0.5 | 0.3 | 0.3 | 0.3 | 0.6 | 0.6 | 0.6 | 0.3 | 0.3 | 0.3 | 0.5 | 0.1 | 0.5 | 0.5 | 0.1 | 0.5 | 0.5 | 0.1 | 0.5 |
| 0 | 1394 | 40.1 | 0.5 | 0.2 | 0.2 | 0.2 | 0.2 | 0.2 | 0.2 | 0.2 | 0.5 | 0.2 | 0.2 | 0.2 | 0.2 | 0.1 | 0.2 | 0.2 | 0.2 | 0.2 | 0.2 | 0.2 | 0.5 | 0.2 | 0.2 | 0.2 | 0.5 | 0.2 | 0.2 |
| 0 | 1464 | 40.1 | 0.5 | 0.2 | 0.3 | 0.2 | 0.1 | 0.2 | 0.3 | 0.2 | 0.5 | 0.2 | 0.1 | 0.2 | 0.1 | 0.6 | 0.1 | 0.2 | 0.1 | 0.2 | 0.3 | 0.2 | 0.5 | 0.2 | 0.1 | 0.2 | 0.5 | 0.2 | 0.3 |
| 0 | 3723 | 82.1 | 0.5 | 0.2 | 0.3 | 0.5 | 0.2 | 0.3 | 0.5 | 0.2 | 0.3 | 0.5 | 0.5 | 0.5 | 0.4 | 0.4 | 0.4 | 0.5 | 0.5 | 0.5 | 0.5 | 0.2 | 0.3 | 0.5 | 0.2 | 0.3 | 0.5 | 0.2 | 0.3 |
| 0 | 3877 | 78.8 | 0.5 | 0.2 | 0.4 | 0.5 | 0.2 | 0.4 | 0.5 | 0.2 | 0.4 | 0.5 | 0.5 | 0.5 | 0.3 | 0.3 | 0.3 | 0.5 | 0.5 | 0.5 | 0.5 | 0.2 | 0.4 | 0.5 | 0.2 | 0.4 | 0.5 | 0.2 | 0.4 |
| 0 | 1852 | 43.1 | 0.5 | 0.2 | 0.5 | 0.2 | 0.2 | 0.2 | 0.5 | 0.2 | 0.5 | 0.2 | 0.2 | 0.2 | 0.2 | 0.1 | 0.2 | 0.2 | 0.2 | 0.2 | 0.5 | 0.2 | 0.5 | 0.2 | 0.2 | 0.2 | 0.5 | 0.2 | 0.5 |
| 0 | 3540 | 79.5 | 0.5 | 0.2 | 0.6 | 0.2 | 0.5 | 0.2 | 0.6 | 0.2 | 0.5 | 0.2 | 0.5 | 0.2 | 0.5 | 0.5 | 0.5 | 0.2 | 0.5 | 0.2 | 0.6 | 0.2 | 0.5 | 0.2 | 0.5 | 0.2 | 0.5 | 0.2 | 0.6 |
| 0 | 2550 | 65.5 | 0.5 | 0.3 | 0.1 | 0.3 | 0.3 | 0.3 | 0.1 | 0.3 | 0.5 | 0.3 | 0.3 | 0.3 | 0.3 | 0.5 | 0.3 | 0.3 | 0.3 | 0.3 | 0.1 | 0.3 | 0.5 | 0.3 | 0.3 | 0.3 | 0.5 | 0.3 | 0.1 |
| 0 | 3210 | 64.2 | 0.5 | 0.3 | 0.6 | 0.3 | 0.2 | 0.3 | 0.6 | 0.3 | 0.5 | 0.3 | 0.2 | 0.3 | 0.2 | 0.5 | 0.2 | 0.3 | 0.2 | 0.3 | 0.6 | 0.3 | 0.5 | 0.3 | 0.2 | 0.3 | 0.5 | 0.3 | 0.6 |
| 0 | 3349 | 67.9 | 0.5 | 0.4 | 0.5 | 0.1 | 0.1 | 0.1 | 0.5 | 0.1 | 0.5 | 0.5 | 0.4 | 0.5 | 0.5 | 0.4 | 0.5 | 0.1 | 0.4 | 0.1 | 0.1 | 0.1 | 0.1 | 0.5 | 0.1 | 0.5 | 0.5 | 0.4 | 0.5 |
| 0 | 4934 | 99.8 | 0.5 | 0.4 | 0.5 | 0.1 | 0.4 | 0.1 | 0.6 | 0.4 | 0.6 | 0.5 | 0.4 | 0.5 | 0.6 | 0.4 | 0.6 | 0.1 | 0.4 | 0.1 | 0.1 | 0.4 | 0.1 | 0.5 | 0.4 | 0.5 | 0.6 | 0.4 | 0.6 |
| 0 | 3982 | 76.0 | 0.5 | 0.4 | 0.5 | 0.3 | 0.1 | 0.3 | 0.5 | 0.1 | 0.5 | 0.5 | 0.4 | 0.5 | 0.5 | 0.4 | 0.5 | 0.3 | 0.4 | 0.3 | 0.3 | 0.1 | 0.3 | 0.5 | 0.1 | 0.5 | 0.5 | 0.4 | 0.5 |
| 0 | 4010 | 92.0 | 0.5 | 0.4 | 0.5 | 0.4 | 0.3 | 0.4 | 0.5 | 0.4 | 0.5 | 0.4 | 0.3 | 0.4 | 0.3 | 0.1 | 0.3 | 0.4 | 0.3 | 0.4 | 0.5 | 0.4 | 0.5 | 0.4 | 0.3 | 0.4 | 0.5 | 0.4 | 0.5 |
| 0 | 1927 | 38.7 | 0.5 | 0.5 | 0.1 | 0.5 | 0.5 | 0.1 | 0.5 | 0.5 | 0.1 | 0.3 | 0.3 | 0.3 | 0.2 | 0.2 | 0.2 | 0.3 | 0.3 | 0.3 | 0.5 | 0.5 | 0.1 | 0.5 | 0.5 | 0.1 | 0.5 | 0.5 | 0.1 |
| 0 | 2191 | 44.9 | 0.5 | 0.5 | 0.1 | 0.5 | 0.5 | 0.1 | 0.5 | 0.5 | 0.1 | 0.4 | 0.4 | 0.4 | 0.1 | 0.1 | 0.1 | 0.4 | 0.4 | 0.4 | 0.5 | 0.5 | 0.1 | 0.5 | 0.5 | 0.1 | 0.5 | 0.5 | 0.1 |
| 0 | 3474 | 72.6 | 0.5 | 0.5 | 0.1 | 0.5 | 0.5 | 0.1 | 0.5 | 0.5 | 0.1 | 0.5 | 0.5 | 0.5 | 0.4 | 0.4 | 0.4 | 0.5 | 0.5 | 0.5 | 0.5 | 0.5 | 0.1 | 0.5 | 0.5 | 0.1 | 0.5 | 0.5 | 0.1 |
| 0 | 2870 | 71.4 | 0.5 | 0.5 | 0.5 | 0.1 | 0.5 | 0.1 | 0.2 | 0.5 | 0.2 | 0.5 | 0.5 | 0.5 | 0.2 | 0.5 | 0.2 | 0.1 | 0.5 | 0.1 | 0.1 | 0.5 | 0.1 | 0.5 | 0.5 | 0.5 | 0.2 | 0.5 | 0.2 |
| 0 | 4653 | 80.4 | 0.5 | 0.5 | 0.5 | 0.5 | 0.1 | 0.5 | 0.5 | 0.5 | 0.5 | 0.5 | 0.1 | 0.5 | 0.1 | 0.1 | 0.1 | 0.5 | 0.1 | 0.5 | 0.5 | 0.5 | 0.5 | 0.5 | 0.1 | 0.5 | 0.5 | 0.5 | 0.5 |
| 0 | 4845 | 89.9 | 0.5 | 0.5 | 0.5 | 0.5 | 0.5 | 0.5 | 0.5 | 0.5 | 0.5 | 0.1 | 0.1 | 0.1 | 0.2 | 0.2 | 0.2 | 0.1 | 0.1 | 0.1 | 0.5 | 0.5 | 0.5 | 0.5 | 0.5 | 0.5 | 0.5 | 0.5 | 0.5 |
| 0 | 2815 | 58.6 | 0.6 | 0.1 | 0.4 | 0.6 | 0.1 | 0.4 | 0.6 | 0.1 | 0.4 | 0.4 | 0.4 | 0.4 | 0.3 | 0.3 | 0.3 | 0.4 | 0.4 | 0.4 | 0.6 | 0.1 | 0.4 | 0.6 | 0.1 | 0.4 | 0.6 | 0.1 | 0.4 |
| 0 | 1781 | 44.3 | 0.6 | 0.2 | 0.2 | 0.2 | 0.2 | 0.2 | 0.2 | 0.2 | 0.6 | 0.2 | 0.2 | 0.2 | 0.2 | 0.6 | 0.2 | 0.2 | 0.2 | 0.2 | 0.2 | 0.2 | 0.6 | 0.2 | 0.2 | 0.2 | 0.6 | 0.2 | 0.2 |
| 0 | 3820 | 80.6 | 0.6 | 0.2 | 0.3 | 0.6 | 0.2 | 0.3 | 0.6 | 0.2 | 0.3 | 0.5 | 0.5 | 0.5 | 0.4 | 0.4 | 0.4 | 0.5 | 0.5 | 0.5 | 0.6 | 0.2 | 0.3 | 0.6 | 0.2 | 0.3 | 0.6 | 0.2 | 0.3 |
| 0 | 3068 | 63.6 | 0.6 | 0.2 | 0.6 | 0.2 | 0.3 | 0.2 | 0.5 | 0.3 | 0.5 | 0.6 | 0.2 | 0.6 | 0.5 | 0.2 | 0.5 | 0.2 | 0.2 | 0.2 | 0.2 | 0.3 | 0.2 | 0.6 | 0.3 | 0.6 | 0.5 | 0.2 | 0.5 |
| 0 | 3054 | 53.3 | 0.6 | 0.2 | 0.6 | 0.6 | 0.2 | 0.6 | 0.6 | 0.2 | 0.6 | 0.1 | 0.1 | 0.1 | 0.4 | 0.4 | 0.4 | 0.1 | 0.1 | 0.1 | 0.6 | 0.2 | 0.6 | 0.6 | 0.2 | 0.6 | 0.6 | 0.2 | 0.6 |
| 0 | 3202 | 57.7 | 0.6 | 0.2 | 0.6 | 0.6 | 0.2 | 0.6 | 0.6 | 0.2 | 0.6 | 0.3 | 0.3 | 0.3 | 0.2 | 0.2 | 0.2 | 0.3 | 0.3 | 0.3 | 0.6 | 0.2 | 0.6 | 0.6 | 0.2 | 0.6 | 0.6 | 0.2 | 0.6 |
| 0 | 2417 | 67.3 | 0.6 | 0.3 | 0.1 | 0.3 | 0.3 | 0.3 | 0.1 | 0.3 | 0.6 | 0.3 | 0.3 | 0.3 | 0.3 | 0.4 | 0.3 | 0.3 | 0.3 | 0.3 | 0.1 | 0.3 | 0.6 | 0.3 | 0.3 | 0.3 | 0.6 | 0.3 | 0.1 |
| 0 | 3661 | 90.9 | 0.4 | 0.5 | 0.2 | 0.4 | 0.5 | 0.2 | 0.4 | 0.5 | 0.2 | 0.4 | 0.4 | 0.4 | 0.6 | 0.6 | 0.6 | 0.4 | 0.4 | 0.4 | 0.4 | 0.5 | 0.2 | 0.4 | 0.5 | 0.2 | 0.4 | 0.5 | 0.2 |
| 0 | 1578 | 45.3 | 0.1 | 0.1 | 0.1 | 0.1 | 0.1 | 0.1 | 0.1 | 0.1 | 0.1 | 0.3 | 0.3 | 0.3 | 0.6 | 0.6 | 0.6 | 0.3 | 0.3 | 0.3 | 0.1 | 0.1 | 0.1 | 0.1 | 0.1 | 0.1 | 0.1 | 0.1 | 0.1 |
| 0 | 2821 | 60.8 | 0.2 | 0.3 | 0.2 | 0.1 | 0.4 | 0.1 | 0.6 | 0.4 | 0.6 | 0.2 | 0.3 | 0.2 | 0.6 | 0.3 | 0.6 | 0.1 | 0.3 | 0.1 | 0.1 | 0.4 | 0.1 | 0.2 | 0.4 | 0.2 | 0.6 | 0.3 | 0.6 |

| | | | | | | | | | | | | | | | | | | | | | | | | | | | | | |
|---|---|---|---|---|---|---|---|---|---|---|---|---|---|---|---|---|---|---|---|---|---|---|---|---|---|---|---|---|---|
| 0 | 1552 | 37.4 | 0.1 | 0.2 | 0.1 | 0.4 | 0.1 | 0.4 | 0.5 | 0.1 | 0.5 | 0.1 | 0.2 | 0.1 | 0.5 | 0.2 | 0.5 | 0.4 | 0.2 | 0.4 | 0.4 | 0.1 | 0.4 | 0.1 | 0.1 | 0.1 | 0.5 | 0.2 | 0.5 |
| 0 | 2235 | 59.4 | 0.6 | 0.3 | 0.2 | 0.3 | 0.2 | 0.3 | 0.2 | 0.3 | 0.6 | 0.3 | 0.2 | 0.3 | 0.2 | 0.3 | 0.2 | 0.3 | 0.2 | 0.3 | 0.2 | 0.3 | 0.6 | 0.3 | 0.2 | 0.3 | 0.6 | 0.3 | 0.2 |
| 0 | 2314 | 57.0 | 0.6 | 0.4 | 0.2 | 0.4 | 0.1 | 0.4 | 0.2 | 0.4 | 0.6 | 0.4 | 0.1 | 0.4 | 0.1 | 0.1 | 0.1 | 0.4 | 0.1 | 0.4 | 0.2 | 0.4 | 0.6 | 0.4 | 0.1 | 0.4 | 0.6 | 0.4 | 0.2 |
| 0 | 3062 | 60.6 | 0.6 | 0.4 | 0.1 | 0.6 | 0.4 | 0.1 | 0.6 | 0.4 | 0.1 | 0.5 | 0.5 | 0.5 | 0.2 | 0.2 | 0.2 | 0.5 | 0.5 | 0.5 | 0.6 | 0.4 | 0.1 | 0.6 | 0.4 | 0.1 | 0.6 | 0.4 | 0.1 |
| 0 | 4087 | 80.7 | 0.5 | 0.6 | 0.5 | 0.5 | 0.1 | 0.5 | 0.1 | 0.1 | 0.1 | 0.5 | 0.6 | 0.5 | 0.1 | 0.6 | 0.1 | 0.5 | 0.6 | 0.5 | 0.5 | 0.1 | 0.5 | 0.5 | 0.1 | 0.5 | 0.1 | 0.6 | 0.1 |
| 0 | 2266 | 45.5 | 0.6 | 0.2 | 0.6 | 0.2 | 0.2 | 0.2 | 0.6 | 0.2 | 0.6 | 0.2 | 0.2 | 0.2 | 0.2 | 0.4 | 0.2 | 0.2 | 0.2 | 0.2 | 0.6 | 0.2 | 0.6 | 0.2 | 0.2 | 0.2 | 0.6 | 0.2 | 0.6 |
| 0 | 2294 | 49.1 | 0.3 | 0.1 | 0.3 | 0.5 | 0.3 | 0.5 | 0.4 | 0.3 | 0.4 | 0.3 | 0.1 | 0.3 | 0.4 | 0.1 | 0.4 | 0.5 | 0.1 | 0.5 | 0.5 | 0.3 | 0.5 | 0.3 | 0.3 | 0.3 | 0.4 | 0.1 | 0.4 |
| 0 | 1243 | 33.6 | 0.1 | 0.2 | 0.1 | 0.3 | 0.5 | 0.3 | 0.1 | 0.5 | 0.1 | 0.1 | 0.2 | 0.1 | 0.1 | 0.2 | 0.1 | 0.3 | 0.2 | 0.3 | 0.3 | 0.5 | 0.3 | 0.1 | 0.5 | 0.1 | 0.1 | 0.2 | 0.1 |
| 0 | 2078 | 50.9 | 0.2 | 0.1 | 0.3 | 0.2 | 0.1 | 0.3 | 0.2 | 0.1 | 0.3 | 0.4 | 0.4 | 0.4 | 0.4 | 0.4 | 0.4 | 0.4 | 0.4 | 0.4 | 0.2 | 0.1 | 0.3 | 0.2 | 0.1 | 0.3 | 0.2 | 0.1 | 0.3 |
| 0 | 2416 | 62.2 | 0.3 | 0.4 | 0.1 | 0.3 | 0.4 | 0.1 | 0.3 | 0.4 | 0.1 | 0.3 | 0.3 | 0.3 | 0.6 | 0.6 | 0.6 | 0.3 | 0.3 | 0.3 | 0.3 | 0.4 | 0.1 | 0.3 | 0.4 | 0.1 | 0.3 | 0.4 | 0.1 |
| 0 | 2648 | 61.6 | 0.3 | 0.6 | 0.2 | 0.3 | 0.6 | 0.2 | 0.3 | 0.6 | 0.2 | 0.3 | 0.3 | 0.3 | 0.3 | 0.3 | 0.3 | 0.3 | 0.3 | 0.3 | 0.3 | 0.6 | 0.2 | 0.3 | 0.6 | 0.2 | 0.3 | 0.6 | 0.2 |
| 0 | 3547 | 85.6 | 0.3 | 0.4 | 0.3 | 0.4 | 0.2 | 0.4 | 0.6 | 0.2 | 0.6 | 0.3 | 0.4 | 0.3 | 0.6 | 0.4 | 0.6 | 0.4 | 0.4 | 0.4 | 0.4 | 0.2 | 0.4 | 0.3 | 0.2 | 0.3 | 0.6 | 0.4 | 0.6 |
| 0 | 1536 | 35.8 | 0.1 | 0.5 | 0.1 | 0.1 | 0.5 | 0.1 | 0.3 | 0.5 | 0.3 | 0.1 | 0.5 | 0.1 | 0.3 | 0.5 | 0.3 | 0.1 | 0.5 | 0.1 | 0.1 | 0.5 | 0.1 | 0.1 | 0.5 | 0.1 | 0.3 | 0.5 | 0.3 |
| 0 | 3051 | 70.7 | 0.3 | 0.4 | 0.5 | 0.4 | 0.2 | 0.4 | 0.5 | 0.4 | 0.3 | 0.4 | 0.2 | 0.4 | 0.2 | 0.2 | 0.2 | 0.4 | 0.2 | 0.4 | 0.5 | 0.4 | 0.3 | 0.4 | 0.2 | 0.4 | 0.3 | 0.4 | 0.5 |
| 0 | 3095 | 71.3 | 0.6 | 0.4 | 0.4 | 0.4 | 0.1 | 0.4 | 0.4 | 0.4 | 0.6 | 0.4 | 0.1 | 0.4 | 0.1 | 0.2 | 0.1 | 0.4 | 0.1 | 0.4 | 0.4 | 0.4 | 0.6 | 0.4 | 0.1 | 0.4 | 0.6 | 0.4 | 0.4 |
| 0 | 3236 | 76.4 | 0.5 | 0.6 | 0.5 | 0.1 | 0.2 | 0.1 | 0.3 | 0.2 | 0.3 | 0.5 | 0.6 | 0.5 | 0.3 | 0.6 | 0.3 | 0.1 | 0.6 | 0.1 | 0.1 | 0.2 | 0.1 | 0.5 | 0.2 | 0.5 | 0.3 | 0.6 | 0.3 |
| 0 | 1818 | 36.0 | 0.6 | 0.1 | 0.5 | 0.6 | 0.1 | 0.5 | 0.6 | 0.1 | 0.5 | 0.1 | 0.1 | 0.1 | 0.3 | 0.3 | 0.3 | 0.1 | 0.1 | 0.1 | 0.6 | 0.1 | 0.5 | 0.6 | 0.1 | 0.5 | 0.6 | 0.1 | 0.5 |
| 0 | 2107 | 52.0 | 0.1 | 0.8 | 0.1 | 0.6 | 0.2 | 0.8 | 0.1 | 0.5 | 0.2 | 0.4 | 0.2 | 0.2 | 0.2 | 0.2 | 0.6 | 0.2 | 0.2 | 0.2 | 0.4 | 0.1 | 0.5 | 0.1 | 0.5 | 0.2 | 0.3 | 0.8 | 0.2 |
| 0 | 1932 | 47.7 | 0.1 | 0.2 | 0.7 | 0.4 | 0.3 | 0.4 | 0.3 | 0.7 | 0.1 | 0.3 | 0.4 | 0.2 | 0.3 | 0.2 | 0.2 | 0.6 | 0.3 | 0.2 | 0.3 | 0.1 | 0.3 | 0.4 | 0.1 | 0.5 | 0.7 | 0.1 | 0.3 |
| 0 | 2349 | 52.5 | 0.1 | 0.6 | 0.2 | 0.2 | 0.2 | 0.8 | 0.7 | 0.2 | 0.1 | 0.7 | 0.3 | 0.2 | 0.1 | 0.3 | 0.6 | 0.1 | 0.3 | 0.6 | 0.2 | 0.1 | 0.4 | 0.4 | 0.3 | 0.3 | 0.3 | 0.2 | 0.1 |
| 0 | 1518 | 36.2 | 0.6 | 0.4 | 0.1 | 0.3 | 0.2 | 0.1 | 0.6 | 0.1 | 0.1 | 0.1 | 0.1 | 0.2 | 0.7 | 0.4 | 0.2 | 0.5 | 0.6 | 0.3 | 0.2 | 0.2 | 0.3 | 0.2 | 0.1 | 0.1 | 0.2 | 0.3 | 0.1 |
| 1 | 2722 | 70.3 | 0.4 | 0.3 | 0.2 | 0.4 | 0.1 | 0.3 | 0.1 | 0.4 | 0.5 | 0.3 | 0.3 | 0.3 | 0.5 | 0.5 | 0.4 | 0.4 | 0.3 | 0.2 | 0.2 | 0.3 | 0.8 | 0.4 | 0.3 | 0.3 | 0.6 | 0.3 | 0.1 |
| 1 | 2704 | 69.0 | 0.7 | 0.3 | 0.2 | 0.3 | 0.1 | 0.3 | 0.1 | 0.3 | 0.4 | 0.3 | 0.3 | 0.3 | 0.6 | 0.5 | 0.4 | 0.4 | 0.3 | 0.2 | 0.1 | 0.3 | 0.8 | 0.4 | 0.3 | 0.3 | 0.7 | 0.4 | 0.1 |
| 1 | 2738 | 64.9 | 0.2 | 0.8 | 0.1 | 0.3 | 0.1 | 0.3 | 0.1 | 0.7 | 0.2 | 0.1 | 0.4 | 0.3 | 0.5 | 0.7 | 0.5 | 0.1 | 0.4 | 0.3 | 0.2 | 0.3 | 0.6 | 0.4 | 0.7 | 0.4 | 0.1 | 0.8 | 0.2 |
| 1 | 2441 | 65.3 | 0.2 | 0.3 | 0.1 | 0.4 | 0.2 | 0.5 | 0.1 | 0.3 | 0.8 | 0.4 | 0.3 | 0.3 | 0.3 | 0.7 | 0.4 | 0.1 | 0.3 | 0.3 | 0.4 | 0.3 | 0.8 | 0.3 | 0.1 | 0.4 | 0.7 | 0.3 | 0.1 |
| 1 | 2750 | 61.8 | 0.3 | 0.2 | 0.5 | 0.3 | 0.2 | 0.3 | 0.3 | 0.3 | 0.5 | 0.3 | 0.3 | 0.3 | 0.4 | 0.4 | 0.6 | 0.3 | 0.3 | 0.3 | 0.3 | 0.2 | 0.8 | 0.3 | 0.2 | 0.5 | 0.3 | 0.2 | 0.5 |
| 1 | 2735 | 67.7 | 0.3 | 0.7 | 0.3 | 0.1 | 0.1 | 0.2 | 0.1 | 0.5 | 0.1 | 0.3 | 0.3 | 0.2 | 0.6 | 0.8 | 0.5 | 0.1 | 0.3 | 0.3 | 0.4 | 0.4 | 0.1 | 0.4 | 0.3 | 0.4 | 0.4 | 0.8 | 0.1 |
| 1 | 2625 | 66.6 | 0.6 | 0.3 | 0.1 | 0.3 | 0.3 | 0.3 | 0.1 | 0.3 | 0.7 | 0.4 | 0.3 | 0.3 | 0.6 | 0.4 | 0.3 | 0.3 | 0.3 | 0.3 | 0.1 | 0.3 | 0.8 | 0.3 | 0.3 | 0.3 | 0.8 | 0.3 | 0.1 |
| 1 | 2763 | 73.6 | 0.7 | 0.2 | 0.3 | 0.4 | 0.3 | 0.2 | 0.1 | 0.3 | 0.5 | 0.3 | 0.3 | 0.3 | 0.7 | 0.6 | 0.4 | 0.3 | 0.3 | 0.3 | 0.4 | 0.2 | 0.8 | 0.3 | 0.2 | 0.3 | 0.8 | 0.2 | 0.1 |
| 1 | 2733 | 66.2 | 0.3 | 0.1 | 0.4 | 0.4 | 0.1 | 0.4 | 0.2 | 0.3 | 0.1 | 0.3 | 0.3 | 0.3 | 0.4 | 0.4 | 0.6 | 0.3 | 0.7 | 0.3 | 0.3 | 0.2 | 0.8 | 0.4 | 0.3 | 0.5 | 0.7 | 0.2 | 0.2 |
| 1 | 2731 | 66.3 | 0.2 | 0.2 | 0.1 | 0.3 | 0.2 | 0.3 | 0.2 | 0.3 | 0.2 | 0.2 | 0.5 | 0.3 | 0.5 | 0.7 | 0.5 | 0.4 | 0.6 | 0.3 | 0.1 | 0.3 | 0.2 | 0.3 | 0.8 | 0.3 | 0.4 | 0.3 | 0.1 |
| 1 | 2777 | 68.4 | 0.3 | 0.4 | 0.1 | 0.1 | 0.2 | 0.1 | 0.2 | 0.8 | 0.3 | 0.2 | 0.3 | 0.3 | 0.4 | 0.7 | 0.5 | 0.3 | 0.4 | 0.3 | 0.3 | 0.4 | 0.6 | 0.3 | 0.4 | 0.3 | 0.2 | 0.8 | 0.2 |
| 1 | 3030 | 59.0 | 0.3 | 0.8 | 0.1 | 0.1 | 0.2 | 0.2 | 0.1 | 0.7 | 0.3 | 0.3 | 0.3 | 0.3 | 0.4 | 0.8 | 0.5 | 0.2 | 0.4 | 0.3 | 0.3 | 0.4 | 0.7 | 0.2 | 0.7 | 0.3 | 0.2 | 0.8 | 0.2 |
| 1 | 2701 | 70.3 | 0.5 | 0.2 | 0.1 | 0.3 | 0.3 | 0.3 | 0.1 | 0.3 | 0.6 | 0.3 | 0.3 | 0.3 | 0.3 | 0.5 | 0.4 | 0.2 | 0.3 | 0.3 | 0.4 | 0.3 | 0.6 | 0.3 | 0.3 | 0.4 | 0.6 | 0.3 | 0.1 |
| 1 | 2877 | 59.6 | 0.3 | 0.8 | 0.1 | 0.1 | 0.2 | 0.2 | 0.1 | 0.7 | 0.3 | 0.3 | 0.3 | 0.3 | 0.4 | 0.8 | 0.5 | 0.2 | 0.4 | 0.3 | 0.3 | 0.4 | 0.8 | 0.2 | 0.7 | 0.3 | 0.2 | 0.8 | 0.2 |
| 1 | 2826 | 60.2 | 0.3 | 0.8 | 0.1 | 0.1 | 0.2 | 0.2 | 0.1 | 0.7 | 0.3 | 0.3 | 0.3 | 0.3 | 0.4 | 0.8 | 0.5 | 0.2 | 0.4 | 0.3 | 0.3 | 0.4 | 0.8 | 0.2 | 0.6 | 0.3 | 0.2 | 0.8 | 0.2 |
| 1 | 2706 | 69.1 | 0.6 | 0.3 | 0.2 | 0.1 | 0.3 | 0.2 | 0.1 | 0.3 | 0.5 | 0.4 | 0.2 | 0.3 | 0.6 | 0.4 | 0.4 | 0.3 | 0.3 | 0.2 | 0.1 | 0.3 | 0.8 | 0.6 | 0.3 | 0.4 | 0.8 | 0.3 | 0.1 |
| 1 | 2562 | 60.1 | 0.3 | 0.6 | 0.1 | 0.1 | 0.4 | 0.1 | 0.3 | 0.4 | 0.2 | 0.4 | 0.3 | 0.3 | 0.6 | 0.6 | 0.7 | 0.3 | 0.3 | 0.3 | 0.3 | 0.4 | 0.1 | 0.3 | 0.4 | 0.1 | 0.3 | 0.4 | 0.1 |
| 1 | 2592 | 68.7 | 0.5 | 0.3 | 0.1 | 0.3 | 0.3 | 0.3 | 0.1 | 0.3 | 0.5 | 0.3 | 0.3 | 0.3 | 0.3 | 0.5 | 0.3 | 0.3 | 0.3 | 0.3 | 0.6 | 0.3 | 0.5 | 0.3 | 0.3 | 0.3 | 0.6 | 0.3 | 0.1 |
| 1 | 2795 | 70.0 | 0.3 | 0.1 | 0.2 | 0.4 | 0.2 | 0.4 | 0.2 | 0.3 | 0.6 | 0.4 | 0.3 | 0.3 | 0.7 | 0.4 | 0.4 | 0.2 | 0.3 | 0.2 | 0.4 | 0.3 | 0.8 | 0.4 | 0.3 | 0.3 | 0.8 | 0.3 | 0.1 |
| 1 | 2886 | 74.3 | 0.2 | 0.8 | 0.2 | 0.2 | 0.1 | 0.2 | 0.1 | 0.6 | 0.2 | 0.3 | 0.5 | 0.3 | 0.5 | 0.7 | 0.5 | 0.3 | 0.4 | 0.1 | 0.2 | 0.3 | 0.2 | 0.3 | 0.8 | 0.4 | 0.2 | 0.7 | 0.4 |

| | | | | | | | | | | | | | | | | | | | | | | | | | | | | |
|---|---|---|---|---|---|---|---|---|---|---|---|---|---|---|---|---|---|---|---|---|---|---|---|---|---|---|---|---|---|
| 2 | 2752 | 57.9 | 0.8 | 0.1 | 0.2 | 0.3 | 0.4 | 0.2 | 0.1 | 0.5 | 0.1 | 0.3 | 0.8 | 0.3 | 0.8 | 0.5 | 0.4 | 0.4 | 0.2 | 0.1 | 0.1 | 0.3 | 0.5 | 0.2 | 0.2 | 0.3 | 0.7 | 0.3 | 0.1 |
| 2 | 2556 | 71.0 | 0.8 | 0.2 | 0.2 | 0.3 | 0.6 | 0.4 | 0.1 | 0.4 | 0.8 | 0.3 | 0.4 | 0.3 | 0.4 | 0.4 | 0.5 | 0.2 | 0.2 | 0.1 | 0.1 | 0.3 | 0.4 | 0.3 | 0.2 | 0.3 | 0.8 | 0.3 | 0.1 |
| 2 | 2821 | 68.9 | 0.8 | 0.2 | 0.2 | 0.3 | 0.8 | 0.4 | 0.1 | 0.4 | 0.6 | 0.4 | 0.3 | 0.3 | 0.8 | 0.4 | 0.3 | 0.1 | 0.2 | 0.1 | 0.2 | 0.2 | 0.2 | 0.3 | 0.3 | 0.3 | 0.8 | 0.4 | 0.1 |
| 2 | 2710 | 60.4 | 0.8 | 0.3 | 0.5 | 0.3 | 0.4 | 0.8 | 0.1 | 0.4 | 0.5 | 0.3 | 0.3 | 0.3 | 0.3 | 0.5 | 0.3 | 0.3 | 0.1 | 0.1 | 0.4 | 0.3 | 0.5 | 0.3 | 0.2 | 0.3 | 0.7 | 0.3 | 0.1 |
| 2 | 2640 | 64.7 | 0.3 | 0.2 | 0.4 | 0.4 | 0.7 | 0.3 | 0.1 | 0.8 | 0.1 | 0.3 | 0.6 | 0.3 | 0.6 | 0.4 | 0.5 | 0.2 | 0.3 | 0.1 | 0.2 | 0.3 | 0.2 | 0.3 | 0.2 | 0.3 | 0.7 | 0.3 | 0.1 |
| 2 | 2646 | 67.8 | 0.7 | 0.1 | 0.2 | 0.3 | 0.6 | 0.3 | 0.2 | 0.5 | 0.8 | 0.4 | 0.5 | 0.3 | 0.4 | 0.1 | 0.3 | 0.1 | 0.5 | 0.1 | 0.1 | 0.5 | 0.4 | 0.3 | 0.2 | 0.3 | 0.8 | 0.3 | 0.1 |
| 2 | 2732 | 71.1 | 0.8 | 0.2 | 0.1 | 0.3 | 0.8 | 0.4 | 0.1 | 0.4 | 0.6 | 0.3 | 0.3 | 0.3 | 0.8 | 0.4 | 0.3 | 0.1 | 0.2 | 0.1 | 0.2 | 0.2 | 0.4 | 0.3 | 0.3 | 0.3 | 0.8 | 0.4 | 0.1 |
| 2 | 2295 | 63.9 | 0.8 | 0.3 | 0.2 | 0.2 | 0.3 | 0.4 | 0.1 | 0.4 | 0.6 | 0.4 | 0.5 | 0.3 | 0.3 | 0.1 | 0.3 | 0.4 | 0.1 | 0.1 | 0.1 | 0.4 | 0.5 | 0.3 | 0.2 | 0.3 | 0.7 | 0.8 | 0.1 |
| 2 | 2692 | 73.0 | 0.8 | 0.3 | 0.1 | 0.6 | 0.3 | 0.4 | 0.1 | 0.5 | 0.8 | 0.4 | 0.3 | 0.3 | 0.4 | 0.6 | 0.3 | 0.2 | 0.1 | 0.1 | 0.1 | 0.3 | 0.5 | 0.2 | 0.3 | 0.4 | 0.8 | 0.3 | 0.1 |
| 2 | 2739 | 71.0 | 0.7 | 0.3 | 0.3 | 0.5 | 0.8 | 0.3 | 0.1 | 0.4 | 0.6 | 0.3 | 0.5 | 0.5 | 0.2 | 0.4 | 0.3 | 0.2 | 0.2 | 0.1 | 0.2 | 0.4 | 0.2 | 0.3 | 0.2 | 0.2 | 0.8 | 0.1 | 0.2 |
| 2 | 2534 | 71.4 | 0.2 | 0.3 | 0.1 | 0.3 | 0.5 | 0.3 | 0.1 | 0.3 | 0.2 | 0.3 | 0.5 | 0.3 | 0.5 | 0.5 | 0.6 | 0.3 | 0.5 | 0.3 | 0.1 | 0.3 | 0.2 | 0.3 | 0.5 | 0.3 | 0.2 | 0.3 | 0.1 |
| 2 | 2533 | 64.4 | 0.8 | 0.3 | 0.4 | 0.3 | 0.4 | 0.8 | 0.1 | 0.4 | 0.5 | 0.3 | 0.3 | 0.3 | 0.3 | 0.5 | 0.3 | 0.3 | 0.2 | 0.1 | 0.4 | 0.3 | 0.4 | 0.3 | 0.2 | 0.3 | 0.7 | 0.3 | 0.1 |
| 2 | 2666 | 67.7 | 0.8 | 0.2 | 0.4 | 0.4 | 0.8 | 0.3 | 0.1 | 0.2 | 0.6 | 0.2 | 0.7 | 0.3 | 0.4 | 0.3 | 0.3 | 0.4 | 0.1 | 0.1 | 0.1 | 0.3 | 0.4 | 0.4 | 0.2 | 0.2 | 0.8 | 0.3 | 0.1 |
| 2 | 2548 | 66.9 | 0.5 | 0.3 | 0.1 | 0.3 | 0.3 | 0.3 | 0.1 | 0.3 | 0.8 | 0.3 | 0.3 | 0.3 | 0.3 | 0.5 | 0.3 | 0.3 | 0.3 | 0.3 | 0.1 | 0.3 | 0.5 | 0.3 | 0.3 | 0.3 | 0.5 | 0.3 | 0.3 |
| 2 | 2653 | 69.4 | 0.6 | 0.4 | 0.1 | 0.2 | 0.8 | 0.3 | 0.1 | 0.3 | 0.6 | 0.3 | 0.5 | 0.3 | 0.3 | 0.4 | 0.3 | 0.2 | 0.3 | 0.1 | 0.1 | 0.3 | 0.5 | 0.4 | 0.4 | 0.3 | 0.8 | 0.3 | 0.1 |
| 2 | 2634 | 65.2 | 0.3 | 0.2 | 0.1 | 0.5 | 0.4 | 0.3 | 0.1 | 0.5 | 0.6 | 0.4 | 0.8 | 0.3 | 0.3 | 0.5 | 0.3 | 0.3 | 0.1 | 0.1 | 0.1 | 0.3 | 0.4 | 0.4 | 0.1 | 0.3 | 0.7 | 0.3 | 0.1 |
| 2 | 2362 | 76.8 | 0.8 | 0.3 | 0.3 | 0.4 | 0.7 | 0.3 | 0.1 | 0.6 | 0.7 | 0.2 | 0.5 | 0.3 | 0.4 | 0.5 | 0.3 | 0.1 | 0.1 | 0.1 | 0.2 | 0.3 | 0.2 | 0.3 | 0.1 | 0.4 | 0.8 | 0.3 | 0.1 |
| 2 | 2489 | 60.1 | 0.8 | 0.3 | 0.1 | 0.1 | 0.8 | 0.2 | 0.1 | 0.4 | 0.2 | 0.4 | 0.8 | 0.3 | 0.6 | 0.4 | 0.3 | 0.3 | 0.3 | 0.2 | 0.1 | 0.3 | 0.4 | 0.3 | 0.3 | 0.3 | 0.5 | 0.3 | 0.1 |
| 2 | 2561 | 60.7 | 0.4 | 0.2 | 0.1 | 0.3 | 0.6 | 0.2 | 0.1 | 0.5 | 0.2 | 0.3 | 0.5 | 0.4 | 0.6 | 0.7 | 0.4 | 0.4 | 0.5 | 0.1 | 0.2 | 0.3 | 0.2 | 0.2 | 0.3 | 0.3 | 0.6 | 0.3 | 0.1 |
| 2 | 2187 | 70.0 | 0.8 | 0.2 | 0.2 | 0.3 | 0.4 | 0.4 | 0.1 | 0.5 | 0.8 | 0.4 | 0.5 | 0.3 | 0.4 | 0.1 | 0.3 | 0.1 | 0.5 | 0.1 | 0.1 | 0.5 | 0.4 | 0.3 | 0.2 | 0.3 | 0.8 | 0.3 | 0.1 |
| 3 | 2721 | 68.2 | 0.8 | 0.5 | 0.2 | 0.3 | 0.2 | 0.4 | 0.1 | 0.6 | 0.8 | 0.3 | 0.5 | 0.3 | 0.4 | 0.4 | 0.5 | 0.2 | 0.5 | 0.2 | 0.1 | 0.3 | 0.1 | 0.6 | 0.2 | 0.4 | 0.1 | 0.7 | 0.1 |
| 3 | 3050 | 66.6 | 0.8 | 0.5 | 0.4 | 0.4 | 0.3 | 0.2 | 0.1 | 0.8 | 0.7 | 0.4 | 0.6 | 0.1 | 0.5 | 0.4 | 0.7 | 0.1 | 0.7 | 0.2 | 0.1 | 0.2 | 0.1 | 0.4 | 0.2 | 0.2 | 0.1 | 0.3 | 0.1 |
| 3 | 2872 | 64.5 | 0.8 | 0.4 | 0.3 | 0.4 | 0.3 | 0.4 | 0.1 | 0.5 | 0.8 | 0.1 | 0.5 | 0.3 | 0.5 | 0.6 | 0.4 | 0.1 | 0.7 | 0.2 | 0.1 | 0.3 | 0.1 | 0.4 | 0.2 | 0.4 | 0.1 | 0.4 | 0.1 |
| 3 | 2635 | 70.7 | 0.8 | 0.5 | 0.2 | 0.3 | 0.1 | 0.4 | 0.1 | 0.6 | 0.8 | 0.3 | 0.5 | 0.3 | 0.4 | 0.4 | 0.5 | 0.2 | 0.5 | 0.1 | 0.1 | 0.3 | 0.4 | 0.6 | 0.2 | 0.3 | 0.1 | 0.6 | 0.1 |
| 3 | 2917 | 67.0 | 0.7 | 0.5 | 0.2 | 0.3 | 0.1 | 0.4 | 0.1 | 0.6 | 0.8 | 0.3 | 0.5 | 0.3 | 0.4 | 0.4 | 0.5 | 0.2 | 0.5 | 0.1 | 0.1 | 0.3 | 0.4 | 0.6 | 0.2 | 0.3 | 0.1 | 0.6 | 0.1 |
| 3 | 2783 | 58.3 | 0.8 | 0.4 | 0.3 | 0.7 | 0.1 | 0.4 | 0.1 | 0.8 | 0.8 | 0.2 | 0.5 | 0.3 | 0.5 | 0.2 | 0.6 | 0.1 | 0.4 | 0.2 | 0.2 | 0.2 | 0.1 | 0.3 | 0.5 | 0.4 | 0.1 | 0.4 | 0.1 |
| 3 | 2130 | 59.2 | 0.8 | 0.5 | 0.3 | 0.3 | 0.2 | 0.3 | 0.1 | 0.5 | 0.8 | 0.3 | 0.5 | 0.3 | 0.3 | 0.5 | 0.3 | 0.1 | 0.5 | 0.4 | 0.4 | 0.2 | 0.3 | 0.3 | 0.3 | 0.3 | 0.2 | 0.3 | 0.1 |
| 3 | 2650 | 68.2 | 0.8 | 0.6 | 0.3 | 0.4 | 0.2 | 0.4 | 0.1 | 0.8 | 0.8 | 0.3 | 0.5 | 0.3 | 0.4 | 0.2 | 0.7 | 0.1 | 0.1 | 0.3 | 0.1 | 0.3 | 0.2 | 0.4 | 0.5 | 0.3 | 0.1 | 0.6 | 0.1 |
| 3 | 2904 | 61.4 | 0.8 | 0.4 | 0.3 | 0.3 | 0.2 | 0.4 | 0.1 | 0.8 | 0.8 | 0.1 | 0.4 | 0.3 | 0.5 | 0.5 | 0.8 | 0.1 | 0.5 | 0.3 | 0.2 | 0.4 | 0.1 | 0.2 | 0.1 | 0.4 | 0.2 | 0.5 | 0.1 |
| 3 | 2533 | 72.0 | 0.3 | 0.3 | 0.1 | 0.3 | 0.5 | 0.3 | 0.1 | 0.3 | 0.2 | 0.3 | 0.5 | 0.3 | 0.5 | 0.5 | 0.6 | 0.3 | 0.5 | 0.3 | 0.1 | 0.3 | 0.2 | 0.3 | 0.5 | 0.3 | 0.2 | 0.3 | 0.1 |
| 3 | 2667 | 65.4 | 0.8 | 0.6 | 0.3 | 0.4 | 0.2 | 0.4 | 0.1 | 0.6 | 0.8 | 0.3 | 0.5 | 0.3 | 0.4 | 0.2 | 0.7 | 0.1 | 0.1 | 0.3 | 0.1 | 0.3 | 0.2 | 0.4 | 0.5 | 0.3 | 0.1 | 0.6 | 0.1 |
| 3 | 2651 | 66.5 | 0.8 | 0.4 | 0.1 | 0.4 | 0.3 | 0.2 | 0.1 | 0.6 | 0.3 | 0.1 | 0.7 | 0.3 | 0.5 | 0.6 | 0.6 | 0.3 | 0.5 | 0.1 | 0.1 | 0.2 | 0.2 | 0.3 | 0.5 | 0.3 | 0.2 | 0.4 | 0.1 |
| 3 | 2894 | 61.4 | 0.8 | 0.4 | 0.3 | 0.3 | 0.2 | 0.3 | 0.1 | 0.8 | 0.8 | 0.1 | 0.3 | 0.3 | 0.5 | 0.5 | 0.8 | 0.2 | 0.5 | 0.3 | 0.2 | 0.4 | 0.1 | 0.2 | 0.1 | 0.4 | 0.2 | 0.4 | 0.1 |
| 3 | 2358 | 62.5 | 0.8 | 0.6 | 0.2 | 0.4 | 0.1 | 0.3 | 0.1 | 0.8 | 0.8 | 0.2 | 0.4 | 0.3 | 0.4 | 0.5 | 0.4 | 0.1 | 0.8 | 0.3 | 0.6 | 0.2 | 0.1 | 0.4 | 0.4 | 0.3 | 0.2 | 0.1 | 0.1 |
| 3 | 2580 | 58.7 | 0.8 | 0.4 | 0.3 | 0.4 | 0.1 | 0.3 | 0.1 | 0.8 | 0.8 | 0.3 | 0.5 | 0.3 | 0.4 | 0.4 | 0.7 | 0.1 | 0.6 | 0.2 | 0.3 | 0.3 | 0.1 | 0.3 | 0.2 | 0.3 | 0.1 | 0.5 | 0.1 |
| 3 | 2553 | 61.0 | 0.8 | 0.5 | 0.3 | 0.7 | 0.1 | 0.2 | 0.1 | 0.7 | 0.8 | 0.3 | 0.5 | 0.3 | 0.5 | 0.5 | 0.3 | 0.1 | 0.7 | 0.3 | 0.4 | 0.3 | 0.1 | 0.4 | 0.1 | 0.4 | 0.1 | 0.1 | 0.1 |
| 3 | 2677 | 64.1 | 0.8 | 0.4 | 0.4 | 0.3 | 0.2 | 0.2 | 0.1 | 0.8 | 0.8 | 0.2 | 0.5 | 0.2 | 0.3 | 0.4 | 0.4 | 0.3 | 0.3 | 0.3 | 0.7 | 0.3 | 0.1 | 0.3 | 0.4 | 0.4 | 0.1 | 0.2 | 0.3 |
| 3 | 2042 | 60.6 | 0.8 | 0.6 | 0.1 | 0.4 | 0.3 | 0.4 | 0.1 | 0.6 | 0.8 | 0.2 | 0.3 | 0.2 | 0.4 | 0.5 | 0.3 | 0.1 | 0.8 | 0.3 | 0.6 | 0.3 | 0.1 | 0.4 | 0.4 | 0.3 | 0.3 | 0.2 | 0.1 |
| 3 | 2783 | 58.3 | 0.8 | 0.4 | 0.3 | 0.7 | 0.1 | 0.4 | 0.1 | 0.8 | 0.8 | 0.2 | 0.5 | 0.3 | 0.5 | 0.2 | 0.6 | 0.1 | 0.4 | 0.2 | 0.2 | 0.2 | 0.1 | 0.3 | 0.5 | 0.4 | 0.1 | 0.4 | 0.1 |
| 3 | 2812 | 59.5 | 0.8 | 0.5 | 0.3 | 0.2 | 0.1 | 0.3 | 0.1 | 0.8 | 0.8 | 0.2 | 0.5 | 0.3 | 0.5 | 0.5 | 0.6 | 0.1 | 0.4 | 0.3 | 0.6 | 0.3 | 0.1 | 0.3 | 0.5 | 0.2 | 0.1 | 0.1 | 0.1 |
| 4 | 2574 | 73.2 | 0.3 | 0.3 | 0.1 | 0.4 | 0.5 | 0.3 | 0.1 | 0.3 | 0.2 | 0.3 | 0.5 | 0.3 | 0.5 | 0.5 | 0.6 | 0.3 | 0.4 | 0.3 | 0.1 | 0.3 | 0.2 | 0.3 | 0.5 | 0.3 | 0.2 | 0.3 | 0.1 |

| | | | | | | | | | | | | | | | | | | | | | | | | | | | | | |
|---|---|---|---|---|---|---|---|---|---|---|---|---|---|---|---|---|---|---|---|---|---|---|---|---|---|---|---|---|---|
| 4 | 3009 | 73.5 | 0.2 | 0.7 | 0.1 | 0.4 | 0.5 | 0.3 | 0.1 | 0.3 | 0.2 | 0.3 | 0.3 | 0.3 | 0.5 | 0.5 | 0.6 | 0.1 | 0.7 | 0.3 | 0.3 | 0.3 | 0.2 | 0.4 | 0.5 | 0.3 | 0.8 | 0.5 | 0.1 |
| 4 | 3037 | 77.3 | 0.8 | 0.6 | 0.2 | 0.2 | 0.2 | 0.1 | 0.1 | 0.2 | 0.7 | 0.2 | 0.4 | 0.3 | 0.4 | 0.5 | 0.5 | 0.1 | 0.7 | 0.3 | 0.4 | 0.4 | 0.4 | 0.4 | 0.2 | 0.4 | 0.8 | 0.3 | 0.1 |
| 4 | 2967 | 77.7 | 0.8 | 0.4 | 0.4 | 0.4 | 0.6 | 0.3 | 0.1 | 0.5 | 0.8 | 0.2 | 0.4 | 0.3 | 0.4 | 0.5 | 0.2 | 0.1 | 0.4 | 0.3 | 0.4 | 0.4 | 0.2 | 0.3 | 0.1 | 0.4 | 0.8 | 0.3 | 0.1 |
| 4 | 3261 | 77.6 | 0.8 | 0.7 | 0.3 | 0.4 | 0.6 | 0.4 | 0.1 | 0.6 | 0.7 | 0.3 | 0.5 | 0.3 | 0.3 | 0.5 | 0.1 | 0.1 | 0.1 | 0.5 | 0.1 | 0.2 | 0.2 | 0.4 | 0.2 | 0.4 | 0.8 | 0.6 | 0.1 |
| 4 | 3373 | 78.6 | 0.8 | 0.6 | 0.2 | 0.3 | 0.4 | 0.1 | 0.3 | 0.2 | 0.7 | 0.2 | 0.4 | 0.2 | 0.2 | 0.4 | 0.6 | 0.1 | 0.8 | 0.8 | 0.1 | 0.3 | 0.2 | 0.4 | 0.1 | 0.3 | 0.8 | 0.5 | 0.1 |
| 4 | 3010 | 78.7 | 0.3 | 0.6 | 0.1 | 0.5 | 0.5 | 0.1 | 0.1 | 0.2 | 0.2 | 0.3 | 0.5 | 0.3 | 0.5 | 0.5 | 0.6 | 0.1 | 0.6 | 0.4 | 0.4 | 0.3 | 0.2 | 0.4 | 0.3 | 0.3 | 0.8 | 0.3 | 0.2 |
| 4 | 3004 | 82.1 | 0.8 | 0.5 | 0.1 | 0.4 | 0.5 | 0.3 | 0.1 | 0.3 | 0.4 | 0.3 | 0.5 | 0.3 | 0.4 | 0.4 | 0.5 | 0.1 | 0.8 | 0.4 | 0.4 | 0.2 | 0.4 | 0.4 | 0.3 | 0.2 | 0.8 | 0.3 | 0.2 |
| 4 | 2918 | 73.1 | 0.5 | 0.3 | 0.1 | 0.3 | 0.5 | 0.4 | 0.1 | 0.3 | 0.1 | 0.3 | 0.4 | 0.3 | 0.3 | 0.5 | 0.5 | 0.1 | 0.8 | 0.4 | 0.1 | 0.3 | 0.3 | 0.7 | 0.2 | 0.3 | 0.7 | 0.6 | 0.1 |
| 4 | 2904 | 78.8 | 0.8 | 0.5 | 0.3 | 0.4 | 0.6 | 0.3 | 0.1 | 0.2 | 0.6 | 0.1 | 0.5 | 0.3 | 0.4 | 0.5 | 0.3 | 0.1 | 0.5 | 0.4 | 0.2 | 0.3 | 0.2 | 0.5 | 0.2 | 0.4 | 0.8 | 0.3 | 0.1 |
| 4 | 2993 | 73.1 | 0.8 | 0.6 | 0.2 | 0.4 | 0.2 | 0.3 | 0.2 | 0.2 | 0.7 | 0.2 | 0.5 | 0.2 | 0.4 | 0.3 | 0.5 | 0.1 | 0.8 | 0.3 | 0.2 | 0.3 | 0.4 | 0.4 | 0.3 | 0.3 | 0.8 | 0.6 | 0.1 |
| 4 | 2941 | 70.3 | 0.8 | 0.5 | 0.3 | 0.4 | 0.6 | 0.1 | 0.1 | 0.3 | 0.6 | 0.3 | 0.4 | 0.3 | 0.4 | 0.5 | 0.2 | 0.1 | 0.6 | 0.4 | 0.4 | 0.3 | 0.2 | 0.5 | 0.2 | 0.4 | 0.8 | 0.3 | 0.2 |
| 4 | 2709 | 77.7 | 0.2 | 0.4 | 0.1 | 0.4 | 0.5 | 0.3 | 0.1 | 0.3 | 0.2 | 0.3 | 0.3 | 0.3 | 0.3 | 0.7 | 0.6 | 0.1 | 0.8 | 0.4 | 0.4 | 0.3 | 0.2 | 0.4 | 0.3 | 0.3 | 0.8 | 0.6 | 0.1 |
| 4 | 2859 | 69.3 | 0.8 | 0.5 | 0.2 | 0.4 | 0.2 | 0.3 | 0.1 | 0.2 | 0.7 | 0.3 | 0.5 | 0.2 | 0.4 | 0.3 | 0.5 | 0.1 | 0.7 | 0.2 | 0.3 | 0.3 | 0.4 | 0.4 | 0.3 | 0.3 | 0.8 | 0.6 | 0.1 |
| 4 | 3004 | 81.0 | 0.4 | 0.6 | 0.1 | 0.4 | 0.5 | 0.1 | 0.1 | 0.2 | 0.2 | 0.3 | 0.5 | 0.3 | 0.5 | 0.5 | 0.6 | 0.1 | 0.7 | 0.4 | 0.4 | 0.3 | 0.2 | 0.4 | 0.3 | 0.3 | 0.8 | 0.3 | 0.2 |
| 4 | 2858 | 73.2 | 0.8 | 0.5 | 0.2 | 0.2 | 0.2 | 0.2 | 0.1 | 0.5 | 0.7 | 0.3 | 0.8 | 0.3 | 0.4 | 0.4 | 0.5 | 0.2 | 0.3 | 0.2 | 0.2 | 0.3 | 0.4 | 0.3 | 0.2 | 0.4 | 0.8 | 0.3 | 0.1 |
| 4 | 2786 | 74.5 | 0.2 | 0.4 | 0.2 | 0.4 | 0.5 | 0.2 | 0.1 | 0.2 | 0.2 | 0.3 | 0.3 | 0.3 | 0.5 | 0.6 | 0.4 | 0.1 | 0.8 | 0.5 | 0.4 | 0.2 | 0.2 | 0.4 | 0.4 | 0.3 | 0.8 | 0.6 | 0.1 |
| 4 | 3026 | 79.0 | 0.2 | 0.7 | 0.1 | 0.4 | 0.5 | 0.3 | 0.2 | 0.2 | 0.2 | 0.3 | 0.5 | 0.3 | 0.5 | 0.5 | 0.6 | 0.1 | 0.8 | 0.3 | 0.2 | 0.3 | 0.2 | 0.3 | 0.5 | 0.3 | 0.8 | 0.3 | 0.1 |
| 4 | 3024 | 74.6 | 0.8 | 0.6 | 0.1 | 0.3 | 0.5 | 0.3 | 0.2 | 0.3 | 0.8 | 0.3 | 0.5 | 0.3 | 0.3 | 0.4 | 0.5 | 0.1 | 0.8 | 0.2 | 0.4 | 0.2 | 0.4 | 0.4 | 0.3 | 0.3 | 0.8 | 0.3 | 0.1 |
| 4 | 2741 | 73.6 | 0.8 | 0.7 | 0.2 | 0.4 | 0.5 | 0.1 | 0.1 | 0.3 | 0.8 | 0.3 | 0.3 | 0.3 | 0.4 | 0.5 | 0.5 | 0.1 | 0.7 | 0.2 | 0.3 | 0.3 | 0.3 | 0.6 | 0.2 | 0.3 | 0.8 | 0.1 | 0.3 |
| 5 | 3180 | 63.9 | 0.1 | 0.4 | 0.3 | 0.4 | 0.6 | 0.5 | 0.1 | 0.1 | 0.1 | 0.3 | 0.5 | 0.2 | 0.5 | 0.7 | 0.8 | 0.3 | 0.4 | 0.2 | 0.1 | 0.3 | 0.4 | 0.3 | 0.1 | 0.2 | 0.2 | 0.3 | 0.5 |
| 5 | 2988 | 69.3 | 0.1 | 0.5 | 0.1 | 0.4 | 0.8 | 0.5 | 0.1 | 0.2 | 0.1 | 0.3 | 0.5 | 0.3 | 0.5 | 0.7 | 0.7 | 0.1 | 0.4 | 0.3 | 0.1 | 0.3 | 0.2 | 0.4 | 0.3 | 0.3 | 0.3 | 0.1 | 0.5 |
| 5 | 2830 | 63.4 | 0.1 | 0.4 | 0.2 | 0.4 | 0.8 | 0.4 | 0.1 | 0.2 | 0.1 | 0.3 | 0.5 | 0.3 | 0.4 | 0.6 | 0.6 | 0.2 | 0.5 | 0.3 | 0.1 | 0.3 | 0.2 | 0.4 | 0.4 | 0.3 | 0.2 | 0.2 | 0.4 |
| 5 | 2814 | 68.2 | 0.1 | 0.3 | 0.1 | 0.4 | 0.5 | 0.5 | 0.1 | 0.1 | 0.1 | 0.1 | 0.5 | 0.3 | 0.5 | 0.6 | 0.6 | 0.3 | 0.5 | 0.3 | 0.3 | 0.6 | 0.3 | 0.4 | 0.1 | 0.3 | 0.2 | 0.1 | 0.4 |
| 5 | 2619 | 74.6 | 0.6 | 0.3 | 0.2 | 0.3 | 0.7 | 0.5 | 0.1 | 0.6 | 0.3 | 0.2 | 0.4 | 0.3 | 0.4 | 0.5 | 0.5 | 0.1 | 0.1 | 0.2 | 0.1 | 0.3 | 0.4 | 0.4 | 0.2 | 0.3 | 0.8 | 0.3 | 0.1 |
| 5 | 2780 | 76.9 | 0.1 | 0.3 | 0.1 | 0.4 | 0.5 | 0.4 | 0.1 | 0.3 | 0.2 | 0.2 | 0.5 | 0.3 | 0.5 | 0.7 | 0.6 | 0.3 | 0.3 | 0.4 | 0.4 | 0.3 | 0.2 | 0.4 | 0.3 | 0.2 | 0.2 | 0.2 | 0.4 |
| 5 | 3008 | 63.4 | 0.2 | 0.4 | 0.2 | 0.4 | 0.5 | 0.6 | 0.1 | 0.3 | 0.1 | 0.3 | 0.4 | 0.2 | 0.2 | 0.5 | 0.7 | 0.4 | 0.5 | 0.3 | 0.1 | 0.3 | 0.2 | 0.3 | 0.4 | 0.3 | 0.3 | 0.1 | 0.8 |
| 5 | 2761 | 74.5 | 0.1 | 0.3 | 0.1 | 0.4 | 0.5 | 0.4 | 0.1 | 0.2 | 0.2 | 0.2 | 0.5 | 0.3 | 0.5 | 0.7 | 0.6 | 0.3 | 0.3 | 0.4 | 0.4 | 0.3 | 0.2 | 0.4 | 0.3 | 0.2 | 0.2 | 0.2 | 0.4 |
| 5 | 2705 | 73.4 | 0.1 | 0.3 | 0.1 | 0.4 | 0.5 | 0.4 | 0.1 | 0.3 | 0.2 | 0.2 | 0.5 | 0.3 | 0.5 | 0.6 | 0.6 | 0.3 | 0.3 | 0.4 | 0.4 | 0.3 | 0.2 | 0.4 | 0.3 | 0.2 | 0.2 | 0.2 | 0.4 |
| 5 | 2797 | 67.9 | 0.1 | 0.5 | 0.4 | 0.4 | 0.5 | 0.5 | 0.1 | 0.2 | 0.1 | 0.3 | 0.6 | 0.3 | 0.4 | 0.6 | 0.6 | 0.2 | 0.2 | 0.2 | 0.1 | 0.5 | 0.2 | 0.4 | 0.2 | 0.3 | 0.2 | 0.3 | 0.5 |
| 5 | 2883 | 68.6 | 0.3 | 0.4 | 0.1 | 0.4 | 0.6 | 0.4 | 0.1 | 0.2 | 0.1 | 0.3 | 0.5 | 0.3 | 0.4 | 0.7 | 0.6 | 0.2 | 0.4 | 0.4 | 0.1 | 0.3 | 0.2 | 0.4 | 0.2 | 0.3 | 0.2 | 0.2 | 0.4 |
| 5 | 2522 | 63.5 | 0.7 | 0.3 | 0.3 | 0.4 | 0.6 | 0.3 | 0.1 | 0.6 | 0.5 | 0.1 | 0.5 | 0.3 | 0.3 | 0.5 | 0.3 | 0.1 | 0.1 | 0.1 | 0.1 | 0.5 | 0.2 | 0.4 | 0.1 | 0.4 | 0.8 | 0.3 | 0.2 |
| 5 | 2327 | 59.2 | 0.3 | 0.3 | 0.1 | 0.4 | 0.3 | 0.1 | 0.1 | 0.4 | 0.4 | 0.1 | 0.5 | 0.3 | 0.4 | 0.6 | 0.5 | 0.1 | 0.1 | 0.2 | 0.1 | 0.6 | 0.4 | 0.4 | 0.1 | 0.3 | 0.8 | 0.3 | 0.4 |
| 5 | 2575 | 64.0 | 0.5 | 0.3 | 0.1 | 0.4 | 0.5 | 0.2 | 0.1 | 0.5 | 0.5 | 0.2 | 0.5 | 0.3 | 0.3 | 0.7 | 0.3 | 0.1 | 0.1 | 0.2 | 0.2 | 0.6 | 0.2 | 0.4 | 0.1 | 0.4 | 0.8 | 0.3 | 0.3 |
| 5 | 3030 | 60.0 | 0.1 | 0.5 | 0.1 | 0.4 | 0.7 | 0.5 | 0.1 | 0.3 | 0.1 | 0.3 | 0.4 | 0.2 | 0.4 | 0.6 | 0.8 | 0.2 | 0.3 | 0.1 | 0.2 | 0.3 | 0.4 | 0.4 | 0.1 | 0.2 | 0.2 | 0.7 | 0.8 |
| 5 | 2898 | 73.0 | 0.1 | 0.5 | 0.1 | 0.4 | 0.7 | 0.5 | 0.1 | 0.2 | 0.1 | 0.3 | 0.5 | 0.3 | 0.5 | 0.7 | 0.7 | 0.1 | 0.4 | 0.3 | 0.2 | 0.3 | 0.2 | 0.4 | 0.3 | 0.3 | 0.3 | 0.1 | 0.5 |
| 5 | 2499 | 65.3 | 0.6 | 0.1 | 0.2 | 0.4 | 0.2 | 0.4 | 0.1 | 0.7 | 0.5 | 0.1 | 0.4 | 0.1 | 0.4 | 0.6 | 0.5 | 0.1 | 0.1 | 0.2 | 0.3 | 0.6 | 0.4 | 0.3 | 0.2 | 0.3 | 0.8 | 0.3 | 0.1 |
| 5 | 2309 | 70.5 | 0.8 | 0.3 | 0.2 | 0.4 | 0.7 | 0.3 | 0.1 | 0.7 | 0.6 | 0.2 | 0.5 | 0.3 | 0.4 | 0.5 | 0.3 | 0.1 | 0.1 | 0.3 | 0.2 | 0.4 | 0.2 | 0.3 | 0.1 | 0.4 | 0.8 | 0.2 | 0.1 |
| 5 | 2879 | 66.3 | 0.1 | 0.6 | 0.2 | 0.4 | 0.5 | 0.5 | 0.1 | 0.1 | 0.1 | 0.3 | 0.5 | 0.3 | 0.2 | 0.7 | 0.7 | 0.4 | 0.4 | 0.2 | 0.2 | 0.2 | 0.3 | 0.4 | 0.3 | 0.3 | 0.2 | 0.2 | 0.6 |
| 5 | 2489 | 76.5 | 0.8 | 0.3 | 0.3 | 0.4 | 0.7 | 0.3 | 0.1 | 0.6 | 0.6 | 0.2 | 0.5 | 0.3 | 0.4 | 0.5 | 0.3 | 0.1 | 0.1 | 0.2 | 0.3 | 0.4 | 0.2 | 0.3 | 0.1 | 0.4 | 0.8 | 0.3 | 0.1 |
| 6 | 2584 | 70.0 | 0.2 | 0.4 | 0.1 | 0.2 | 0.5 | 0.3 | 0.1 | 0.3 | 0.3 | 0.3 | 0.5 | 0.3 | 0.5 | 0.7 | 0.6 | 0.1 | 0.8 | 0.3 | 0.8 | 0.3 | 0.2 | 0.4 | 0.3 | 0.3 | 0.4 | 0.1 | 0.1 |
| 6 | 2549 | 70.7 | 0.8 | 0.3 | 0.2 | 0.4 | 0.6 | 0.3 | 0.1 | 0.7 | 0.8 | 0.2 | 0.5 | 0.3 | 0.4 | 0.6 | 0.5 | 0.1 | 0.1 | 0.3 | 0.3 | 0.3 | 0.3 | 0.4 | 0.2 | 0.3 | 0.8 | 0.1 | 0.1 |

| | | | | | | | | | | | | | | | | | | | | | | | | | | | | | |
|---|---|---|---|---|---|---|---|---|---|---|---|---|---|---|---|---|---|---|---|---|---|---|---|---|---|---|---|---|---|
| 6 | 2696 | 77.0 | 0.8 | 0.3 | 0.3 | 0.4 | 0.8 | 0.3 | 0.1 | 0.6 | 0.7 | 0.2 | 0.5 | 0.4 | 0.4 | 0.5 | 0.3 | 0.1 | 0.1 | 0.2 | 0.3 | 0.3 | 0.2 | 0.3 | 0.1 | 0.4 | 0.8 | 0.3 | 0.1 |
| 6 | 2841 | 72.3 | 0.7 | 0.3 | 0.3 | 0.4 | 0.5 | 0.4 | 0.1 | 0.7 | 0.6 | 0.2 | 0.5 | 0.3 | 0.4 | 0.5 | 0.4 | 0.1 | 0.1 | 0.3 | 0.3 | 0.3 | 0.3 | 0.3 | 0.1 | 0.5 | 0.7 | 0.2 | 0.1 |
| 6 | 2656 | 76.6 | 0.8 | 0.3 | 0.2 | 0.1 | 0.8 | 0.3 | 0.1 | 0.8 | 0.8 | 0.2 | 0.6 | 0.3 | 0.5 | 0.4 | 0.3 | 0.2 | 0.1 | 0.2 | 0.3 | 0.3 | 0.6 | 0.3 | 0.2 | 0.4 | 0.8 | 0.2 | 0.1 |
| 6 | 2718 | 67.4 | 0.8 | 0.3 | 0.1 | 0.2 | 0.7 | 0.4 | 0.1 | 0.7 | 0.7 | 0.1 | 0.4 | 0.4 | 0.4 | 0.4 | 0.5 | 0.1 | 0.1 | 0.2 | 0.2 | 0.4 | 0.4 | 0.4 | 0.3 | 0.3 | 0.8 | 0.2 | 0.1 |
| 6 | 2795 | 67.3 | 0.8 | 0.4 | 0.1 | 0.2 | 0.7 | 0.4 | 0.1 | 0.7 | 0.7 | 0.1 | 0.4 | 0.4 | 0.4 | 0.4 | 0.5 | 0.1 | 0.1 | 0.2 | 0.3 | 0.4 | 0.3 | 0.4 | 0.3 | 0.3 | 0.8 | 0.2 | 0.1 |
| 6 | 2474 | 72.1 | 0.8 | 0.2 | 0.2 | 0.4 | 0.6 | 0.3 | 0.1 | 0.7 | 0.8 | 0.2 | 0.5 | 0.3 | 0.4 | 0.6 | 0.5 | 0.1 | 0.1 | 0.3 | 0.3 | 0.3 | 0.3 | 0.4 | 0.3 | 0.3 | 0.8 | 0.1 | 0.1 |
| 6 | 2843 | 77.8 | 0.8 | 0.4 | 0.1 | 0.3 | 0.8 | 0.5 | 0.1 | 0.6 | 0.2 | 0.2 | 0.4 | 0.4 | 0.4 | 0.5 | 0.5 | 0.1 | 0.1 | 0.2 | 0.4 | 0.3 | 0.3 | 0.4 | 0.2 | 0.4 | 0.5 | 0.3 | 0.1 |
| 6 | 2652 | 78.7 | 0.8 | 0.3 | 0.3 | 0.4 | 0.8 | 0.3 | 0.1 | 0.6 | 0.6 | 0.2 | 0.5 | 0.4 | 0.4 | 0.5 | 0.3 | 0.1 | 0.1 | 0.2 | 0.3 | 0.3 | 0.2 | 0.3 | 0.1 | 0.4 | 0.8 | 0.3 | 0.1 |
| 6 | 3060 | 69.5 | 0.7 | 0.3 | 0.3 | 0.3 | 0.7 | 0.4 | 0.1 | 0.6 | 0.7 | 0.2 | 0.4 | 0.3 | 0.5 | 0.1 | 0.3 | 0.1 | 0.1 | 0.2 | 0.2 | 0.4 | 0.3 | 0.3 | 0.5 | 0.4 | 0.7 | 0.3 | 0.1 |
| 6 | 2598 | 72.8 | 0.8 | 0.5 | 0.2 | 0.3 | 0.6 | 0.4 | 0.1 | 0.5 | 0.5 | 0.2 | 0.4 | 0.3 | 0.2 | 0.5 | 0.5 | 0.1 | 0.1 | 0.2 | 0.2 | 0.3 | 0.4 | 0.4 | 0.3 | 0.3 | 0.7 | 0.3 | 0.1 |
| 6 | 2685 | 79.9 | 0.8 | 0.3 | 0.3 | 0.4 | 0.7 | 0.3 | 0.1 | 0.7 | 0.7 | 0.2 | 0.5 | 0.3 | 0.4 | 0.5 | 0.4 | 0.1 | 0.1 | 0.2 | 0.2 | 0.3 | 0.2 | 0.3 | 0.2 | 0.4 | 0.8 | 0.3 | 0.1 |
| 6 | 2721 | 67.1 | 0.3 | 0.3 | 0.1 | 0.3 | 0.4 | 0.3 | 0.1 | 0.3 | 0.2 | 0.3 | 0.5 | 0.3 | 0.5 | 0.5 | 0.6 | 0.3 | 0.5 | 0.3 | 0.1 | 0.3 | 0.2 | 0.3 | 0.5 | 0.3 | 0.2 | 0.3 | 0.1 |
| 6 | 2711 | 74.4 | 0.2 | 0.5 | 0.1 | 0.3 | 0.5 | 0.4 | 0.3 | 0.3 | 0.3 | 0.3 | 0.6 | 0.3 | 0.5 | 0.7 | 0.6 | 0.1 | 0.2 | 0.3 | 0.8 | 0.2 | 0.1 | 0.3 | 0.5 | 0.3 | 0.3 | 0.2 | 0.1 |
| 6 | 2689 | 74.6 | 0.8 | 0.3 | 0.3 | 0.3 | 0.7 | 0.4 | 0.1 | 0.6 | 0.7 | 0.1 | 0.4 | 0.4 | 0.4 | 0.1 | 0.3 | 0.1 | 0.1 | 0.2 | 0.2 | 0.4 | 0.3 | 0.3 | 0.5 | 0.4 | 0.8 | 0.3 | 0.1 |
| 6 | 2625 | 68.4 | 0.3 | 0.4 | 0.1 | 0.3 | 0.5 | 0.3 | 0.1 | 0.1 | 0.3 | 0.2 | 0.5 | 0.3 | 0.5 | 0.5 | 0.6 | 0.1 | 0.5 | 0.3 | 0.4 | 0.3 | 0.2 | 0.3 | 0.4 | 0.3 | 0.3 | 0.2 | 0.2 |
| 6 | 3214 | 72.4 | 0.1 | 0.4 | 0.2 | 0.2 | 0.5 | 0.3 | 0.4 | 0.2 | 0.1 | 0.2 | 0.5 | 0.2 | 0.7 | 0.7 | 0.6 | 0.3 | 0.3 | 0.3 | 0.8 | 0.2 | 0.1 | 0.2 | 0.5 | 0.6 | 0.2 | 0.1 | 0.1 |
| 6 | 2507 | 72.9 | 0.8 | 0.3 | 0.2 | 0.3 | 0.3 | 0.3 | 0.1 | 0.6 | 0.6 | 0.2 | 0.5 | 0.3 | 0.4 | 0.4 | 0.5 | 0.1 | 0.1 | 0.4 | 0.2 | 0.3 | 0.4 | 0.4 | 0.2 | 0.3 | 0.4 | 0.7 | 0.1 |
| 6 | 2507 | 72.9 | 0.8 | 0.3 | 0.2 | 0.3 | 0.3 | 0.3 | 0.1 | 0.6 | 0.6 | 0.2 | 0.5 | 0.3 | 0.4 | 0.4 | 0.5 | 0.1 | 0.1 | 0.4 | 0.2 | 0.3 | 0.4 | 0.4 | 0.2 | 0.3 | 0.4 | 0.7 | 0.1 |
| 7 | 2776 | 79.8 | 0.8 | 0.2 | 0.2 | 0.4 | 0.3 | 0.1 | 0.2 | 0.6 | 0.5 | 0.3 | 0.5 | 0.3 | 0.4 | 0.6 | 0.5 | 0.1 | 0.1 | 0.4 | 0.3 | 0.2 | 0.5 | 0.3 | 0.2 | 0.3 | 0.5 | 0.7 | 0.1 |
| 7 | 2531 | 73.1 | 0.8 | 0.2 | 0.1 | 0.3 | 0.7 | 0.3 | 0.1 | 0.6 | 0.2 | 0.4 | 0.3 | 0.3 | 0.5 | 0.5 | 0.5 | 0.1 | 0.2 | 0.4 | 0.3 | 0.3 | 0.5 | 0.3 | 0.2 | 0.3 | 0.8 | 0.3 | 0.1 |
| 7 | 2667 | 69.4 | 0.8 | 0.3 | 0.1 | 0.5 | 0.8 | 0.3 | 0.1 | 0.6 | 0.2 | 0.4 | 0.4 | 0.2 | 0.4 | 0.5 | 0.5 | 0.1 | 0.1 | 0.4 | 0.3 | 0.3 | 0.5 | 0.2 | 0.2 | 0.3 | 0.8 | 0.3 | 0.1 |
| 7 | 2825 | 72.9 | 0.8 | 0.3 | 0.2 | 0.4 | 0.7 | 0.1 | 0.1 | 0.6 | 0.2 | 0.5 | 0.5 | 0.3 | 0.4 | 0.6 | 0.6 | 0.3 | 0.4 | 0.4 | 0.7 | 0.1 | 0.1 | 0.3 | 0.4 | 0.3 | 0.2 | 0.3 | 0.1 |
| 7 | 2681 | 76.6 | 0.8 | 0.3 | 0.1 | 0.4 | 0.6 | 0.3 | 0.1 | 0.6 | 0.2 | 0.3 | 0.5 | 0.2 | 0.4 | 0.8 | 0.5 | 0.1 | 0.1 | 0.4 | 0.6 | 0.3 | 0.4 | 0.3 | 0.2 | 0.3 | 0.4 | 0.5 | 0.1 |
| 7 | 2963 | 72.4 | 0.8 | 0.2 | 0.1 | 0.6 | 0.7 | 0.3 | 0.1 | 0.6 | 0.2 | 0.3 | 0.5 | 0.3 | 0.5 | 0.7 | 0.6 | 0.3 | 0.1 | 0.3 | 0.4 | 0.2 | 0.1 | 0.3 | 0.3 | 0.3 | 0.1 | 0.6 | 0.1 |
| 7 | 2701 | 81.2 | 0.8 | 0.2 | 0.1 | 0.4 | 0.7 | 0.3 | 0.1 | 0.5 | 0.3 | 0.2 | 0.5 | 0.3 | 0.4 | 0.7 | 0.5 | 0.1 | 0.1 | 0.4 | 0.4 | 0.2 | 0.4 | 0.3 | 0.2 | 0.3 | 0.5 | 0.6 | 0.1 |
| 7 | 2511 | 78.4 | 0.8 | 0.2 | 0.3 | 0.4 | 0.7 | 0.3 | 0.1 | 0.5 | 0.5 | 0.2 | 0.5 | 0.3 | 0.4 | 0.5 | 0.4 | 0.1 | 0.1 | 0.3 | 0.2 | 0.2 | 0.2 | 0.3 | 0.1 | 0.5 | 0.8 | 0.4 | 0.1 |
| 7* DANTE #2 | 2582 | 79.9 | 0.8 | 0.3 | 0.1 | 0.4 | 0.7 | 0.3 | 0.1 | 0.6 | 0.3 | 0.4 | 0.5 | 0.4 | 0.3 | 0.6 | 0.5 | 0.1 | 0.2 | 0.3 | 0.3 | 0.2 | 0.3 | 0.3 | 0.2 | 0.3 | 0.8 | 0.3 | 0.1 |
| 7 | 2557 | 76.9 | 0.8 | 0.2 | 0.3 | 0.4 | 0.7 | 0.2 | 0.1 | 0.6 | 0.8 | 0.3 | 0.6 | 0.3 | 0.4 | 0.5 | 0.3 | 0.1 | 0.1 | 0.3 | 0.3 | 0.3 | 0.2 | 0.2 | 0.2 | 0.4 | 0.8 | 0.4 | 0.1 |
| 7 | 2652 | 77.7 | 0.8 | 0.2 | 0.1 | 0.4 | 0.7 | 0.3 | 0.1 | 0.5 | 0.6 | 0.4 | 0.5 | 0.4 | 0.4 | 0.5 | 0.3 | 0.1 | 0.2 | 0.4 | 0.2 | 0.2 | 0.2 | 0.2 | 0.3 | 0.4 | 0.6 | 0.3 | 0.1 |
| 7 | 2732 | 77.7 | 0.8 | 0.3 | 0.2 | 0.4 | 0.7 | 0.5 | 0.1 | 0.6 | 0.3 | 0.2 | 0.5 | 0.3 | 0.4 | 0.5 | 0.5 | 0.2 | 0.1 | 0.2 | 0.1 | 0.3 | 0.4 | 0.3 | 0.2 | 0.3 | 0.8 | 0.3 | 0.1 |
| 7 | 2626 | 76.0 | 0.8 | 0.2 | 0.2 | 0.3 | 0.7 | 0.3 | 0.1 | 0.6 | 0.2 | 0.3 | 0.5 | 0.3 | 0.4 | 0.5 | 0.5 | 0.1 | 0.1 | 0.4 | 0.3 | 0.3 | 0.5 | 0.3 | 0.3 | 0.3 | 0.8 | 0.3 | 0.1 |
| 7 | 2616 | 76.4 | 0.8 | 0.3 | 0.2 | 0.4 | 0.5 | 0.2 | 0.1 | 0.5 | 0.3 | 0.3 | 0.5 | 0.3 | 0.4 | 0.6 | 0.5 | 0.1 | 0.1 | 0.4 | 0.4 | 0.1 | 0.5 | 0.4 | 0.3 | 0.3 | 0.3 | 0.7 | 0.1 |
| 7 | 2536 | 69.7 | 0.1 | 0.1 | 0.1 | 0.4 | 0.7 | 0.2 | 0.1 | 0.6 | 0.3 | 0.4 | 0.5 | 0.3 | 0.5 | 0.5 | 0.6 | 0.3 | 0.1 | 0.3 | 0.2 | 0.3 | 0.2 | 0.2 | 0.6 | 0.4 | 0.1 | 0.8 | 0.1 |
| 7 | 2703 | 75.6 | 0.8 | 0.2 | 0.2 | 0.4 | 0.4 | 0.1 | 0.2 | 0.6 | 0.5 | 0.3 | 0.5 | 0.3 | 0.4 | 0.5 | 0.5 | 0.1 | 0.1 | 0.4 | 0.4 | 0.1 | 0.5 | 0.3 | 0.2 | 0.3 | 0.5 | 0.7 | 0.1 |
| 7 | 2675 | 70.9 | 0.8 | 0.2 | 0.1 | 0.5 | 0.5 | 0.3 | 0.1 | 0.6 | 0.2 | 0.3 | 0.6 | 0.3 | 0.5 | 0.5 | 0.6 | 0.3 | 0.4 | 0.3 | 0.3 | 0.3 | 0.1 | 0.2 | 0.5 | 0.3 | 0.2 | 0.3 | 0.1 |
| 7 | 2918 | 73.3 | 0.8 | 0.2 | 0.1 | 0.6 | 0.7 | 0.3 | 0.1 | 0.6 | 0.2 | 0.3 | 0.5 | 0.3 | 0.5 | 0.7 | 0.6 | 0.3 | 0.1 | 0.3 | 0.4 | 0.2 | 0.1 | 0.3 | 0.3 | 0.3 | 0.1 | 0.7 | 0.1 |
| 7 | 2703 | 75.6 | 0.8 | 0.2 | 0.2 | 0.4 | 0.4 | 0.1 | 0.2 | 0.6 | 0.5 | 0.3 | 0.5 | 0.3 | 0.4 | 0.5 | 0.5 | 0.1 | 0.1 | 0.4 | 0.4 | 0.1 | 0.5 | 0.3 | 0.2 | 0.3 | 0.5 | 0.7 | 0.1 |
| 7 | 2726 | 79.6 | 0.8 | 0.2 | 0.2 | 0.4 | 0.5 | 0.1 | 0.2 | 0.6 | 0.5 | 0.3 | 0.5 | 0.2 | 0.4 | 0.7 | 0.5 | 0.1 | 0.1 | 0.4 | 0.4 | 0.2 | 0.5 | 0.2 | 0.1 | 0.3 | 0.5 | 0.7 | 0.1 |
| 8 | 2779 | 74.9 | 0.8 | 0.3 | 0.1 | 0.3 | 0.6 | 0.4 | 0.1 | 0.6 | 0.3 | 0.2 | 0.5 | 0.4 | 0.2 | 0.6 | 0.5 | 0.1 | 0.2 | 0.4 | 0.3 | 0.2 | 0.3 | 0.3 | 0.2 | 0.3 | 0.8 | 0.6 | 0.1 |
| 8 | 2523 | 76.8 | 0.8 | 0.1 | 0.2 | 0.3 | 0.7 | 0.3 | 0.1 | 0.5 | 0.4 | 0.4 | 0.6 | 0.3 | 0.2 | 0.6 | 0.5 | 0.1 | 0.2 | 0.4 | 0.4 | 0.2 | 0.2 | 0.3 | 0.3 | 0.4 | 0.8 | 0.3 | 0.1 |

| | | | | | | | | | | | | | | | | | | | | | | | | | | | | | |
|---|---|---|---|---|---|---|---|---|---|---|---|---|---|---|---|---|---|---|---|---|---|---|---|---|---|---|---|---|---|
| 8 | 2740 | 77.9 | 0.8 | 0.2 | 0.2 | 0.4 | 0.6 | 0.3 | 0.1 | 0.6 | 0.3 | 0.2 | 0.7 | 0.3 | 0.4 | 0.6 | 0.5 | 0.1 | 0.2 | 0.4 | 0.3 | 0.2 | 0.3 | 0.3 | 0.2 | 0.3 | 0.8 | 0.4 | 0.1 |
| 8 | 2761 | 80.4 | 0.8 | 0.2 | 0.1 | 0.4 | 0.5 | 0.3 | 0.1 | 0.6 | 0.4 | 0.3 | 0.7 | 0.4 | 0.3 | 0.7 | 0.5 | 0.1 | 0.1 | 0.3 | 0.3 | 0.2 | 0.3 | 0.3 | 0.3 | 0.3 | 0.8 | 0.4 | 0.1 |
| 8 | 2692 | 67.9 | 0.7 | 0.1 | 0.3 | 0.4 | 0.7 | 0.3 | 0.1 | 0.6 | 0.5 | 0.2 | 0.5 | 0.3 | 0.1 | 0.7 | 0.4 | 0.1 | 0.1 | 0.3 | 0.5 | 0.2 | 0.2 | 0.4 | 0.1 | 0.4 | 0.8 | 0.5 | 0.1 |
| 8 | 2820 | 76.0 | 0.8 | 0.2 | 0.2 | 0.4 | 0.5 | 0.3 | 0.1 | 0.5 | 0.5 | 0.3 | 0.5 | 0.3 | 0.4 | 0.6 | 0.3 | 0.1 | 0.3 | 0.4 | 0.4 | 0.2 | 0.2 | 0.3 | 0.3 | 0.4 | 0.7 | 0.4 | 0.1 |
| 8 | 2831 | 67.2 | 0.1 | 0.5 | 0.3 | 0.3 | 0.5 | 0.4 | 0.1 | 0.6 | 0.2 | 0.4 | 0.5 | 0.3 | 0.4 | 0.1 | 0.6 | 0.3 | 0.1 | 0.5 | 0.2 | 0.3 | 0.1 | 0.4 | 0.5 | 0.4 | 0.2 | 0.8 | 0.2 |
| 8 | 2654 | 77.3 | 0.8 | 0.2 | 0.2 | 0.4 | 0.5 | 0.3 | 0.1 | 0.6 | 0.6 | 0.2 | 0.5 | 0.3 | 0.5 | 0.6 | 0.3 | 0.1 | 0.2 | 0.3 | 0.3 | 0.2 | 0.3 | 0.3 | 0.1 | 0.4 | 0.8 | 0.5 | 0.1 |
| 8 | 2577 | 79.4 | 0.8 | 0.2 | 0.3 | 0.3 | 0.6 | 0.2 | 0.1 | 0.6 | 0.6 | 0.2 | 0.5 | 0.3 | 0.4 | 0.6 | 0.4 | 0.1 | 0.1 | 0.3 | 0.3 | 0.2 | 0.2 | 0.3 | 0.2 | 0.5 | 0.8 | 0.5 | 0.1 |
| 8 | 2540 | 76.7 | 0.8 | 0.1 | 0.2 | 0.3 | 0.7 | 0.3 | 0.1 | 0.6 | 0.4 | 0.4 | 0.6 | 0.3 | 0.2 | 0.6 | 0.5 | 0.1 | 0.2 | 0.4 | 0.4 | 0.2 | 0.2 | 0.3 | 0.3 | 0.4 | 0.8 | 0.3 | 0.1 |
| 8 | 2486 | 77.0 | 0.8 | 0.2 | 0.3 | 0.3 | 0.7 | 0.3 | 0.1 | 0.6 | 0.6 | 0.2 | 0.5 | 0.3 | 0.4 | 0.5 | 0.3 | 0.2 | 0.1 | 0.3 | 0.4 | 0.2 | 0.2 | 0.3 | 0.1 | 0.5 | 0.8 | 0.4 | 0.1 |
| 8 | 2729 | 67.3 | 0.5 | 0.2 | 0.2 | 0.4 | 0.6 | 0.1 | 0.2 | 0.6 | 0.4 | 0.1 | 0.6 | 0.5 | 0.2 | 0.6 | 0.5 | 0.1 | 0.2 | 0.3 | 0.3 | 0.2 | 0.3 | 0.3 | 0.3 | 0.2 | 0.8 | 0.4 | 0.1 |
| 8 | 2648 | 78.3 | 0.7 | 0.1 | 0.1 | 0.3 | 0.6 | 0.5 | 0.1 | 0.6 | 0.3 | 0.2 | 0.7 | 0.3 | 0.4 | 0.5 | 0.5 | 0.1 | 0.2 | 0.3 | 0.1 | 0.3 | 0.4 | 0.3 | 0.3 | 0.3 | 0.8 | 0.4 | 0.1 |
| 8 | 2704 | 81.3 | 0.8 | 0.2 | 0.1 | 0.4 | 0.5 | 0.3 | 0.1 | 0.6 | 0.4 | 0.3 | 0.7 | 0.4 | 0.3 | 0.6 | 0.5 | 0.1 | 0.2 | 0.3 | 0.3 | 0.2 | 0.3 | 0.3 | 0.3 | 0.3 | 0.8 | 0.4 | 0.1 |
| 8 | 2845 | 70.7 | 0.8 | 0.1 | 0.2 | 0.4 | 0.7 | 0.3 | 0.1 | 0.6 | 0.4 | 0.3 | 0.5 | 0.4 | 0.1 | 0.6 | 0.5 | 0.1 | 0.2 | 0.2 | 0.4 | 0.2 | 0.4 | 0.4 | 0.2 | 0.3 | 0.8 | 0.4 | 0.1 |
| 8* DANTE #1 | 2652 | 82.1 | 0.8 | 0.2 | 0.2 | 0.3 | 0.6 | 0.2 | 0.1 | 0.6 | 0.4 | 0.1 | 0.6 | 0.3 | 0.4 | 0.7 | 0.5 | 0.1 | 0.2 | 0.4 | 0.3 | 0.2 | 0.4 | 0.3 | 0.3 | 0.3 | 0.8 | 0.4 | 0.1 |
| 8 | 2717 | 75.4 | 0.8 | 0.2 | 0.3 | 0.4 | 0.7 | 0.3 | 0.1 | 0.6 | 0.3 | 0.3 | 0.7 | 0.3 | 0.3 | 0.6 | 0.3 | 0.1 | 0.1 | 0.4 | 0.5 | 0.2 | 0.2 | 0.3 | 0.3 | 0.4 | 0.8 | 0.3 | 0.1 |
| 8 | 2725 | 78.8 | 0.8 | 0.2 | 0.2 | 0.3 | 0.6 | 0.3 | 0.1 | 0.6 | 0.3 | 0.2 | 0.6 | 0.3 | 0.4 | 0.6 | 0.5 | 0.1 | 0.2 | 0.4 | 0.4 | 0.2 | 0.4 | 0.3 | 0.3 | 0.3 | 0.8 | 0.3 | 0.1 |
| 8 | 2842 | 74.3 | 0.8 | 0.2 | 0.1 | 0.4 | 0.7 | 0.2 | 0.1 | 0.5 | 0.4 | 0.2 | 0.7 | 0.4 | 0.2 | 0.6 | 0.4 | 0.1 | 0.2 | 0.3 | 0.4 | 0.2 | 0.3 | 0.3 | 0.3 | 0.4 | 0.7 | 0.4 | 0.1 |
| 8 | 2812 | 80.5 | 0.8 | 0.3 | 0.1 | 0.3 | 0.5 | 0.4 | 0.1 | 0.6 | 0.4 | 0.3 | 0.6 | 0.3 | 0.3 | 0.8 | 0.4 | 0.1 | 0.1 | 0.3 | 0.4 | 0.2 | 0.4 | 0.3 | 0.4 | 0.3 | 0.6 | 0.4 | 0.1 |
| Uniform | 2478 | 58.3 | 0.3 | 0.3 | 0.3 | 0.3 | 0.3 | 0.3 | 0.3 | 0.3 | 0.3 | 0.3 | 0.3 | 0.3 | 0.3 | 0.3 | 0.3 | 0.3 | 0.3 | 0.3 | 0.3 | 0.3 | 0.3 | 0.3 | 0.3 | 0.3 | 0.3 | 0.3 | 0.3 |
| GAD-MALL #1 | 2378 | 75.0 | 0.1 | 0.3 | 0.2 | 0.3 | 0.7 | 0.3 | 0.2 | 0.1 | 0.1 | 0.2 | 0.7 | 0.2 | 0.7 | 0.1 | 0.7 | 0.2 | 0.7 | 0.3 | 0.2 | 0.1 | 0.1 | 0.3 | 0.7 | 0.3 | 0.1 | 0.3 | 0.2 |
| GAD-MALL #2 | 2408 | 73.9 | 0.1 | 0.3 | 0.2 | 0.3 | 0.7 | 0.3 | 0.2 | 0.3 | 0.1 | 0.2 | 0.7 | 0.2 | 0.7 | 0.1 | 0.7 | 0.2 | 0.7 | 0.3 | 0.2 | 0.3 | 0.1 | 0.2 | 0.7 | 0.3 | 0.1 | 0.3 | 0.2 |

978

**Supplementary Table 8: List of representative compositions of fcc CCAs predicted by DANTE**

| Fe (at. %) | Co (at. %) | Ni (at. %) | Al (at. %) | Zn (at. %) | Ir (at. %) | $E_f$ (Ry/site) | Mag ($\mu_B$/f.u.) | $\|\sigma_{xy}\|$ $(m\Omega\ cm)^{-1}$ | $\|\sigma_{xy}\|/\sigma_{xx}$ |
|---|---|---|---|---|---|---|---|---|---|
| 43.5 | 19.0 | 9.5 | 5.0 | 8.5 | 14.5 | 0.007 | 1.46 | 0.933 | 0.069 |
| 43.5 | 19.5 | 9.5 | 5.0 | 8.5 | 14.0 | 0.007 | 1.47 | 0.915 | 0.067 |
| 42.5 | 16.5 | 12.0 | 5.5 | 7.5 | 16.0 | 0.007 | 1.41 | 0.855 | 0.066 |
| 43.5 | 18.5 | 10.0 | 4.5 | 9.5 | 14.0 | 0.007 | 1.46 | 0.857 | 0.063 |
| 43.5 | 18.5 | 10.0 | 5.5 | 7.5 | 15.0 | 0.007 | 1.46 | 0.831 | 0.062 |
| 42.5 | 15.5 | 12.5 | 5.5 | 8.0 | 16.0 | 0.007 | 1.40 | 0.815 | 0.063 |
| 42.5 | 17.5 | 10.5 | 5.0 | 8.5 | 16.0 | 0.007 | 1.42 | 0.819 | 0.063 |
| 42.5 | 18.0 | 10.0 | 5.0 | 8.5 | 16.0 | 0.007 | 1.43 | 0.818 | 0.063 |
| 43.0 | 18.0 | 10.5 | 5.5 | 7.5 | 15.5 | 0.007 | 1.44 | 0.815 | 0.062 |

**Supplementary Table 9: List of representative compositions of bcc CCAs predicted by DANTE**

| Fe (at. %) | Co (at. %) | Ni (at. %) | Al (at. %) | Zn (at. %) | Ir (at. %) | Si (at. %) | $E_f$ (Ry/site) | Mag ($\mu_B$/f.u.) | $\|\sigma_{xy}\|$ $(m\Omega\ cm)^{-1}$ | $\|\sigma_{xy}\|/\sigma_{xx}$ |
|---|---|---|---|---|---|---|---|---|---|---|
| 63.5 | 0.5 | 0.5 | 9.0 | 8.0 | 18.5 | 0.0 | 0.002 | 1.72 | 0.993 | 0.093 |
| 67.0 | 0.5 | 0.5 | 0.0 | 15.0 | 13.5 | 3.5 | 0.005 | 1.79 | 0.963 | 0.082 |
| 61.5 | 0.5 | 0.5 | 0.0 | 19.0 | 16.0 | 2.5 | 0.006 | 1.67 | 0.924 | 0.085 |
| 67.5 | 0.5 | 0.5 | 0.0 | 14.0 | 13.5 | 4.0 | 0.004 | 1.80 | 0.957 | 0.082 |
| 59.0 | 0.5 | 0.5 | 0.0 | 21.5 | 16.5 | 2.0 | 0.006 | 1.60 | 0.901 | 0.085 |
| 69.5 | 0.5 | 0.5 | 0.0 | 13.5 | 12.0 | 4.0 | 0.004 | 1.83 | 0.970 | 0.079 |
| 59.0 | 0.5 | 0.5 | 0.0 | 20.5 | 17.0 | 2.5 | 0.006 | 1.61 | 0.891 | 0.085 |
| 69.5 | 0.5 | 0.5 | 0.0 | 14.5 | 11.5 | 3.5 | 0.004 | 1.83 | 0.977 | 0.077 |
| 60.5 | 0.5 | 0.5 | 0.0 | 18.5 | 16.5 | 3.5 | 0.006 | 1.65 | 0.886 | 0.084 |

**Supplementary Table 10: List of representative compositions of fcc CCAs predicted by MCMC**

| Fe (at. %) | Co (at. %) | Ni (at. %) | Al (at. %) | Pt (at. %) | Ir (at. %) | $E_f$ (Ry/site) | Mag ($\mu_B$/f.u.) | $\|\sigma_{xy}\|$ $(m\Omega\ cm)^{-1}$ | $\|\sigma_{xy}\|/\sigma_{xx}$ |
|---|---|---|---|---|---|---|---|---|---|
| 47.0 | 17.0 | 14.5 | 5.5 | 9.0 | 7.0 | 0.005 | 1.59 | 1.020 | 0.057 |
| 39.0 | 14.0 | 11.5 | 3.5 | 20.0 | 12.0 | 0.007 | 1.41 | 0.820 | 0.059 |
| 41.5 | 18.5 | 9.0 | 4.5 | 15.5 | 11.0 | 0.006 | 1.50 | 0.825 | 0.058 |
| 36.0 | 12.5 | 11.5 | 4.5 | 19.5 | 16.0 | 0.006 | 1.30 | 0.797 | 0.059 |
| 41.0 | 18.5 | 9.0 | 4.5 | 15.5 | 11.5 | 0.007 | 1.49 | 0.817 | 0.057 |
| 35.0 | 27.0 | 13.0 | 4.5 | 12.5 | 8.0 | 0.006 | 1.48 | 0.905 | 0.051 |

| | | | | | | | | | |
|---|---|---|---|---|---|---|---|---|---|
| 45.0 | 7.0 | 23.0 | 5.5 | 10.0 | 9.5 | 0.005 | 1.45 | 0.805 | 0.057 |
| 41.5 | 16.5 | 10.5 | 5.0 | 14.0 | 12.5 | 0.006 | 1.46 | 0.790 | 0.057 |
| 39.0 | 13.0 | 13.5 | 5.5 | 19.0 | 9.5 | 0.004 | 1.40 | 0.788 | 0.057 |

**Supplementary Table 11: List of representative compositions of bcc CCAs predicted by MCMC**

| Fe (at. %) | Co (at. %) | Al (at. %) | Pt (at. %) | Ir (at. %) | Zn (at. %) | Pd (at. %) | Mn (at. %) | Mg (at. %) | Rh (at. %) | Si (at. %) | $E_f$ (Ry/site) | Mag ($\mu_B$/f.u.) | $\|\sigma_{xy}\|$ $(m\Omega\ cm)^{-1}$ | $\|\sigma_{xy}\|$ $/\sigma_{xx}$ |
|---|---|---|---|---|---|---|---|---|---|---|---|---|---|---|
| 58.5 | 1.0 | 5.0 | 29.0 | 5.0 | 0.0 | 0.0 | 0.0 | 1.5 | 0.0 | 0.0 | 0.003 | 1.75 | 1.085 | 0.068 |
| 72.0 | 1.0 | 8.0 | 0.0 | 10.0 | 2.0 | 0.0 | 7.0 | 0.0 | 0.0 | 0.0 | 0.001 | 1.98 | 0.927 | 0.075 |
| 72.5 | 1.0 | 7.5 | 0.0 | 7.5 | 5.0 | 0.0 | 6.5 | 0.0 | 0.0 | 0.0 | 0.001 | 1.95 | 0.904 | 0.073 |
| 73.5 | 1.0 | 7.0 | 0.0 | 7.0 | 5.0 | 0.0 | 6.5 | 0.0 | 0.0 | 0.0 | 0.001 | 1.97 | 0.888 | 0.070 |
| 59.0 | 1.0 | 0.0 | 27.5 | 4.5 | 0.0 | 0.5 | 0.0 | 0.0 | 0.0 | 7.5 | 0.003 | 1.75 | 0.797 | 0.056 |
| 60.0 | 0.5 | 0.0 | 0.0 | 10.0 | 16.0 | 0.0 | 0.0 | 1.5 | 0.0 | 12.0 | 0.005 | 1.58 | 0.668 | 0.066 |
| 60.0 | 0.5 | 0.0 | 0.0 | 9.5 | 15.5 | 0.0 | 0.0 | 3.0 | 0.0 | 11.5 | 0.006 | 1.58 | 0.667 | 0.065 |
| 59.0 | 1.0 | 0.0 | 26.0 | 5.0 | 0.0 | 0.0 | 0.0 | 0.0 | 1.0 | 8.0 | 0.003 | 1.75 | 0.772 | 0.056 |
| 59.5 | 0.5 | 0.0 | 0.0 | 9.5 | 16.0 | 0.0 | 0.0 | 3.0 | 0.0 | 11.5 | 0.006 | 1.57 | 0.662 | 0.065 |

**Supplementary Table 12: The AHC of ferromagnetic fcc Fe43.5Co18.5Ni10Al4.5Zn9.5Ir14, Fe65Ir35 as well as bcc Fe61.5Co0.5Ni0.5Si2.5Zn19Ir16 and Fe80Ir20**

In this table, nvc and vc denote transport properties without and with vertex corrections, respectively. The unit of AHC ($\boldsymbol{\sigma_{xy}}$) is $(\mathrm{m}\Omega\ \mathrm{cm})^{-1}$

| | $\sigma_{xy}^{0}$ | $\sigma_{xy}^{1,I}$ | $\sigma_{xy}^{1,II}$ | $\sigma_{xy}$ |
|---|---|---|---|---|
| Fe43.5Co18.5Ni10Al4.5Zn9.5Ir14 (fcc, nvc) | 0.033 | 0.534 | 0.143 | 0.710 |
| Fe43.5Co18.5Ni10Al4.5Zn9.5Ir14 (fcc, vc) | 0.033 | 0.687 | 0.138 | 0.858 |
| Fe65Ir35 (fcc, nvc) | 0.026 | 0.407 | 0.101 | 0.534 |
| Fe65Ir35 (fcc, vc) | 0.026 | 0.459 | 0.103 | 0.588 |
| Fe61.5Co0.5Ni0.5Si2.5Zn19Ir16 (bcc, nvc) | 0.038 | 0.479 | 0.229 | 0.746 |
| Fe61.5Co0.5Ni0.5Si2.5Zn19Ir16 (bcc, vc) | 0.038 | 0.657 | 0.229 | 0.924 |
| Fe80Ir20 (bcc, nvc) | 0.040 | 0.749 | 0.254 | 1.043 |
| Fe80Ir20 (bcc, vc) | 0.040 | 0.826 | 0.256 | 1.122 |

**Supplementary Table 13: The calculated structure corresponding to each element**

The asteroid marks a meta-stable reference crystal structure.

| **Element** | Ti | Nb | Al | Ge | Co | Au | Pd | Ni | Zn |
|---|---|---|---|---|---|---|---|---|---|
| **Structure** | hcp | bcc | fcc | Diamond | hcp | fcc | fcc | fcc | hcp |
| **Element** | Ga | Mo | Cu | Pt | Sn | Cr | Mn | Mg | Si |

| | | | | | | | | |
|---|---|---|---|---|---|---|---|---|
| **Structure** | fcc* | bcc | fcc | fcc | Diamond | bcc | hcp* | hcp | Diamond |
| **Element** | Fe | Ru | Rh | Hf | Ta | W | Re | Ir | Bi |
| **Structure** | bcc | hcp | fcc | hcp | bcc | bcc | hcp | fcc | Bcc* |

**Supplementary Table 14: Metrics for *De novo* cyclic peptide binder design**

The highlighted one has the best target value.

| pdb | type | number | Target / Å$^2$ | SC | dSASA / Å$^2$ | pdb | Target / Å$^2$ | SC | dSASA / Å$^2$ |
|---|---|---|---|---|---|---|---|---|---|
| 1sfi | Nature | | 10.15 | 0.701 | 1448.61 | 1sld | 5.33 | 0.730 | 730.18 |
| | DANTE | 1 | 10.92 | 0.675 | 1618.02 | | 5.05 | 0.730 | 691.48 |
| | | 2 | 11.34 | 0.728 | 1558.72 | | **5.81** | **0.627** | **925.78** |
| | | 3 | **13.57** | **0.705** | **1925.38** | | 5.18 | 0.572 | 905.76 |
| | GD | 1 | 1.35 | 0.550 | 245.83 | | 3.69 | 0.602 | 613.97 |
| | | 2 | 10.70 | 0.742 | 1443.17 | | 3.52 | 0.513 | 687.00 |
| | | 3 | 9.81 | 0.641 | 1530.74 | | 4.75 | 0.529 | 897.69 |
| | MCMC | 1 | 10.58 | 0.655 | 1615.47 | | 3.72 | 0.461 | 807.31 |
| | | 2 | 11.50 | 0.751 | 1530.92 | | 4.72 | 0.519 | 910.51 |
| | | 3 | 9.59 | 0.705 | 1360.64 | | 3.49 | 0.752 | 464.51 |
| 1smf | Nature | | 7.50 | 0.746 | 1005.24 | 3p72 | **8.80** | **0.763** | **1152.96** |
| | DANTE | 1 | **8.89** | **0.685** | **1297.30** | | 8.51 | 0.735 | 1158.54 |
| | | 2 | 8.60 | 0.756 | 1137.36 | | 8.61 | 0.699 | 1231.85 |
| | | 3 | 8.24 | 0.652 | 1264.85 | | 8.21 | 0.589 | 1394.38 |
| | GD | 1 | 7.31 | 0.691 | 1058.40 | | 7.44 | 0.524 | 1420.02 |
| | | 2 | 6.00 | 0.570 | 1052.16 | | 6.28 | 0.547 | 1147.88 |
| | | 3 | 8.01 | 0.709 | 1130.17 | | 3.05 | 0.323 | 942.21 |
| | MCMC | 1 | 7.04 | 0.608 | 1157.19 | | 3.69 | 0.390 | 947.23 |
| | | 2 | 7.60 | 0.646 | 1177.31 | | 4.20 | 0.388 | 1083.41 |
| | | 3 | 3.88 | 0.512 | 756.18 | | 3.92 | 0.410 | 956.66 |
| 3zgc | Nature | | 5.96 | 0.713 | 835.77 | 4ib5 | 6.53 | 0.624 | 1046.26 |
| | DANTE | 1 | **6.55** | **0.728** | **899.68** | | **9.58** | **0.780** | **1228.46** |
| | | 2 | 5.46 | 0.615 | 887.05 | | 8.85 | 0.704 | 1257.73 |
| | | 3 | 5.90 | 0.650 | 907.43 | | 9.18 | 0.786 | 1168.05 |
| | GD | 1 | 4.54 | 0.544 | 834.52 | | 7.92 | 0.721 | 1099.24 |
| | | 2 | 0.71 | 0.198 | 358.21 | | 7.09 | 0.676 | 1048.96 |
| | | 3 | 4.68 | 0.718 | 652.10 | | 7.42 | 0.727 | 1021.51 |
| | MCMC | 1 | 2.17 | 0.439 | 494.78 | | 8.94 | 0.741 | 1206.37 |
| | | 2 | 2.45 | 0.386 | 634.29 | | 7.10 | 0.663 | 1070.63 |
| | | 3 | 4.34 | 0.472 | 919.38 | | 8.32 | 0.742 | 1120.35 |
| 4kel | Nature | | 11.69 | 0.777 | 1504.00 | 5h5q | 7.77 | 0.758 | 1024.75 |
| | DANTE | 1 | **13.45** | **0.752** | **1788.49** | | 7.77 | 0.780 | 996.43 |
| | | 2 | 12.98 | 0.707 | 1837.1 | | **8.50** | **0.701** | **1212.31** |
| | | 3 | 13.32 | 0.723 | 1843.51 | | 7.98 | 0.714 | 1117.44 |
| | GD | 1 | 7.17 | 0.642 | 1117.13 | | 4.20 | 0.594 | 707.79 |
| | | 2 | 9.85 | 0.708 | 1391.35 | | 5.86 | 0.720 | 813.39 |
| | | 3 | 7.37 | 0.656 | 1123.58 | | 6.20 | 0.579 | 1070.4 |
| | MCMC | 1 | 8.84 | 0.700 | 1262.96 | | 7.87 | 0.738 | 1066.42 |
| | | 2 | 12.36 | 0.740 | 1669.76 | | 7.29 | 0.702 | 1038.36 |
| | | 3 | 10.52 | 0.675 | 1559.2 | | 5.50 | 0.577 | 953.23 |
| 5tu6 | Nature | | **7.87** | **0.723** | **1089.15** | 6d40 | 9.58 | 0.726 | 1320.00 |
| | DANTE | 1 | 6.18 | 0.679 | 910.80 | | **11.01** | **0.729** | **1509.77** |
| | | 2 | 6.19 | 0.581 | 1065.00 | | 8.81 | 0.626 | 1407.85 |
| | | 3 | 7.05 | 0.679 | 1037.35 | | 10.56 | 0.758 | 1392.28 |
| | GD | 1 | 5.92 | 0.660 | 896.14 | | 9.69 | 0.747 | 1296.64 |
| | | 2 | 5.92 | 0.635 | 931.67 | | 7.89 | 0.666 | 1184.02 |

| | | | | | | | | | |
|---|---|---|---|---|---|---|---|---|---|
| | | 3 | 5.19 | 0.547 | 948.75 | | 7.55 | 0.760 | 993.12 |
| | MCMC | 1 | 5.22 | 0.552 | 945.71 | | 8.86 | 0.657 | 1349.15 |
| | | 2 | 5.07 | 0.542 | 936.06 | | 6.49 | 0.614 | 1057.11 |
| | | 3 | 5.58 | 0.574 | 972.75 | | 10.07 | 0.735 | 1370.57 |
| 6u6k | Nature | | **9.46** | **0.805** | **1175.96** | 6vxy | 10.28 | 0.749 | 1372.11 |
| | DANTE | 1 | 7.88 | 0.639 | 1234.42 | | 11.32 | 0.748 | 1512.91 |
| | | 2 | 6.37 | 0.684 | 931.47 | | 11.02 | 0.725 | 1520.40 |
| | | 3 | 7.26 | 0.677 | 1072.11 | | **11.69** | **0.689** | **1696.12** |
| | GD | 1 | 3.52 | 0.642 | 548.44 | | 9.78 | 0.685 | 1427.80 |
| | | 2 | 3.43 | 0.437 | 786.31 | | 8.38 | 0.730 | 1148.92 |
| | | 3 | 6.88 | 0.756 | 910.61 | | 11.39 | 0.727 | 1566.2 |
| | MCMC | 1 | 6.13 | 0.612 | 1001.18 | | 10.63 | 0.674 | 1576.99 |
| | | 2 | 7.00 | 0.647 | 1081.51 | | 9.16 | 0.675 | 1357.77 |
| | | 3 | 2.20 | 0.446 | 492.54 | | 8.81 | 0.66 | 1333.93 |
| 7ezw | Nature | | 8.80 | 0.804 | 1093.45 | 7k2j | 5.60 | 0.647 | 865.20 |
| | DANTE | 1 | 8.52 | 0.606 | 1404.56 | | 5.68 | 0.644 | 881.57 |
| | | 2 | 7.67 | 0.812 | 944.07 | | 5.08 | 0.704 | 721.39 |
| | | 3 | 8.49 | 0.638 | 1329.28 | | **6.32** | **0.662** | **955.64** |
| | GD | 1 | 3.69 | 0.644 | 573.23 | | 4.63 | 0.556 | 832.43 |
| | | 2 | 6.71 | 0.626 | 1072.26 | | 4.86 | 0.498 | 975.58 |
| | | 3 | **9.62** | **0.680** | **1414.16** | | 5.15 | 0.648 | 794.16 |
| | MCMC | 1 | 8.08 | 0.726 | 1112.81 | | 5.82 | 0.675 | 861.24 |
| | | 2 | 7.62 | 0.686 | 1110.58 | | 4.37 | 0.640 | 683.46 |
| | | 3 | 9.61 | 0.727 | 1322.61 | | 3.75 | 0.511 | 734.20 |

**Supplementary Table 15: Sequence for *De novo* cyclic peptide binder design**

The highlighted sequence has the best target value.

| pdb | type | number | sequence | pdb | sequence |
|---|---|---|---|---|---|
| 1sfi(14) | Nature | | GRCTKSIPPICFPD | 1sld(6) | CHPQFC |
| | DANTE | 1 | CFQNYPAFKGKHPK | | MSYMPC |
| | | 2 | SNMSVENGMVMWML | | **HMPGAH** |
| | | 3 | **TDMMYSPFKIWCME** | | HPWGCM |
| | GD | 1 | CDAGCKQEMGSRHV | | LPCGGK |
| | | 2 | PKCRDVTSEDPVII | | DCPCGV |
| | | 3 | CMPGDERESCAKCR | | WLPCCP |
| | MCMC | 1 | RDTWCNSDVCRRNM | | CCGPHG |
| | | 2 | LLVHGNNVAYCREK | | CDIHHP |
| | | 3 | FKECWCPVDVTDPP | | YCSDCK |
| 1smf(9) | Nature | | CTKSIPPEC | 3p72(11) | **CTERMALHNLC** |
| | DANTE | 1 | **CWEINSVMW** | | IVDMKSVEMLH |
| | | 2 | GHKCYSQLD | | QVRAPFSMMWY |
| | | 3 | HLPNWLRCQ | | IRRWAPFGSPM |
| | GD | 1 | RCVPERTPM | | HKENPFNKHDR |
| | | 2 | PTRCMDVPP | | NKTMKSCRLED |
| | | 3 | VPPPTRCMD | | DCNINRHCEIF |
| | MCMC | 1 | QDTPEPERV | | TSKLRDHTRHR |
| | | 2 | EGPARIREE | | QWRMLQAGNLN |
| | | 3 | GLHVTCQCR | | GSYRDQVRARM |
| 3zgc(7) | Nature | | GDEETGE | 4ib5(13) | GCRLYGFKIHGCG |
| | DANTE | 1 | **NAFTFIC** | | **HWKFIITETWHCC** |
| | | 2 | QSFLWLQ | | VTQYCRWKWISMH |
| | | 3 | ITKEMNS | | IWHYADIEGRMCK |
| | GD | 1 | EDPDEFP | | NCVCSEKQNPGYF |

| | | | | | |
|---|---|---|---|---|---|
| | | 2 | ETPAGCD | | LAAVMRGAKWTMD |
| | | 3 | EEVGNGD | | ETSKRKGGWKHYG |
| | MCMC | 1 | CNCGAEL | | NWKWVNSGQTQVM |
| | | 2 | GCRWWET | | HFKSENKKWVEET |
| | | 3 | TMDDNLN | | QYFKDQKHRGMFR |
| 4kel(14) | Nature | | GFCQRSIPPICFPN | 5h5q(13) | CRVDLQGWRRCRR |
| | DANTE | 1 | **WHSYLFYPVPFNWI** | | QQEGYINVAAEEA |
| | | 2 | TRHSPREENVWAFW | | **YLCWKQEKNCDMN** |
| | | 3 | SVSYKNWIFYSFLE | | IGRNNGKSGPARY |
| | GD | 1 | APITFELCHVMVID | | QYINRRKTTTQDH |
| | | 2 | LQQAGDIYYRCSEG | | AKKMGTISCDKKE |
| | | 3 | CKKLGLSHLLDREL | | RNPKESNEDWILL |
| | MCMC | 1 | KKQVQDIWIDCEPI | | EPNAPYAQKGDEG |
| | | 2 | GDMVYKSFLEWPPF | | CPKCKYIQRNSGP |
| | | 3 | CKKLGLSHLLDREL | | NTRPGKHFHAPET |
| 5tu6(7) | Nature | | **INPYLYP** | 6d40(14) | GRCYKSIPPICFPD |
| | DANTE | 1 | HHSSQDL | | **RWSGYMSYEHAGNS** |
| | | 2 | KRGWYIV | | TRPYCYYCGLRIQF |
| | | 3 | GKFNTFH | | MYALKTSHFNNDVL |
| | GD | 1 | SVFGSEC | | QFFSGKAEGDPDEK |
| | | 2 | CENGCHY | | PGETAWGKPVEIKE |
| | | 3 | HLCEGNC | | MKEDPEMVYCSGQQ |
| | MCMC | 1 | HDGNEQV | | CAQRDQMRGDVMRR |
| | | 2 | KGPWGTG | | DYCAPREGKNDGTR |
| | | 3 | PDNITLG | | CPVEPDGFVRWCKG |
| 6u6k(11) | Nature | | **WWIIPKVKKGC** | 6vxy(14) | GRGTKSIPPIAFPD |
| | DANTE | 1 | FRGKWKVWQEN | | RPRCIFCPKVDSLW |
| | | 2 | TMDRSQVNEWK | | HKPYCPEYSTHSFD |
| | | 3 | LWTKCIDFHIM | | **MDHQNAYGFNLWSR** |
| | GD | 1 | ETDPRIKNFGS | | MCWPTANEAWSEDE |
| | | 2 | EGGGRGLKEIM | | GEEAEVPVKCQPKA |
| | | 3 | KENNPHNRGGL | | QVKCVDRKTANKEM |
| | MCMC | 1 | KKCEDARKCQD | | GVSVKQPHTIESTV |
| | | 2 | KKGWSDLREAL | | KWAVCHGCRLHHEA |
| | | 3 | RYIATTKQQYR | | SPEPRMLAEKPPTQ |
| 7ezw(11) | Nature | | ACEMGFFQDCG | 7k2j(7) | GDPEAGE |
| | DANTE | 1 | YDSMNTVQHPG | | MNFGWSE |
| | | 2 | CYEWNVGPGNW | | LMHDKSQ |
| | | 3 | MWYKDFAKGKE | | **GFESSRE** |
| | GD | 1 | ADINDIANPCG | | EYGDGDE |
| | | 2 | DDPFPKGKDIS | | EEVPDWD |
| | | 3 | **HTDDGMTSPKH** | | QSAEDGE |
| | MCMC | 1 | NRVEGTIHVMN | | TPAEKVE |
| | | 2 | VETTNPLTCQP | | EDDPEMS |
| | | 3 | PLDLDNLNSGN | | GEIGPED |

**Supplementary Table 16: Percentile of high-quality solutions designed by three methods.**

| Methods | Percentile of high-quality solutions (ipTM>0.8) |
|---|---|
| **DANTE** | **27%** |
| MCMC | 7% |

| | |
|---|---|
| TuRBO5 | 0% |

# Supplementary References

1. D. Eriksson, M. Pearce, J. Gardner, R. D. Turner, M. Poloczek, Scalable global optimization via local Bayesian optimization. *Adv Neural Inf Process Syst* **32** (2019).
2. N. Hansen, S. D. Müller, P. Koumoutsakos, Reducing the time complexity of the derandomized evolution strategy with covariance matrix adaptation (CMA-ES). *Evol Comput* **11**, 1–18 (2003).
3. R. Storn, K. Price, Differential evolution–a simple and efficient heuristic for global optimization over continuous spaces. *Journal of global optimization* **11**, 341–359 (1997).
4. J. Liu, A. Moreau, M. Preuss, J. Rapin, B. Roziere, F. Teytaud, O. Teytaud, "Versatile black-box optimization" in *Proceedings of the 2020 Genetic and Evolutionary Computation Conference* (2020), pp. 620–628.
5. R. Munos, Optimistic optimization of a deterministic function without the knowledge of its smoothness. *Adv Neural Inf Process Syst* **24** (2011).
6. B. Kim, K. Lee, S. Lim, L. Kaelbling, T. Lozano-Pérez, "Monte carlo tree search in continuous spaces using voronoi optimistic optimization with regret bounds" in *Proceedings of the AAAI Conference on Artificial Intelligence* (2020)vol. 34, pp. 9916–9924.
7. L. Wang, R. Fonseca, Y. Tian, Learning search space partition for black-box optimization using monte carlo tree search. *Adv Neural Inf Process Syst* **33**, 19511–19522 (2020).
8. W. R. Gilks, S. Richardson, D. Spiegelhalter, *Markov Chain Monte Carlo in Practice* (CRC press, 1995).
9. M. Pincus, A Monte Carlo method for the approximate solution of certain types of constrained optimization problems. *Oper Res* **18**, 1225–1228 (1970).
10. C. Ying, A. Klein, E. Christiansen, E. Real, K. Murphy, F. Hutter, "Nas-bench-101: Towards reproducible neural architecture search" in *International Conference on Machine Learning* (2019), pp. 7105–7114.
11. G. E. Hinton, Improving neural networks by preventing co-adaptation of feature detectors. *arXiv preprint arXiv:1207.0580* (2012).
12. C. White, W. Neiswanger, Y. Savani, "Bananas: Bayesian optimization with neural architectures for neural architecture search" in *Proceedings of the AAAI Conference on Artificial Intelligence* (2021)vol. 35, pp. 10293–10301.
13. B. Letham, R. Calandra, A. Rai, E. Bakshy, Re-examining linear embeddings for high-dimensional Bayesian optimization. *Adv Neural Inf Process Syst* **33**, 1546–1558 (2020).
14. Z. Chen, Y. Jiang, Y.-T. Shao, M. E. Holtz, M. Odstrčil, M. Guizar-Sicairos, I. Hanke, S. Ganschow, D. G. Schlom, D. A. Muller, Electron ptychography achieves atomic-resolution limits set by lattice vibrations. *Science (1979)* **372**, 826–831 (2021).
15. P. D. Nellist, *Scanning Transmission Electron Microscopy: Imaging and Analysis* (Springer, 2011).
16. J. M. Cowley, A. F. Moodie, The scattering of electrons by atoms and crystals. I. A new theoretical approach. *Acta Crystallogr* **10**, 609–619 (1957).
17. J. Song, C. S. Allen, S. Gao, C. Huang, H. Sawada, X. Pan, J. Warner, P. Wang, A. I. Kirkland, Atomic Resolution Defocused Electron Ptychography at Low Dose with a Fast, Direct Electron Detector. *Sci Rep* **9**, 3919 (2019).
18. J. Madsen, T. Susi, The abTEM code: transmission electron microscopy from first principles [version 2; peer review: 2 approved] . *Open Research Europe* **1** (2021).
19. B. H. Savitzky, S. E. Zeltmann, L. A. Hughes, H. G. Brown, S. Zhao, P. M. Pelz, T. C. Pekin, E. S. Barnard, J. Donohue, L. Rangel DaCosta, E. Kennedy, Y. Xie, M. T. Janish, M. M. Schneider, P. Herring, C. Gopal, A. Anapolsky, R. Dhall, K. C. Bustillo, P. Ercius, M. C. Scott, J. Ciston, A. M. Minor, C. Ophus, py4DSTEM: A Software Package for Four-Dimensional Scanning Transmission Electron Microscopy Data Analysis. *Microscopy and Microanalysis* **27**, 712–743 (2021).
20. X. Xia, C. M. Spadaccini, J. R. Greer, Responsive materials architected in space and time. *Nat Rev Mater*, doi: 10.1038/s41578-022-00450-z (2022).

21. G. Strang, G. J. Fix, *An Analysis of the Finite Element Method* (Prentice-Hall, 1973).
22. N. J. Higham, *Accuracy and Stability of Numerical Algorithms* (Society for Industrial and Applied Mathematics, Second., 2002; https://epubs.siam.org/doi/abs/10.1137/1.9780898718027).
23. B. Peng, Y. Wei, Y. Qin, J. Dai, Y. Li, A. Liu, Y. Tian, L. Han, Y. Zheng, P. Wen, Machine learning-enabled constrained multi-objective design of architected materials. *Nat Commun* **14**, 6630 (2023).
24. N. K. Katiyar, K. Biswas, J.-W. Yeh, S. Sharma, C. S. Tiwary, A perspective on the catalysis using the high entropy alloys. *Nano Energy* **88**, 106261 (2021).
25. P. Mori-Sánchez, A. J. Cohen, W. Yang, Localization and Delocalization Errors in Density Functional Theory and Implications for Band-Gap Prediction. *Phys. Rev. Lett.* **100**, 146401 (2008).
26. Z. Rao, P.-Y. Tung, R. Xie, Y. Wei, H. Zhang, A. Ferrari, T. P. C. Klaver, F. Körmann, P. T. Sukumar, A. da Silva, others, Machine learning–enabled high-entropy alloy discovery. *Science (1979)* **378**, 78–85 (2022).
27. D. Raabe, J. R. Mianroodi, J. Neugebauer, Accelerating the design of compositionally complex materials via physics-informed artificial intelligence. *Nat Comput Sci* **3**, 198–209 (2023).
28. T. Suzuki, R. Chisnell, A. Devarakonda, Y.-T. Liu, W. Feng, D. Xiao, J. W. Lynn, J. G. Checkelsky, Large anomalous Hall effect in a half-Heusler antiferromagnet. *Nat Phys* **12**, 1119–1123 (2016).
29. R. Singha, S. Roy, A. Pariari, B. Satpati, P. Mandal, Magnetotransport properties and giant anomalous Hall angle in the half-Heusler compound TbPtBi. *Phys. Rev. B* **99**, 35110 (2019).
30. D. B. Miracle, O. N. Senkov, A critical review of high entropy alloys and related concepts. *Acta Mater* **122**, 448–511 (2017).
31. H. C. Robarts, T. E. Millichamp, D. A. Lagos, J. Laverock, D. Billington, J. A. Duffy, D. O'Neill, S. R. Giblin, J. W. Taylor, G. Kontrym-Sznajd, others, Extreme Fermi surface smearing in a maximally disordered concentrated solid solution. *Phys Rev Lett* **124**, 46402 (2020).
32. S. Mu, G. D. Samolyuk, S. Wimmer, M. C. Troparevsky, S. N. Khan, S. Mankovsky, H. Ebert, G. M. Stocks, Uncovering electron scattering mechanisms in NiFeCoCrMn derived concentrated solid solution and high entropy alloys. *NPJ Comput Mater* **5**, 1 (2019).
33. J. Kudrnovsk\`y, V. Drchal, F. Máca, I. Turek, S. Khmelevskyi, Electron transport in high-entropy alloys: Al x CrFeCoNi as a case study. *Phys Rev B* **100**, 14441 (2019).
34. D. Ködderitzsch, K. Chadova, H. Ebert, Linear response Kubo-Bastin formalism with application to the anomalous and spin Hall effects: A first-principles approach. *Phys Rev B* **92**, 184415 (2015).
35. S. H. Vosko, L. Wilk, M. Nusair, Accurate spin-dependent electron liquid correlation energies for local spin density calculations: a critical analysis. *Can J Phys* **58**, 1200–1211 (1980).
36. T. Fukushima, H. Akai, T. Chikyow, H. Kino, Automatic exhaustive calculations of large material space by Korringa-Kohn-Rostoker coherent potential approximation method applied to equiatomic quaternary high entropy alloys. *Phys Rev Mater* **6**, 23802 (2022).
37. L. Vitos, *Computational Quantum Mechanics for Materials Engineers: The EMTO Method and Applications* (Springer Science & Business Media, 2007).
38. Z. Szotek, B. L. Gyorffy, G. M. Stocks, W. M. Temmerman, Electron and electron-positron momentum distributions in concentrated random alloys. *Journal of Physics F: Metal Physics* **14**, 2571 (1984).
39. A. A. Vinogradov, Y. Yin, H. Suga, Macrocyclic Peptides as Drug Candidates: Recent Progress and Remaining Challenges. *J Am Chem Soc* **141**, 4167–4181 (2019).
40. P. Hosseinzadeh, P. R. Watson, T. W. Craven, X. Li, S. Rettie, F. Pardo-Avila, A. K. Bera, V. K. Mulligan, P. Lu, A. S. Ford, B. D. Weitzner, L. J. Stewart, A. P. Moyer, M. Di Piazza, J. G. Whalen, P. Jr. Greisen, D. W. Christianson, D. Baker, Anchor extension: a structure-guided approach to design cyclic peptides targeting enzyme active sites. *Nat Commun* **12**, 3384 (2021).
41. A. Zorzi, K. Deyle, C. Heinis, Cyclic peptide therapeutics: past, present and future. (2017).
42. E. Muratspahić, K. Deibler, J. Han, N. Tomašević, K. B. Jadhav, A.-L. Olivé-Marti, N. Hochrainer, R. Hellinger, J. Koehbach, J. F. Fay, M. H. Rahman, L. Hegazy, T. W. Craven, B. R. Varga, G. Bhardwaj, K. Appourchaux, S. Majumdar, M. Muttenthaler, P. Hosseinzadeh, D. J. Craik, M. Spetea, T. Che, D. Baker,

C. W. Gruber, Design and structural validation of peptide–drug conjugate ligands of the kappa-opioid receptor. *Nat Commun* **14**, 8064 (2023).

43. A. Leaver-Fay, M. Tyka, S. M. Lewis, O. F. Lange, J. Thompson, R. Jacak, K. W. Kaufmann, P. D. Renfrew, C. A. Smith, W. Sheffler, I. W. Davis, S. Cooper, A. Treuille, D. J. Mandell, F. Richter, Y.-E. A. Ban, S. J. Fleishman, J. E. Corn, D. E. Kim, S. Lyskov, M. Berrondo, S. Mentzer, kk Zoran Popovic, J. J. Havranek, J. Karanicolas, R. Das, J. Meiler, T. Kortemme, J. J. Gray, B. Kuhlman, D. Baker, P. Bradley, ROSETTA3: An Object-Oriented Software Suite for the Simulation and Design of Macromolecules. *Methods Enzymol*, doi: 10.1016/S0076-6879(11)87019-9 (2011).
44. T. Kosugi, M. Ohue, Design of Cyclic Peptides Targeting Protein–Protein Interactions Using AlphaFold. *Int J Mol Sci* **24** (2023).
45. A. Koide, S. Koide, Monobodies: antibody mimics based on the scaffold of the fibronectin type III domain. *Protein Engineering Protocols*, 95–109 (2007).
46. M. Gebauer, A. Skerra, Engineered protein scaffolds as next-generation therapeutics. *Annu Rev Pharmacol Toxicol* **60**, 391–415 (2020).
47. J. Jumper, R. Evans, A. Pritzel, T. Green, M. Figurnov, O. Ronneberger, K. Tunyasuvunakool, R. Bates, A. Ž\’\idek, A. Potapenko, others, Highly accurate protein structure prediction with AlphaFold. *Nature* **596**, 583–589 (2021).
